# The first two cell-fate decisions of preimplantation mouse embryo development are not functionally independent

**DOI:** 10.1038/srep15034

**Published:** 2015-10-13

**Authors:** Aleksandar I. Mihajlović, Vasanth Thamodaran, Alexander W. Bruce

**Affiliations:** 1 Laboratory of Developmental Biology & Genetics (LDB&G), Department of Molecular Biology, Faculty of Science, University of South Bohemia, Branišovská 31, 37005 České Budějovice, CZECH REPUBLIC; 2 Institute of Entomology Biology Centre of the Czech Academy of Sciences, Branišovská 31, 37005 České Budějovice, CZECH REPUBLIC

## Abstract

During mouse preimplantation embryo development, three distinct cell lineages are formed, represented by the differentiating trophectoderm (TE), primitive endoderm (PrE) and the pluripotent epiblast (EPI). Classically, lineage derivation has been presented as a two-step process whereby outer TE cells are first segregated from inner-cell mass (ICM), followed by ICM refinement into either the PrE or EPI. As ICM founders can be produced following the fourth or fifth cleavage divisions, their potential to equally contribute to EPI and PrE is contested. Thus, modelling the early sequestration of ICM founders from TE-differentiation after the fourth cleavage division, we examined ICM lineage contribution of varying sized cell clones unable to initiate TE-differentiation. Such TE-inhibited ICM cells do not equally contribute to EPI and PrE and are significantly biased to form EPI. This bias is not caused by enhanced expression of the EPI marker *Nanog*, nor correlated with reduced apical polarity but associated with reduced expression of PrE-related gene transcripts (Dab2 and Lrp2) and down-regulation of plasma membrane associated Fgfr2. Our results favour a unifying model were the three cell lineages are guided in an integrated, yet flexible, fate decision centred on relative exposure of founder cells to TE-differentiative cues.

The preimplantation period of embryogenesis encompasses the first cell-fate decisions of mammalian development and results in the derivation of three distinct cell lineages by the time of uterine implantation at the late blastocyst stage. Two of these lineages, the trophectoderm (TE) and primitive endoderm (PrE), are differentiating and ultimately yield extraembryonic tissues. The third epiblast (EPI) lineage is pluripotent and is a progenitor pool for all subsequently derived foetal cells^1^,^2^,^3^. Classically, this process of lineage separation has been presented as occurring in two distinct independent cell-fate decisions. The first decision involves the spatial separation of outer-residing TE progenitor cells from inner-cell mass (ICM) during the fourth/8- to 16-cell and fifth/16- to 32-cell cleavages, via so-called ‘asymmetric/differentiative’ cell-divisions, that are distinct from ‘symmetric/conservative’ divisions yielding two outer-cells^4^,^5^. Additionally, this spatial separation can occur when atypical apolar outer-cells are physically forced inside the embryo by neighbouring cells exhibiting pronounced apical-basolateral polarity^6^. The second decision is classically defined as involving gene expression refinements and active cell sorting within the ICM that ultimately results in EPI cells, residing deep within the ICM, and the PrE comprising a monolayer of blastocoel-facing cells at the surface of the ICM (reviewed in^1^,^2^,^3^,^7^). The collective result of much research into these two cell-fate decisions has led to the identification of key transcription-factor genes (*e.g. Tead4*^8^,^9^ and *Cdx2*^10^ in the TE, *Nanog*^11^ in EPI and *Gata6/4*^12^,^13^,^14^ and *Sox17*^15^ in the PrE) required to regulate the necessary gene expression patterns to support these emerging cell-types. Additionally, the differential activity of the hippo-signalling pathway in functionally regulating Tead4 output between outer and inner-cells^16^,^17^,^18^,^19^, and PrE promoting extra-cellular signalling provided by Fgf4 (fibroblast growth factor 4), via the Fgfr2 (fibroblast growth factor receptor 2) receptor in the ICM^20^,^21^, have also emerged as important mechanisms.

However, the classical view of a functionally independent ‘two-step’ model of cell-fate derivation has been questioned^2^,^22^,^23^. The keystone of such questioning has been the observation that in time-lapse cell lineage tracing experiments, the majority of inner-cells generated after the fourth cleavage division are biased to ultimately form EPI. Whereas, inner-cells generated during the fifth cleavage division are strongly biased towards PrE, with inner-cells derived by an infrequent third wave of asymmetric division, being invariably fated to PrE^22^. These data, although not universally accepted^24^,^25^,^26^, suggest that not all ICM cells are generated with equal potential and suggests that ancestral cell history plays a role during ICM cell lineage separation. Moreover, it implies that rather than being a ‘two-step’ process, the emergence of the three blastocyst cell lineages is more an inter-related continuum, whereby mechanisms influencing TE and ICM separation, in the ‘first cell-fate decision’, have knock-on consequences in the ICM during the ‘second cell-fate decision’. Expressed alternatively, cell-fate can be influenced by the timing of inner-cell derivation, ultimately resulting in three possible outcomes, TE, PrE and EPI. This ‘integrated cell-fate model’ postulates that the earliest possible sequestration of EPI progenitor cells to the ICM is required to maintain their pluripotent properties, whereas the extra developmental ‘time’ parental PrE progenitors reside on the outside of the embryo, receiving TE-differentiative and apical-basolateral polarisation cues^16^,^17^,^18^,^19^,^27^,^28^,^29^, prior to their internalisation during the fifth cleavage division, predisposes their ICM progeny to form PrE; a model sometimes called the ‘time-outside hypothesis’^23^,^30^,^31^ but herein referred to as the ‘integrated cell-fate model’.

Accordingly, to test the integrated cell-fate model, we report our experiments inhibiting TE cell-fate initiation within defined sub-populations of cells (*i.e.* clones) in the developing preimplantation mouse embryo, assaying the frequency at which such cell clones, within the ICM, are capable of contributing to PrE; therefore modelling the early removal of inner-cells derived during the fourth cleavage division from TE-promoting differentiative signals, such as that provided by inhibited hippo-signalling^16^,^17^,^18^,^19^. We show, from observing PrE/EPI contribution in clones of varying size, that TE-inhibited ICM cells preferentially contribute progeny to the EPI rather than PrE, in a statistically significant manner. Moreover, the biased contribution is not because of non-physiological inductions in the expression of the EPI associated gene *Nanog*, but rather correlates with reduced expression of PrE markers (*Dab2* and *Lrp2*). Additionally, we observe decreased expression of *Fgfr2* and down-regulation of Fgfr2 protein from the plasma membrane, within TE-inhibited clones. Our results indicate that the ability to initiate and respond to TE-differentiation cues/primes blastomeres to contribute future PrE progenitors and that preventing TE-differentiation favours eventual EPI formation. Consequently, the data are consistent with the integrated cell-fate model stating that the early removal of cells from TE-differentiation, by their internalisation at or shortly after the fourth cleavage, predisposes their progeny to populate EPI; whereas later internalisation resulting from the fifth cleavage, whereby the outer-residing parental cells are exposed to additional differentiative signals, such as inhibited hippo-signalling^17^, biases development towards PrE. However, it is not impossible for TE-inhibited cells to yield PrE progeny, suggesting the observed relationship is not rigid and reflects the remarkable regulative capacity of the developing embryo to respond to additional concurrent, and potentially stochastic, cell-fate inputs, possibly relating to overall ICM cell number.

## Results

ICM founder cells are generated during or shortly after the fourth (the 8- to 16-cell transition) and fifth cleavage (the 16- to 32-cell transition) divisions^4^,^6^. The time between the completion of these divisions is approximately twelve hours^32^, during which outer-residing 16-cell stage blastomeres remain apical-basolaterally polarised and exposed to TE-differentiative cues, such as suppressed hippo-pathway signalling, whilst apolar inner-cells are protected from TE-differentiation by active hippo-pathway signalling^16^,^17^,^18^,^19^,^27^,^28^. As these outer-residing blastomeres can also generate further ICM founders after the fifth cleavage, it is questionable whether ICM progenitors produced by the fourth and fifth cleavage divisions have equal potential to contribute to EPI and PrE^24^,^25^,^26^. In order to test if ICM cells are generated with equal potential, irrespective of the extent of TE induction their parental cells received, we assayed ICM lineage contribution of TE-inhibited cell clones in the embryo. We hypothesised if the extent of TE induction was unimportant for PrE differentiation in the ICM, TE-inhibited clones would not be impaired in their potential to contribute to PrE. Conversely, if being able to initiate TE-differentiation facilitates PrE differentiation, such clones would be disadvantaged in populating the PrE, therefore supporting the integrated cell-fate model.

### *Tead4* down-regulation using long dsRNA phenocopies the zygotic *Tead4* ^*−/−*^ knock-out

**Figure 1:**
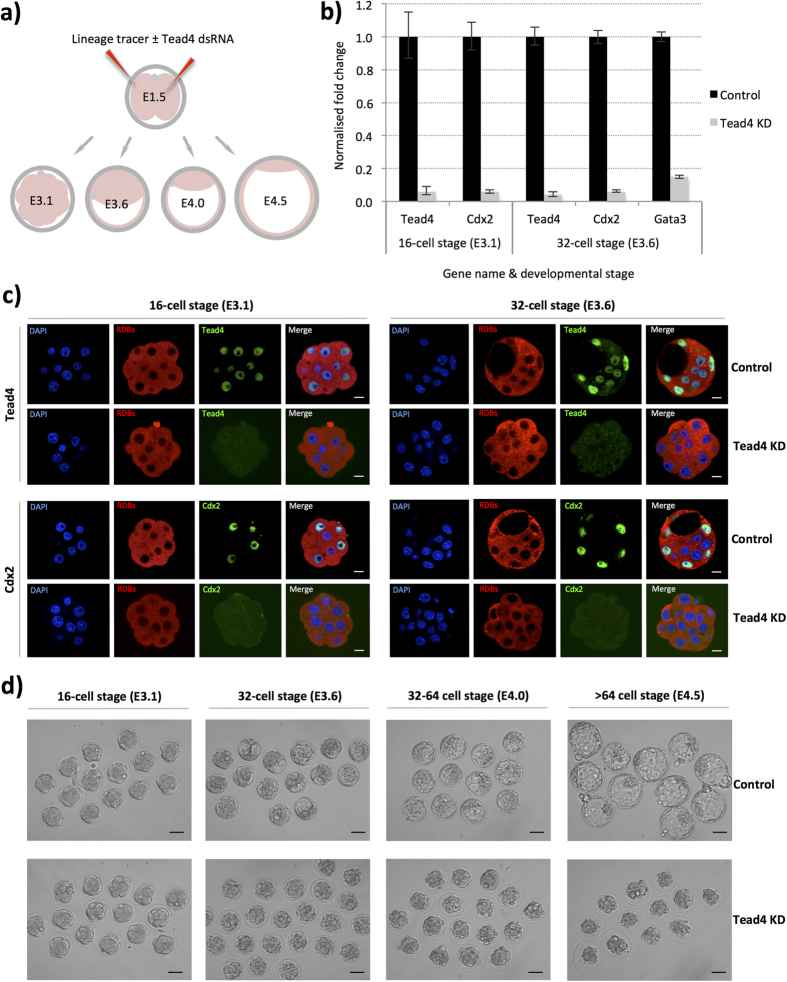
Long dsRNA mediated *Tead4* down-regulation phenocopies the zygotic *Tead4*^*−/−*^ null TE-deficit phenotype. (**a**) Schematic representation of experimental strategy. Embryos were microinjected with RDB injection marker (red) ± Tead4-dsRNA in both cells at the 2-cell stage (E1.5) and *in vitro* cultured until the mid-16-cell (E3.1), 32-cell (E3.6), 32–64-cell (E4.0) or >64-cell (E4.5) stages, prior to Q-RTPCR/microscopic analyses. (**b**) Q-RTPCR data detailing normalised average fold changes in mRNA expression of *Tead4, Cdx2* and *Gata3* in embryos microinjected with Tead4-dsRNA, relative to microinjection control embryos. Individual gene mRNA levels were normalised against Rpl23 and/or H2afz within control and experimental knockdown conditions and the fold change associated with *Tead4* KD calculated. Errors are given as s.e.m. n = at least 2 for biological replicates and 3 for technical replicates. (**c**) Representative single confocal immuno-fluorescence microscopy sections of embryos microinjected with RDB injection marker ± Tead4-dsRNA immuno-stained for Tead4 or Cdx2 protein (green) and DNA co-stained with DAPI (blue). RDB microinjection marker is visible (red). Scale bars = 10 μm. (**d**) Bright-field micrographs of control and Tead4-dsRNA microinjected embryos at various preimplantation stage developmental time-points in *in vitro* culture. Note that the *Tead4*-KD embryos fail to initiate blastocoel formation and starting from the E4.0 time-point exhibit cell death; a phenotype consistent with that observed in zygotic genetic *Tead4*^*−/−*^ null preimplantation embryos. Scale bars = 50 μm.

We employed an RNAi microinjection based strategy, targeting the *Tead4* gene, to inhibit TE-differentiation within defined cell clones. We reasoned clonal *Tead4* down-regulation would mimic the naturally occurring removal of cells from Tead4 regulation that occurs during their internalisation after the fourth cleavage division. We chose to target Tead4 as it is the earliest known transcription-factor to function in TE specification and its transcriptional activating properties are known to be regulated by hippo-signalling, thereby confining its regulatory output to polarised outer-cells^8^,^9^,^16^. Accordingly, we synthesised a *Tead4* specific long double-stranded RNA (Tead4-dsRNA) for use in single cell microinjection experiments that could be used to elicit TE-inhibited cell clones in the preimplantation mouse embryo. We first confirmed the efficacy of the construct by microinjecting recovered 2-cell (E1.5) stage embryos, in both blastomeres, with RDBs (rhodamine dextran conjugated beads, lineage tracer) ± the Tead4-dsRNA. As shown in Fig. 1b, we observed >95% reduced Tead4 mRNA expression, at the 16- (E3.1) and 32-cell (E3.6) stages, with accompanying undetectable levels of Tead4 protein, in Tead4-dsRNA injected embryos (*Tead4*-KD embryos) compared to microinjection controls (Fig. 1c); confirming the efficacy of the Tead4-dsRNA construct. We also observed robust reductions in the expression of two other TE-specific transcription factor genes known to function downstream of Tead4, namely *Cdx2*^10^ (Fig. 1b,c) and *Gata3*^33^,^34^ (Fig. 1b), demonstrating a successful block in TE-specification. Moreover, when we *in vitro* cultured such *Tead4*-KD embryos, they developed in-step with controls until the 32-cell (E3.6) stage but subsequently failed to initiate blastocoel formation and displayed considerable cell-death by the late blastocyst stage (E4.5); recapitulating the phenotype exhibited by genetically null *Tead4*^*−/−*^ zygotic knockout embryos^8^,^9^ (Fig. 1d).

### Clonal down-regulation of *Tead4* expression inhibits PrE formation

**Figure 2:**
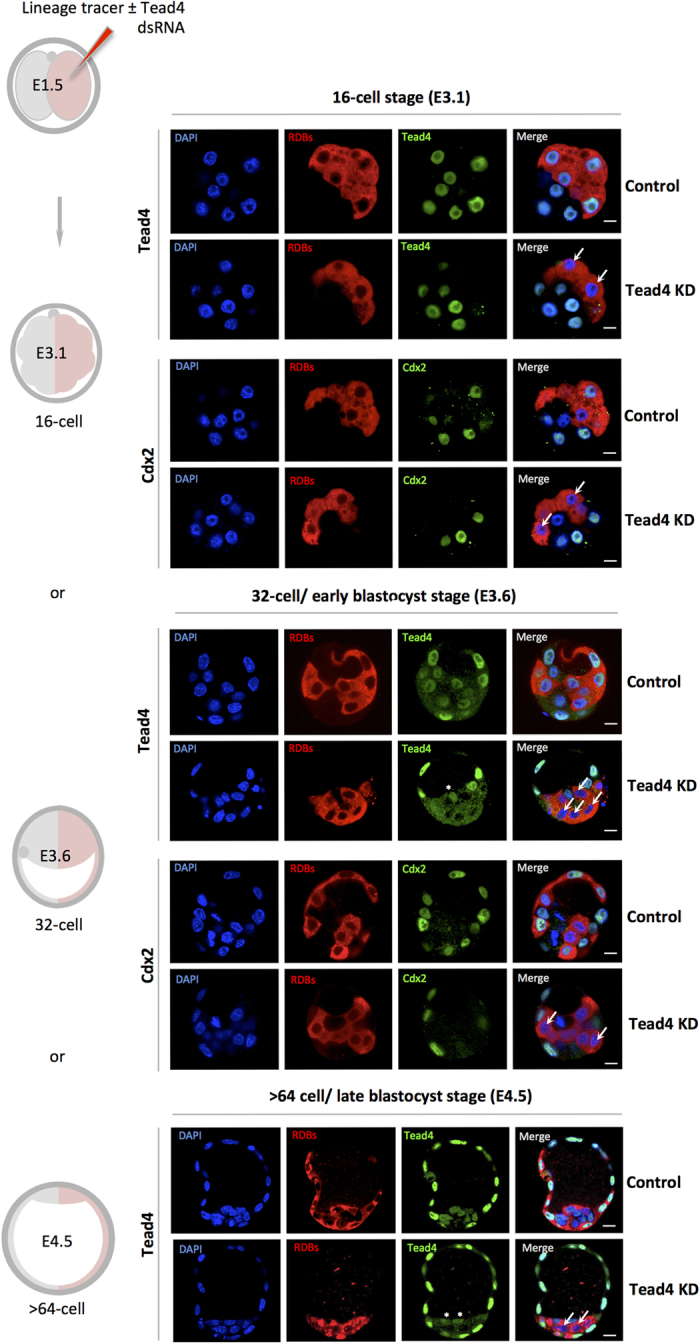
Clonal down-regulation of *Tead4* expression and TE-differentiation inhibition. A schematic of experimental strategy to effect clonal *Tead4* knockdown (KD) and TE-inhibition in one-half of the embryo using microinjected RDBs ± Tead4-dsRNA (see materials and methods) is given on the left. Representative single z-plane confocal micrographs of control and *Tead4*-KD embryos at either the mid-16-cell (E3.1), 32-cell (E3.6) or >64-cell (E4.5) stages immuno-stained for Tead4 or Cdx2 (green) are given. Cells derived from the microinjected 2-cell stage clone are distinguishable by the co-injected RDB fluorescence (red). DNA counter-stain (blue) is also shown. In merged images the arrows denote cells exhibiting a lack of Tead4 or Cdx2 expression in the *Tead4*-KD microinjected cell clone, thus confirming the efficacy and the functional and clonal inhibition of TE-differentiation by Tead4-dsRNA until the late blastocyst stage (E4.5). ICM cells not from the microinjected clone, expressing Tead4 protein are marked with asterisks (in Tead4 alone micrographs). Note, in contrast to global *Tead4*-KD embryos (Fig. 1), such clonal *Tead4*-KD embryos initiate blastocoel formation in a manner indistinguishable from control microinjected embryos. Scale bars = 10 μm.

We next asked what would be the consequence for PrE derivation in a clone of *Tead4*-KD cells in the developing embryo? First, we microinjected 2-cell (E1.5) stage embryos in one blastomere with RDBs ±Tead4-dsRNA, to elicit a fluorescently marked and TE-inhibited clone comprising half the embryo. As shown in Fig. 2, we observed complete down-regulation of Tead4 and Cdx2 protein, only within the cells derived from the Tead4-dsRNA microinjected blastomere and throughout the preimplantation developmental period; confirming our clonal TE-inhibition approach. However, in contrast to the global down-regulation of *Tead4* (Fig. 1), clonal *Tead4*-KD embryos were able to initiate appropriate blastocoel formation, illustrating the regulative capacity of the preimplantation mouse embryo.

**Figure 3:**
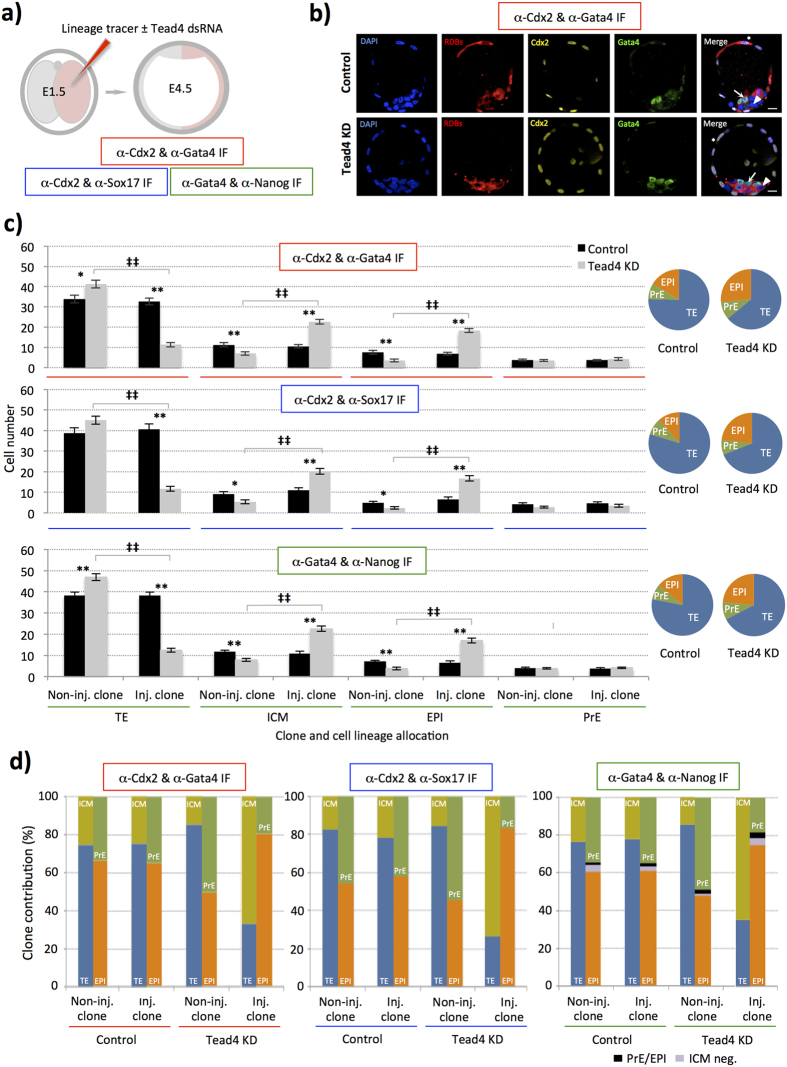
Clonal inhibition of TE-differentiation preferentially biases cells to EPI rather than PrE fates. (**a**) Experimental strategy to effect clonal *Tead4*-KD and TE-inhibition in one-half of the embryo and assess cell lineage allocation in late blastocysts (E4.5) via immuno-fluorescence detection of marker gene expression, using; i) Cdx2 (TE) & Gata4 (late PrE) - red, ii) Cdx2 & Sox17 (early PrE)—blue, and iii) Gata4 & Nanog (EPI)—green, (*n.b.* inner-cells devoid of either lineage marker in i) and ii) were classified as EPI and outer cells devoid of immuno-reactivity in iii) were designated as TE). (**b**) Representative single z-plane confocal micrographs of microinjection control and clonal *Tead4*-KD late blastocyst (E4.5) embryos immuno-stained for Cdx2 (pseudo-coloured yellow) and Gata4 (green) protein expression. Progeny of the microinjected cell are distinguishable by co-injected RDB fluorescence (red). DNA is counterstained with DAPI (blue). Merged image asterisks represent exemplar cells classified in our analyses as TE, arrows as PrE cells and arrow-heads as EPI. Note similar exemplar micrographs for the alternative immuno-staining regimes are given in Supplementary Figs S3 and S4. Scale bars = 10 μm. (**c**) Average number of cells from either non-microinjected or microinjected cell clones contributing to late blastocyst (E4.5) lineages, in control and clonal *Tead4*-KD embryos, immuno-stained in each of the three regimes outlined in a). Error bars represent s.e.m; *^/^** and ^‡/‡‡^ denote statistically significant differences between equivalent cell clones of control and clonal *Tead4*-KD embryos, or between cell clones within control and clonal *Tead4*-KD embryo groups, respectively (p < 0.05 and p < 0.005, 2-tailed student t-tests). The relative average percentage contribution of total cell number to each late blastocyst lineage in both control- and *Tead4*-KD embryos is also provided as a pie-chart. (**d**) Averaged percentage contribution of non-microinjected and microinjected cell clones, of control and clonal *Tead4*-KD embryos, immuno-stained according to the three regimes outlined in a), between the TE (blue) & ICM (yellow) and the PrE (green) & EPI (orange) of analysed late (E4.5) blastocysts. In the anti-Gata4/Nanog immuno-stained embryo groups, the contribution of ICM cells either positive or negative for both PrE and EPI marker gene expression are shown in black and violet, respectively. Overall, for control embryos n = 24, 13 & 25 and for clonal *Tead4*-KD embryos n = 24, 9 & 23 in each of the three immuno-staining regimes outlined in a), respectively. Note, further analysis and variant presentation of the above data is provided in Supplementary Figs S2–S4 and Supplementary Tables ST3–ST8.

We next assayed the frequency that *Tead4*-KD cells contributed to each late blastocyst (E4.5) cell lineage, compared with the non-injected clone and the equivalent clone from control embryos in which RDBs alone had been microinjected (Fig. 3). It is important to note that blastocyst lineage contribution and the incidence of apoptosis in such control embryos were statistically identical to that observed in a second group of control embryos that had been similarly microinjected at the 2-cell stage with RDBs plus a GFP specific dsRNA (GFP-dsRNA; lacking an endogenous mouse mRNA target—Supplementary Fig. S1 and Supplementary Tables ST1 & ST2). The lack of any statistically significant difference between these two groups, confirmed the appropriateness of using the RDB alone control in all the subsequently described assays.

Therefore, lineage contribution was assayed in RDB alone microinjected control and clonal *Tead4*-KD late blastocysts (E4.5) that had been double immuno-stained for either i) Gata4, a PrE marker^35^, and Cdx2, a marker of TE cells^10^, or ii) Sox17, an early PrE marker^15^,^22^, and Cdx2 (under each immuno-staining protocol, ICM cells devoid of either marker were classified as EPI, as ICM lineage segregation is reported to be completed by this time^36^), or, iii) Gata4 and Nanog, an EPI marker^11^, (thus directly assaying both ICM cell lineages whilst designating outer cells negative for either marker as TE). Further comprehensive analyses relating to the relative spatial location of cell clones, ICM versus outer-TE location, and cells with fragmented nuclei typical of apoptosis were also undertaken. Note that each of the three respective immuno-staining regimes described above were associated with dedicated and matched control and *Tead4*-KD embryo groups that were microinjected during the same experimental sessions. Accordingly, n = 24, 13 and 25 for RDB alone control groups and n = 24, 9 and 23 for *Tead4*-KD embryo groups per respective immuno-staining regime. Figure 3 summarises these data and Supplementary Figs S2, S3, and S4 provide expanded summaries from each immuno-staining method used. Supplementary Tables ST3, ST4 and ST5 provide individual embryo data for each of immuno-staining group and data relating to apoptosis can be found in Supplementary Fig. S5 and Supplementary Tables ST6, ST7 and ST8.

Our analyses showed that whilst clonal *Tead4*-KD embryos were able to develop into morphologically normal (E4.5) late blastocysts (Fig. 3b), the clonal origin of these cells, irrespective of how they were assayed in the differing immuno-staining regimes, were not equal. Specifically, *Tead4*-KD clones preferentially and significantly contributed to the ICM versus the outer TE with partial compensation coming from the non-microinjected clone (Fig. 3c); although *Tead4*-KD embryos presented with significantly less TE and more ICM than control embryos (Fig. 3c and Supplementary Figs S2–S4 and Supplementary Tables ST3–ST5). Cells derived from the Tead4-dsRNA microinjected clone that remained in an outer TE position, did not express Cdx2 protein (with very few isolated exceptions detailing very weak anti-Cdx2 immuno-fluorescence) and were almost exclusively spatially restricted to the polar TE, overlying the ICM, rather than mural TE surrounding the expanding blastocoel. Albeit marked, this phenotype is entirely consistent with the well-characterised *Tead4* role in TE-differentiation^16^. However, unlike the situation in control embryos, ICM cell lineage segregation was also unequal in *Tead4*-KD embryos, with the Tead4-dsRNA microinjected cell clones preferentially and significantly contributing to EPI rather than PrE (Fig. 3c). Whilst the ICMs of *Tead4*-KD embryos were significantly larger than control embryos, the number of PrE cells remained equal in both groups, with all the extra *Tead4*-KD ICM cells contributing to increased EPI cell number (Fig. 3c and Supplementary Figs S2–3 and Supplementary Tables ST3–ST5). Moreover, the smaller non-microinjected ICM clone in TE-inhibited embryos (for example comprising 7.0 ± 0.8 cells versus 22.6 ± 1.1 cells for the microinjected clone in embryos immuno-stained with Gata4/Cdx2—Supplementary Fig. S2 and Supplementary Table ST3), contributed a statistically equal number of PrE cells than the microinjected clone (equating, in the Gata4/Cdx2 immuno-stained example, but a trend repeated in the other staining groups, to 50.3% of its overall size, versus 19.0% in the microinjected *Tead4*-KD ICM clone—Fig. 3d), emphasising the bias within the microinjected *Tead4*-KD clone against contributing to the PrE, despite forming the majority of the total cell number of the ICM. We also noted significant reductions in total cell number in *Tead4*-KD embryos (Supplementary Figs S2–S4 and Supplementary Tables ST3–ST5) accountable for by increased apoptosis within outer-residing cells of the microinjected clone, presumably unable to adapt to a spatial location requiring TE-differentiation (Supplementary Fig. S5 and Supplementary Tables ST6–ST8).

Overall, we found that individual ICM cells from either the non-microinjected and microinjected TE-inhibited clones of *Tead4*-KD embryos did not segregate between the EPI and PrE in a manner that would be consistent with them being generated with equal potential. Rather TE-inhibited ICM cells preferentially allocate to the EPI, with the non-microinjected cell clone contributing a greater proportion of its cells to the PrE. Importantly, this result was consistently observed irrespective of the combination of specific antibody markers used to assay the emerging late blastocyst (E4.5) cell lineages, thus validating the functional relevance of the ICM allocation phenotype observed. It has previously been reported that the ICMs of late blastocyst (E4.5) stage embryos can present with cells expressing neither an EPI or PrE marker^21^,^35^. Consistent with such reports we also observed incidences, using the anti-Gata4/Nanog antibody combination, of late blastocyst (E4.5) stage ICM cells that were either negative or positive for both ICM markers, albeit at very low frequencies and in both control and *Tead4*-KD embryo groups (on average less than one cell per embryo in both conditions—Fig. 3d, Supplementary Fig. S4 and Supplementary Table ST5). Although there was a statistically significant increased frequency of cells exhibiting negative immuno-fluorescence for each marker in the microinjected clone of *Tead4*-KD embryos, when compared with both the non-microinjected clone or the equivalent clone of control embryos, it was in overall numbers extremely modest (0.9 ± 0.2 cells per embryo versus 0.0 and 0.3 ± 0.1, respectively). Moreover, it was insufficiently large to explain the increased contribution of cells classified as EPI in embryos that were assessed by immuno-fluorescent staining for either Gata4/Cdx2 or Sox17/Cdx2 (Fig. 3 and Supplementary Figs S2 and S3 plus Supplementary Tables ST3 and ST4) as being derived from unspecified ICM cells.

We therefore interpret the collective sum of these experimental datasets as demonstrating that the clonal inhibition of TE cell-fate biases ICM progeny towards EPI rather than PrE formation, albeit with a very small number of ICM cells remaining uncommitted to either lineage. Moreover, this correlates with TE-differentiation potentiating later PrE differentiation, as suggested by the integrated cell-fate model. However, inhibition of TE-differentiation is not an impermeable block to future PrE formation, as cells derived from the TE-inhibited clone can populate the PrE but at much reduced frequency.

### TE-inhibition within smaller sized cell clones also inhibits PrE formation

**Figure 4:**
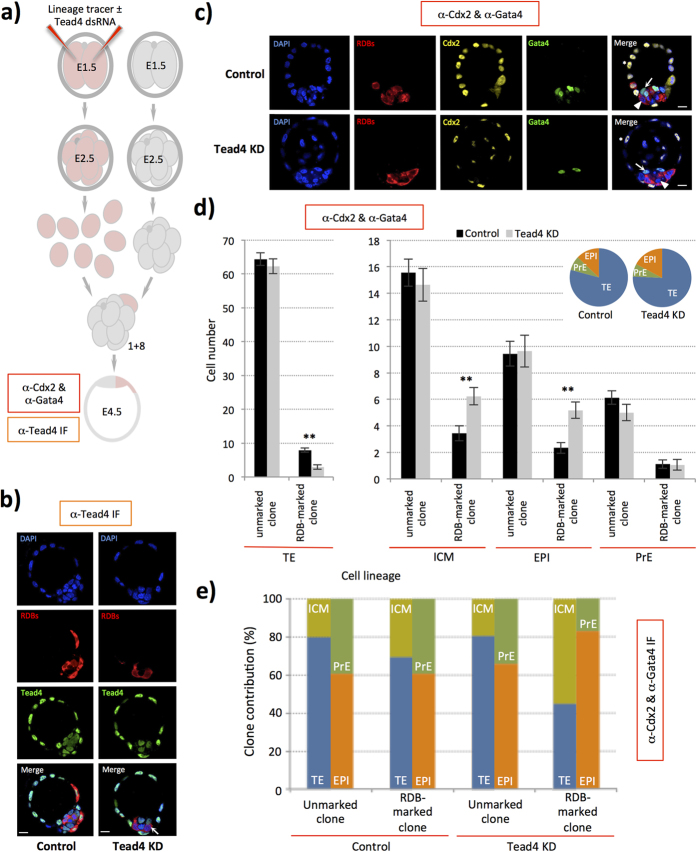
TE-inhibition within small chimeric ICM clones also biases against ultimate PrE cell-fate. (**a**) Experimental strategy to generate embryo chimeras containing marked TE-inhibited cells equivalent to one ninth of the embryo and to asses late blastocyst (E4.5) lineage formation, via immuno-fluorescent staining for Cdx2 and Gata4 (*n.b.* inner-cells devoid of either lineage marker were classified as EPI and immuno-staining using anti-Tead4 antibody was also used to confirm *Tead4* KD in the marked chimeric clones). (**b**) Representative single z-plane confocal micrographs of control and *Tead4*-KD clone containing chimeras immuno-stained for Tead4 (green) expression. In the merged image, arrows denote cells not expressing detectable levels of Tead4 derived from original *Tead4*-KD donor blastomere (**c**) Further, representative single z-plane confocal micrographs of late blastocyst (E4.5) chimeras immuno-stained for Cdx2 (pseudo-coloured yellow) and Gata4 (green). Merged image asterisks represent exemplar cells classified in our analyses as belonging to the TE, arrows PrE and arrow-heads EPI. In (**b**,**c**) cells deriving from donor blastomeres, themselves originally derived from control or Tead4-dsRNA microinjected 2-cell (E1.5) stage embryos, within chimeras are distinguishable by co-injected RDB (red). DNA is counterstained (DAPI, blue). Scale bars = 10 μm. (**d**) Average number of cells from either RDB-marked or non-marked cell clones in TE lineage (left) and ICM, EPI and PrE lineages (right), in control and *Tead4*-KD-chimeras; error bars represent s.e.m and *^/^** denote statistically significant differences between equivalent cell clones of control- and *Tead4*-KD-chimeras (p < 0.05 and p < 0.005, respectively - 2-tailed student t-tests). Inset pie-charts denote the relative average contribution of cells within each embryo group to each of the three late blastocyst (E4.5) lineages. (**e**) Averaged percentage contribution of unmarked and RDB-marked cell clones, of control and clonal *Tead4*-KD chimeric embryos, immuno-stained for Cdx2 and Gata4, between the TE (blue) & ICM (yellow) and the PrE (green) & EPI (orange) of analysed late blastocysts (E4.5). Overall, for control chimeras n = 30 and for clonal *Tead4*-KD chimeras n = 17. Note, further analysis and variant presentation of the above data is provided in Supplementary Fig. S7 and Supplementary Tables ST11 and ST12.

We next created embryos containing smaller sized TE-inhibited clones to assay if the observed bias against PrE contribution, at the late blastocyst (E4.5) stage, would persist. This was because, the above described experiments yielded embryos with relatively larger ICMs (*e.g. Tead4*-KD; 29.6 ± 1.5, versus controls; 21.8 ± 1.0 cells, in embryo groups immuno-stained for Gata4/Cdx2 - Supplementary Fig. S2 and Supplementary Table ST3) and we wanted to exclude the possibility that E4.5 stage late blastocysts could only support a finite number of PrE cells and that our results were not due to excessive developmental regulation (*i.e.* that once a finite number of PrE cells had been specified, any extra ICM cells would be defaulted to form EPI). Our first approach involved the direct microinjection of single individual blastomeres of mid-4-cell stage (E2.0) embryos, causing *Tead4-*KD in one quarter of the embryo (Supplementary Fig. S6 and Supplementary Table ST9, for apoptosis data see Supplementary Fig. S5 and Supplementary Table ST10; n = 19 control embryos and n = 29 *Tead4*-KD embryos, immuno-stained for Gata4 and Cdx2). We observed the same bias, as for the above described 2-cell microinjections, in the *Tead4*-KD microinjected cell clone. However, the average size of such *Tead4*-KD embryo ICMs was still greater than that observed in control embryos. Therefore, we adopted another strategy to characterise late blastocyst (E4.5) cell lineage contribution of still smaller clones of TE-inhibited cells, involving the creation of chimeric embryos from non-compacted 8-cell stage (E2.5) embryos aggregated with single developmentally matched donor blastomeres in which *Tead4* expression was down-regulated. Such chimeras contained marked TE-inhibited clones (*Tead4*-KD-chimeras) equivalent to one-ninth of their initial total cell number and when *in vitro* cultured to the last blastocyst stage were morphologically indistinguishable from control-clone containing chimeras (referred to as control-chimeras, see summarised data in Fig. 4, expanded in Supplementary Fig. S7 with Supplementary Tables ST11 and ST12 documenting individual embryo allocation and apoptosis data, respectively; n = 30 control-chimeras and n = 17 *Tead4*-KD-chimeras, immuno-stained for Gata4/Cdx2). Importantly, such *Tead4*-KD-chimeras had a total number of ICM cells statistically equal to that observed in control–chimeras (20.9 ± 1.0 versus 19.0 ± 0.9 cells, respectively) and were more similar in size to the ICMs of the non-perturbed *in vitro* cultured control blastocysts (22.4 ± 1.5 cells—Supplementary Table ST13). When we assayed late blastocyst (E4.5) cell lineage contribution, we found that the marked TE-inhibited clone of *Tead4*-KD-chimeras was not only biased to populate the ICM, it was also biased in a statistically significant manner to contribute to the EPI rather than the PrE. Therefore, we found that the inhibition of TE-differentiation within discreet small clones, within ICMs of more physiological appropriate size, also biases cell-fate against PrE formation. Thus, demonstrating that the observed anti-PrE biases observed in TE-inhibited ICM residing clones, derived from any of the three experimental strategies described, are not simply a function of generating unusually large ICMs. Moreover, the data reflect unequal cell-fate potential between such TE-inhibited clones when compared with non-manipulated, non-TE-inhibited, ICM cells of the same embryo. Expressed alternatively, clonal inhibition of TE-differentiation inhibits the progeny of that clone to differentiate towards a PrE cell-fate, when competing with otherwise unperturbed cells outwith the clone.

### Molecular characterisation of attenuated PrE formation in TE-inhibited cell clones

**Figure 5:**
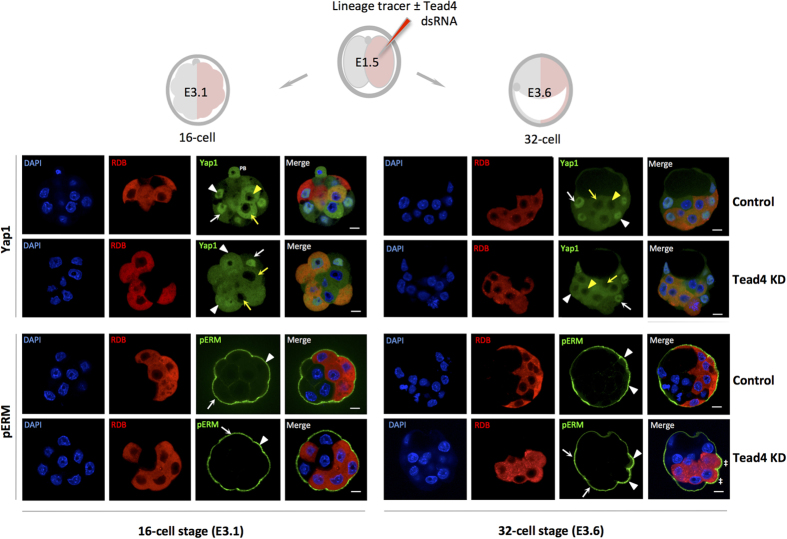
Clonal TE-inhibition causes outer-cell Yap1 mis-localisation and enhanced 32-cell stage apical polarity. A schematic of experimental strategy to assay, via confocal immuno-staining, Yap1 and phospho-ezrin/radixin/moesin (pERM) expression in TE-inhibited clones (comprising half the embryos cells) at the mid-16-cell (E3.1) or 32-cell (E3.6) stages is shown (top). Lower panels; representative single confocal z-plane micrographs of control and *Tead4*-KD embryos immuno-stained for Yap1 (anti-sera does not discriminate between phosphorylated or non-phosphorylated forms) and pERM expression at the mid-16-cell and 32-cell stages (both in green). DNA DAPI counter-stain (blue) and RDB marked microinjected clones (red) are also shown. Arrows and arrow-heads highlight exemplar Yap1/pERM protein expression within non-microinjected and microinjected clones respectively. In Yap1 images the white arrows or arrow-heads indicate staining in outer-cells and yellow variants in inner-cells (second polar body = ‘PB’). Double crosshairs denote outer 32-cell stage cells in the *Tead4*-KD group exhibiting atypical morphology with enhanced apically localised pERM immuno-staining (also seen in Prkcz and Pard6b immuno-staining—Supplementary Fig. S9). Scale bars = 10 μm.

The extent of 8-cell stage blastomere, and subsequent outer-cell, apical-basolateral polarisation has previously been correlated with cell-division orientation and outer-cell engulfment, whereby reduced polarity is associated with inner-cell generation and active hippo-signalling^6^,^28^. We therefore assayed for apical polarisation and hippo-signalling deficits within TE-inhibited cell clones using previously described anti-phospho-ezrin/radixin/moesin (pERM) and Yap1 antibodies^6^,^19^, respectively. However, we observed no apical polarisation defects by the mid-16 cell (E3.1) stage (Fig. 5) although we sometimes documented outer mid-16-cell stage blastomeres with substantially reduced apical pERM (not correlating with any clone) at frequencies similar to recent reports^6^. Yap1 immuno-staining of clonal *Tead4*-KD embryos, detailed nuclear and unusually enriched cytoplasmic expression in outer-residing TE-inhibited cells of mid-16-cell stage embryos, contrasting with more typical nuclear enriched localisation in non-microinjected outer-cells (or control embryos); a similar pattern of Yap1 mis-localisation was also observed at the 32-cell (E3.6) stage, although outer cell staining was more prominently cytoplasmic by this stage. Using anti-sera previously reported^16^ to only recognise phosphorylated Yap1 (pYap1—Supplementary Fig. S8), we determined that the mis-localised Yap1 was non-phosphorylated, confirming the atypical cytoplasmic localisation was not because of the induction of active hippo-signalling. We speculate it may be associated with a lack of mechanism to retain Yap1 in the nucleus given its binding partner’s absence (*i.e.* Tead4), and thus reflects an equilibrium of the active nuclear import and passive export of non-phosphorylated Yap1. Curiously, we also observed nuclear localised pYap1 in outer mid-16-cell stage blastomeres both within and outside the Tead4-dsRNA microinjected clone (Supplementary Fig. S8a,b). pERM immuno-staining of 32-cell (E3.6) stage embryos revealed robust apical expression within outer-residing TE-inhibited clones, associated with rounded non-TE like morphology (Fig. 5) at frequencies consistent with subsequent apoptosis (see Supplementary Fig. S5 and Supplementary Tables ST6–ST8). Overall, we did not observe any apical polarisation deficits/or intracellular mis-localisations within TE-inhibited clones that would be consistent with the generation of the extra ICM founder cells (Fig. 3), nor did we observe such deficits when assaying other apical polarity (Prkcz and Pard6b), basolateral polarity (Scrib) or cell adhesion molecular markers (Cdh1—see Supplementary Fig. S9).

**Figure 6:**
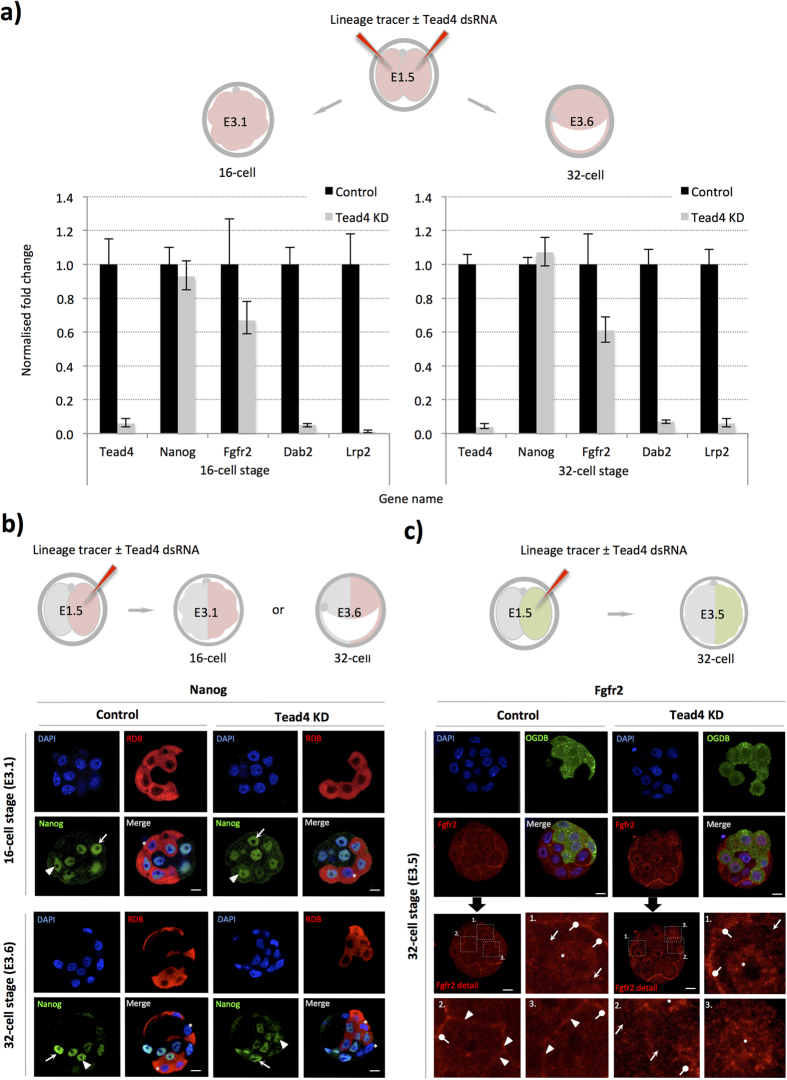
Global and clonal TE-inhibition; no enhanced *Nanog* expression prior to 32-cell stage but attenuated PrE-specific marker expression. (**a**) The experimental strategy to down-regulate *Tead4* and inhibit TE-differentiation throughout all cells of the embryo prior to Q-RTPCR analysis (upper). Normalised expression fold changes, resulting from *Tead4*-KD, of the stated transcripts at the mid-16-cell (E3.1) or 32-cell (E3.6) stages (lower panels). Individual gene mRNA levels were normalised against Rpl23 and/or H2afz transcript levels within control and experimental knockdown conditions prior to fold change calculation. Errors = s.e.m, n = at least 2 for biological and 3 for technical replicates (*n.b. Tead4* specific data is repeated from Fig. 1 as Q-RTPCR was performed from same cDNA preparations). (**b**) Confocal microscopy analysis of Nanog (green) expression after clonal *Tead4*-KD at the mid-16-cell (E3.1) and 32-cell (E3.6) stages. Arrows and arrow-heads denote exemplar Nanog expression in non-microinjected and microinjected clones, respectively. Asterisks highlight TE cells without Nanog expression reflecting previously characterised inter-cell heterogeneity. (**c**) Confocal microscopy analysis of Fgfr2 (red) expression after clonal *Tead4*-KD at the non-cavitated 32-cell (E3.5) blastocyst stage. Representative single z-plane confocal micrographs are shown (middle panels) with the lower 4 panels detailing magnified anti-Fgfr2 immuno-stained images, according to numbered regions of interest. Arrow-heads highlight plasma membrane associated Fgfr2 between neighbouring ICM cells (control embryos) and arrows approximate equivalent regions of other neighbouring ICM cells without anti-Fgfr2 signal (illustrating heterogeneous *Fgfr2* expression within control embryo ICMs). Similarly, arrows show ICM cell boundaries between cells of the microinjected clone devoid of Fgfr2 in *Tead4*-KD embryos. Asterisks and lollipop markers, in both control and *Tead4*-KD embryos, show nuclear Fgfr2 protein (especially in Tead4-KD embryos) or expression at the interface of TE and ICM cells, respectively. In both (**b**,**c**) progeny cells of microinjected clones are distinguishable by co-injected RDBs (red) or OGDBs (Oregon-green dextran beads—green). DNA was counterstained with DAPI (blue) and scale bars = 10 μm.

We next ascertained if TE-inhibited clone segregation towards EPI cell-fate was associated with precocious and/or increased expression of the EPI marker gene *Nanog*^37^. We first assayed *Nanog* derived transcript levels in 16- (E3.1) and 32-cell (E3.6) stage embryos derived from embryos microinjected in both blastomeres at the 2-cell (E1.5) stage with RDBs ± Tead4-dsRNA, to effect global TE-inhibition in all cells of the embryo (Fig. 6a). We found that Nanog mRNA levels remained unchanged at both stages. We therefore assayed Nanog protein expression, but utilising the clonal down-regulation model by microinjecting one blastomere at the 2-cell (E1.5) stage to permit side-by-side comparison of Nanog protein levels in non-microinjected and TE-inhibited microinjected clones (Fig. 6b). Similarly, we did not detect any difference in Nanog protein expression levels between the microinjected and non-microinjected clones in both control and *Tead4*-KD embryos or between the embryo groups, confirming TE-inhibition by *Tead4*-KD is not associated with enhanced or precocious *Nanog* expression.

We next hypothesised that the EPI bias of TE-inhibited ICM residing clones may be associated with attenuated PrE-related gene expression, and thus indicative of a potential obstacle for such cells to initiate or maintain PrE differentiation. Accordingly, we investigated the expression levels of PrE-related gene mRNAs following TE-inhibition (Fig. 6a). We found that, in contrast to *Nanog*, the normalised transcript levels for the PrE marker genes *Lrp2*^38^, and *Dab2*^39^ were down-regulated >95%. Moreover, we also detected a ~40% reduction in *Fgfr2* mRNA transcripts, a gene required to mediate PrE-promoting Fgf4-based signalling in the ICM^20^,^21^. We also observed the same trend of attenuated PrE-related gene mRNA expression in clonally TE-inhibited/*Tead4*-KD late blastocysts (E4.5), derived by microinjection of a single blastomere at the 2-cell (E1.5) stage (Supplementary Fig. S10). Given the centrally important role of Fgf4 signalling within maturing ICMs^20^,^21^, we decided to assay Fgfr2 protein expression after clonal TE-inhibition (Fig. 6c—note microinjected clones were marked with Oregon-green-conjugated dextran beads/OGDBs rather than RDBs). In microinjection control embryos, we observed Fgfr2 in regions demarking approximate boundaries between TE and ICM cells, characteristic of plasma membrane association (the microscopic resolution used prevented exact cellular origin to be determined). Similarly, we also detected Fgfr2 expression at regions between some fellow ICM cells but notably not all potential ICM-to-ICM cell contact regions. We interpret these data to indicate heterogeneous inter-cell, membrane associated, Fgfr2 protein expression in the ICMs of control embryos; an expression pattern consistent with a concurrent and similar heterogeneous and reciprocal inter-cell ‘salt and pepper’ expression pattern for EPI (Nanog) and early PrE (Gata6) markers, already long described^37^. Importantly, in *Tead4*-KD embryos we did not detect Fgfr2 protein at any ICM-to-ICM cell contact region between cells of the microinjected clone. Moreover, such inner-cells also exhibited prominent nuclear localised Fgfr2. Interestingly, nuclear localised Fgfr2 was seen in some, but not all, inner-cells of control embryos, with the suggestion that nuclear-enriched cells also lack plasma membrane associated Fgfr2, akin to TE-inhibited clones (Fig. 6c). It is possible that nuclear sequestration, be it in some ICM cells of control embryos or within the TE-inhibited ICM clones of *Tead4*-KD embryos, blunts their capacity to respond to Fgf4-based signals at the plasma membrane and thus differentiate towards PrE fate.

In summary, the reported attenuated PrE formation of TE-inhibited ICM clones is not associated with increased expression of the EPI marker *Nanog*, nor with reduced apical-basolateral polarity. Inhibition of TE cell-fate, does however correlate with reduced transcript levels for PrE marker genes and down-regulation of Fgfr2 protein from the plasma membrane of ICM cells.

## Discussion

We created variously sized cell clones within developing preimplantation mouse embryos that were unable to initiate TE-differentiation and assayed their ICM cell-fate potential to see if such clones would be predisposed towards EPI rather than PrE, in a similar manner to that described for ICM founders generated during the fourth cleavage^22^. In each of our experiments we found this to be true, as ICM cells derived from TE-inhibited clones preferentially contributed EPI versus PrE cells. These data indicate that level of TE-differentiation exposure ancestral cells of ICM progenitors are exposed to, is functionally relevant, although certainly not deterministic, in regard to guiding their ultimate fate. We conclude that our data are consistent with the integrated cell-fate model, whereby differing levels of exposure to TE-differentiative cues, provided by prolonged inhibition of hippo-signalling in outer-cells that yield ICM founders at the fifth cleavage division rather than those that do at the fourth, biases ICM cells to preferentially occupy either EPI or PrE.

A question arising from our clonal studies is: Are ICM progenitors generated as a result of the fourth or fifth cleavage, or more precisely from cells with differing exposures to TE-differentiation, molecularly equivalent? Others report, the first population of derived inner-cells are characterised by higher *Fgf4* and lower *Fgfr2* expression, whilst the second population exhibit relatively elevated *Fgfr2* expression^26^,^31^, consistent with our observations of reduced Fgfr2 mRNA levels after *Tead4*-KD (Fig. 6a); these data support the hypothesis that prolonged exposure to TE-differentiation, primes cells to yield ICM progeny biased towards PrE, by becoming more responsive to Fgf4^21^. Moreover, still others report that the *Fgfr2* gene itself, plus the *Lrp2* and *Dab2* genes, are occupied by promoter proximal Tead4 protein in TS cells^40^. We observed a lack of detectable Fgfr2 protein expression between adjacent ICM cells within the *Tead4*-KD clone (plus increased nuclear localisation), suggesting that TE inhibited clones are less able to differentiate to PrE because of reduced ability to receive Fgf4-based membrane signals. Interestingly, a similarly heterogeneous anti-Fgfr2 immuno-staining pattern has previously been reported in mouse blastocysts, although the significance of this observation was not discussed^41^. The reason for enhanced Fgfr2 nuclear localisation is unclear, although not unprecedented^42^,^43^ and is observed heterogeneously in control embryo ICM cells populations (Fig. 6c).

A recent genetic study demonstrated that restricted inner-cell expression of the pluripotency-related transcription factor *Sox2* is also regulated by hippo-signalling in a *Tead4*-dependant manner^44^. This report is consistent with the integrated cell-fate model as it highlights the importance of the relatively early and late removal of cells from TE-differentiative environments. The reported data also suggest that the EPI bias of TE-inhibited clones (Figs 3 and 4 and Supplementary Figs S2–S4, S6 and S7) maybe due to increased *Sox2* expression. A previous study investigating ICM lineage contribution of varying ratios of marked inner-cells, in chimeric 16-cell stage embryos, demonstrates that the initial number of ICM founders has profound effects for cell-fate. Chimeras generated with fewer (*e.g.* 3) inner-cells exhibited EPI bias within these ICM founders, reminiscent of previous reports of non-manipulated *in vitro* cultured time-lapse observed embryos^22^, in addition to PrE bias of unmarked cells internalised following the fifth cleavage. However, generating chimeras with more initial inner-cells (*e.g.* 4 or 5) ablated the biases^26^. The authors speculated elevated extra-cellular Fgf4 levels in chimeras with more ICM founders, causes cells that may have otherwise contributed to EPI, to differentiate to PrE; a hypothesis also supported by mathematical modelling of ICM cell-fate^45^. In our data (Figs 3 and 4 and Supplementary Figs S2–S4, S6 and S7), we observed the greatest percentage PrE contribution of non-TE-inhibited ICM-resident clones when the TE-inhibited/*Tead4*-KD clone in the ICM was largest, and *vice-versa*. We interpret these results as indirect evidence that larger TE-inhibited clones may generate greater levels of extra-cellular Fgf4 within the ICM than smaller clones. As such, our data support the relevance of the number of generated ICM cells, and not just their developmental origin, as being important in biasing/directing lineage segregation^26^,^45^. In all our experimental strategies, we observed PrE contribution of cells derived from TE-inhibited clones, indicating that removal from TE-differentiation does not ‘block’ PrE differentiation. It is possible that elevated Fgf4 levels in ICMs containing TE-inhibited clones are sufficient to act upon cells within the clone itself. Alternatively, stochastic mechanisms could also be involved, whereby inherent self-reinforcing fluctuations in gene expression would predispose TE-inhibited ICM cells towards a profile favourable for PrE differentiation. Indeed, stochastic mechanisms are promoted as a means by which ICM lineage segregation normally occurs^46^. We would argue that our data indicate significant biases in ICM cell-fate derivation based on cell history but do not dismiss the existence, input nor importance of stochastic mechanisms. Indeed, early mammalian development is highly regulative, indicating, by definition, the existence of multiple complementary and overlapping pathways that must work together to guide, rather than restrict, the appropriate cell-fate decisions required^47^.

Theoretically our results are explainable by a direct and unprecedented PrE promoting role for *Tead4* within the ICM, potentially acting via inactivation of the hippo-signalling pathway. However, existing ES cell evidence argue against this hypothesis. Firstly, as *Tead4*^*−/−*^ ES cell differentiation leads to the expression of multiple lineage marker genes, including the PrE markers *Gata6* and *Sox17*, the evidence suggests that *Tead4* is dispensable for PrE formation *per se* (agreeing with our clonal data)^9^. Moreover, when we performed double immuno-fluorescent staining of, control microinjected and *Tead4*-KD (microinjecting one blastomere at the 2-cell stage) or *in vitro* cultured control late blastocysts (E4.5) for Yap1 and Gata4 (Supplementary Fig. S11), we did not observe any nuclear co-localisation of the two factors that would have been indicative of activated hippo-signalling in specified PrE cells. Nor, did we observe any indication of nuclear Yap1 in any inner/ICM cells at earlier developmental points prior to the late blastocyst (E4.5) stage (Fig. 5 and Supplementary Fig. S8). We therefore consider it unlikely that Tead4 has a direct PrE promoting role within the ICM. Furthermore, given that it is even possible to derive *Tead4*^*−/−*^ ES cells, a direct requirement of *Tead4* to maintain the pluripotent EPI lineage is also highly improbable^9^. However, it is important to note that ES cells may not share the same degree of functional similarity with cells of the early embryo^48^. Overall, the collective evidence points against any direct cell-fate role of *Tead4* within the ICM. Consequently, our clonal *Tead4*-KD data are best explained by the inability of the ancestors of ICM progenitors to initiate/respond to TE-differentiative cues, rather than a lack of functionally relevant Tead4 protein within individual ICM cells.

Our observations of pERM and Yap1 immuno-staining (Supplementary Fig. S8) are completely consistent with an earlier report detailing the existence of apolar outer-cells with cytoplasmically localised Yap1 protein, indicative of active hippo-signalling^6^. However, we did observe unexpected nuclear pYap1 in outer-residing, mid-16-cell stage blastomeres (Supplementary Fig. S8a,b). This result is unintuitive, as phosphorylation of Yap1 is normally associated with its cytoplasmic sequestration in response to active hippo-signalling, and neither the reason nor its functional significance are clear. Possibly, the phospho-serine-127 epitope recognised by the antibody is not a sufficient/determining factor governing nuclear exclusion at this stage; for instance Yap1 has five known hippo-signalling directed (by Lats) phosphorylatable serine containing consensus motifs^49^.

We conclude our data are compatible with the integrated cell-fate model of early lineage segregation, whereby TE, EPI and PrE fates all begin their segregation at the fourth cleavage division and are guided by relative differences in the exposure of the founding cells to TE-differentiative cues and the timely activation (or liberation from suppression) of the hippo-signalling pathway. However, and in keeping with the remarkable regulative capacity of the early embryo, such mechanisms act to guide rather than dictate cell-fate and can be modulated by factors such as generated inner-cell number or the induction of self-reinforcing stochastic gene expression.

## Methods

All described experimental procedures on mice and mouse embryos were approved, ratified and licensed by the local ethics committee of the Biology Centre (in České Budějovice) of the Czech Academy of Sciences and by the responsible committee of the Czech Academy of Sciences on the national level, in accordance with Czech and European Union law. Accordingly, the described experiments were implemented in full accordance with the approved guidelines provided by the two above stated committees.

### Embryo culture

Embryos were recovered into M2 medium containing 4mg/ml BSA (M2 + BSA) from oviducts of 10 week F1 hybrid (C57Bl6 × CBA/W) female mice (mated with F1 males) after superovulation with 7.5 IU PMSG (Sigma) and 7.5 IU hCG (Sigma; administered 48 hours later). 2- and 4-cell stage embryos were recovered from dissected oviducts 44 and 53 hours post-hCG. Unless microinjected immediately, recovered embryos were washed through KSOM drops (EmbryoMax, Millipore) and cultured under mineral oil in 5% CO_2_ at 37 °C (15 embryos/20 μl drop).

### Tead4-dsRNA generation and microinjection

A *Tead4* specific PCR primer pair, incorporating T_7_-derived RNApol promoters (sense; TAATACGACTCACTATAGGGTGTTGGAGTTCTCGGCTTTC, antisense; TAATACGACTCACTATAGGGTCGGTAGATGTGGTGCTGAG, T_7_-promoter underlined) was used to derive *in vitro* transcription template (from HMI ES cell cDNA) for Tead4 mRNA specific long double-stranded RNA synthesis (Tead4-dsRNA; MEGAscript T_7_; Ambion), according to manufacturers protocol. Tead4-dsRNA uniquely and specifically targets exons 7 to 11 of transcript ENST00000006311. GFP mRNA specific double stranded RNA (GFP-dsRNA) was similarly produced using specific T7-promoter linked primers (sense; TAATACGACTCACTATAGGGAGAGTACAAATTTTCTGTCAGTGGAGAGG, antisense; TAATACGACTCACTATAGGGAGATGTATAGTTCATCCATGCCATGTGTA) using pRN3P:EGFP^22^ plasmid DNA as initial template. The integrity of both Tead4-dsRNA and GFP-dsRNA was confirmed on non-denaturing agarose gels.

Single blastomere microinjections were performed as previously described^50^ on 2- or 4-cell stage embryos in suspended M2 + BSA media drops using IX71 inverted-microscope (Olympus), micromanipulators (Leica) and FemtoJet microinjection system (Eppendorf). Tead4-dsRNA (100 ng/μl) was co-microinjected with rhodamine-/Oregon-green-conjugated dextran beads (RDBs/OGDBs; 2 μg/μl and 1 μg/μl), with controls comprising RDBs/OGDBs microinjection alone or RDBs plus GFP-dsRNA (100 ng/μl). Non-microinjected embryos (1–3 per experiment) served as sentinels for appropriate *in vitro* development.

### Embryo chimeras

Fluorescent microinjected (both blastomeres at 2-cell/E1.5 stage) donor non-compacted 8-cell stage (E2.5), *zona*-less (acid tyrodes treatment), embryos were transferred into Ca^2+^/Mg^2+^ free M2 media and disaggregated into single blastomeres. Per chimera, single fluorescently-labelled control or *Tead4*-KD blastomeres were placed in contact with non-compacted *zona*-less 8-cell stage (E2.5) embryos in PHA (phytohaemagglutin—300 μg/mL; Sigma) containing M2 media and incubated for 10 minutes (37 °C and 5% CO_2_). Following confirmation of aggregation, chimeras were returned to conventional KSOM culture until the late blastocyst (E4.5) stage. All manipulations were performed in pre-warmed (37 °C) media under mineral oil on heated stereo-dissecting microscope stages.

### Immuno-fluorescent staining

Embryos were fixed in 4% PFA (20 minutes, 37 °C) and prepared for confocal-based immuno-fluorescence microscopy as follows (at room temperature in 150 μl, unless stated); i) three 5 minute PBS washes, ii) 20 minutes in 0.5% (in PBS) Triton X-100 (Fgfr2 immuno-staining 0.1% Triton X-100), iii) three 10 minute PBS-Tween 20 (0.15%; PBS-T) washes, iv) one 10 minute NH_4_Cl (50 mM in PBS) wash, v) one 4 hour 3% BSA (in PBS-T; BSA-PBS-T) blocking-step (4 °C), vi) overnight primary antibody (diluted in BSA-PBS-T) incubation (4 °C), vii) three 10 minute PBS-T washes, viii) 4 hour BSA-PBS-T blocking-step (4 °C), ix) 1 hour fluorescently-labelled secondary antibody incubation (diluted in BSA-PBS-T, 4 °C), x) three 10 minute PBS-T washes, xi) 30 minutes PBS wash, and xii) mounting (Vectashield plus DAPI, Vector Labs) on glass-bottomed, poly-L-lysine coated culture dishes. For phospho-Yap1 (pYap1), some embryos were pre-treated with 1000 units λ-phosphatase (sc-200312, Santa Cruz Biotech) according to manufacturers protocol. Primary and secondary antibody details and dilutions are given in Supplementary Table ST14. Note, that for embryos double immuno-stained for Nanog and Gata4, the rat monoclonal version of the anti-Nanog antibody was used. Embryos were imaged on IX81 upright or Fluoview Fv10i confocal microscopes (Olympus).

### Image analysis/cell counting

Individual E4.5 stage blastocyst cell contributions within and outwith the microinjected clone, or within inner or outer-cell populations, or within cell lineage marker protein positive or negative cells (after specific immuno-staining), plus the incidence of cells with fragmented/apoptotic nuclei, were determined in both experimental and control embryos by inspection of confocal z-sections using Fluoview ver.1.7.a (Olympus) and Imaris 6.2.1 (BitPlane) software. The above contribution criteria in *in vitro* culture control embryo groups (starting from 2-cell/E1.5 stage) was first established (Supplementary Fig. S12 and Supplementary Table ST13) to provide a baseline for the appropriate development of individual control and experimental embryos to be screened, removing those with generally poor development due to manipulation (particularly in chimeras). Accordingly, embryos with fewer than 64, or less than four Gata4 positive cells (minimum we observed in the reference data) were excluded from the analysis.

### Statistical analysis

The mean of cells within defined embryonic populations and the standard error of means (mean ± s.e.m.) were calculated and statistical significance determined using 2-tailed Student’s t-tests.

### Quantitative RTPCR (Q-RTPCR)

Total RNA was prepared from ~30 cultured 16- (E3.1) or 32-cell stage (E3.6) *Tead4*-KD or control embryos previously microinjected at the 2-cell stages (E1.5, in either one or both blastomeres) as instructed (Arcturus Biosciences; ‘PicoPure RNA isolation’). Eluted RNA (10 μl) was DNaseI treated (Ambion; ‘DNA-*free*’ kit) and used to derive cDNA (30 μl) using oligodT priming (Invitrogen; ‘SuperscriptIII Reverse Transcriptase’). 0.5 μl of diluted cDNA (1:3, nuclease-free water) was used as template in 10 μl real-time PCR reactions (Qiagen: ‘SYBR Green PCR kit’) to assay specific transcripts (BioRad, ‘CFX96 Real-Time System’)—see Supplementary Table ST15 for oligonucleotide primer sequences (final reaction conc. 400 nM). Transcript levels were internally normalised against Rpl23 (60S ribosomal subunit component) and/or H2afz levels, and fold changes (plus s.e.m.) derived using the ΔΔCt method^51^. A minimum of 2 biological replicates of at least three technical replicates were employed.

## Additional Information

**How to cite this article**: Mihajlović, A. I. *et al.* The first two cell-fate decisions of preimplantation mouse embryo development are not functionally independent. *Sci. Rep.* **5**, 15034; doi: 10.1038/srep15034 (2015).

## Supplementary Material

Supplementary Information
