# Supplementary material for: The first two cell-fate decisions of preimplantation mouse embryo development are not functionally independent
Source: Sci Rep. 2015 Oct 13;5:15034. doi: 10.1038/srep15034 (PMC4602213; doi:10.1038/srep15034)
Supplement: Supplementary Information [file srep15034-s1.pdf]

The first two cell-fate decisions of preimplantation mouse embryo development are not functionally independent

Aleksandar I. Mihajlović, Vasanth Thamodaran and Alexander W. Bruce

SUPPLEMENTARY FIGURES & TABLES, PLUS LEGENDS

INC: x12 Supplementary figures

X15 Supplementary tables

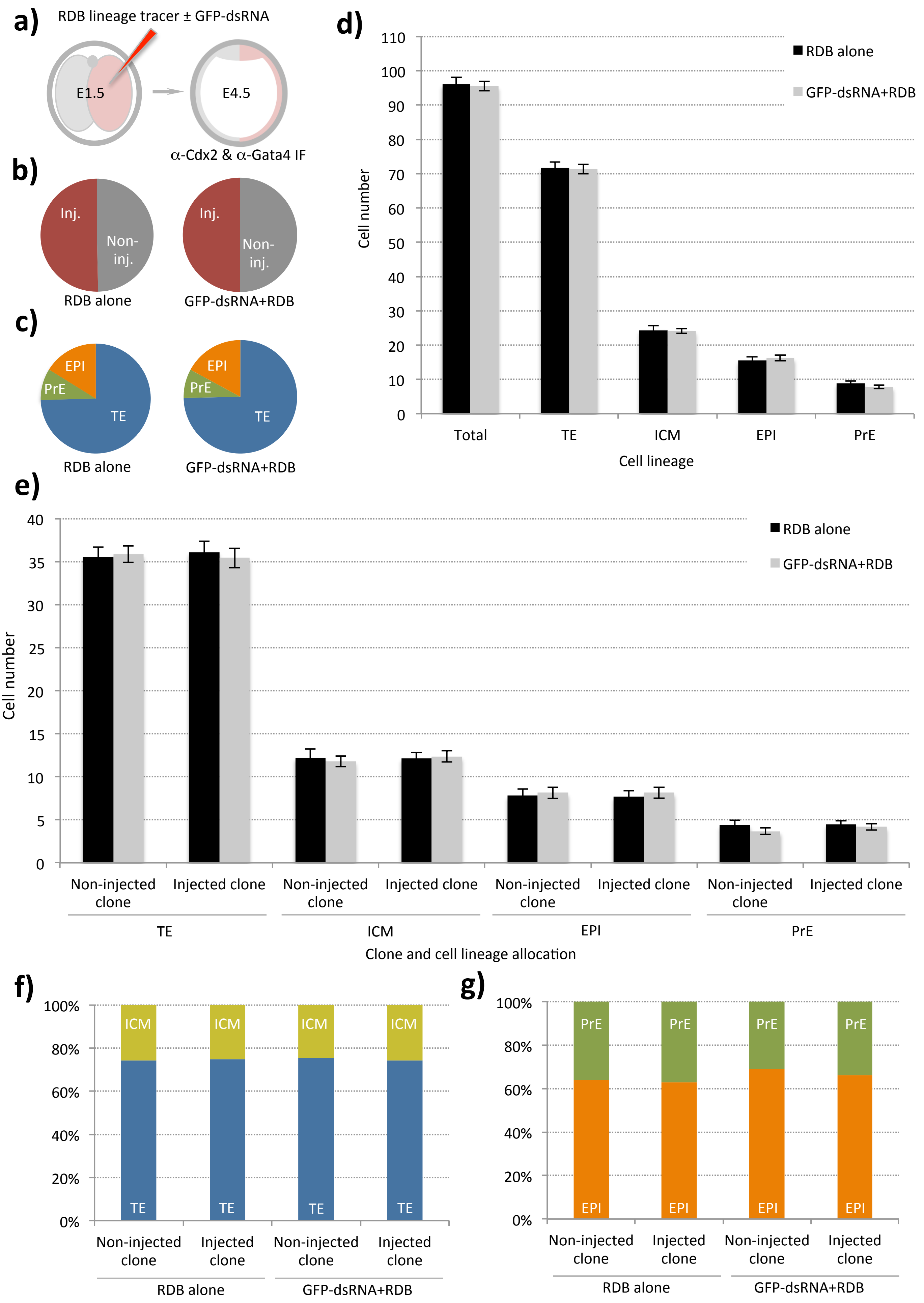

Supplementary figure S1: Clonal inhibition of TE-differentiation preferentially biases cells to EPI rather than PrE fates (Cdx2 and Sox17 lineage marker detection).

Supplementary figure S1: There is no statistically significant difference between blastocyst lineage derivation between two groups of clonal control embryos microinjected in one cell at the 2-cell stage with either RDBs alone or RDBs + GFP-dsRNA. a) Schematic of experimental strategy to clonally mark one-half of control embryo cells with either RDBs or RDBs + GFP-dsRNA and assess cell lineage allocation in late blastocysts (E4.5) via Cdx2 (TE marker) and Gata4 (early PrE marker) immuno-fluorescence detection (*n.b.* inner-cells devoid of either lineage marker were classified as EPI). **b)** Average total cell percentage contribution of microinjected and non-microinjected cell clones in either group of control microinjected embryos (*i.e.* RDB alone or GFP-dsRNA+RDB). **c)** Relative averaged percentage contribution of total cell number to the late blastocyst lineages (TE, PrE and EPI) in either clonal control group of microinjected embryos. **d)** Averaged total cell number for each late blastocyst lineage (ICM = EPI + PrE) in each group of clonal control embryos. **e)** Average number of cells from either non-microinjected or microinjected cell clones contributing to late blastocyst lineages, in each group of clonal control embryos. In e) and f), error bars represent s.e.m; */ ** and ‡/ ‡‡ denote statistically significant differences between equivalent cell clones of each group of clonal control embryos, or between cell clones within each control groups itself, respectively (confidence intervals of $p<0.05$ and $p<0.005$, 2-tailed student t-tests); however note the lack of statistically significant difference between the two groups of clonal control embryos, thus highlighting validity of using the RDB alone control group in relation to the experiments described in the main text of this report. **f)** Averaged percentage contribution of non-microinjected and microinjected cell clones, of each group of clonal control embryos, to TE or ICM of late blastocysts. **g)** Averaged percentage contribution of non-microinjected and microinjected ICM cell clones, of each group of clonal control embryos, to PrE or EPI lineages. Overall, in the RDB alone clonal control group of embryos n = 30 and in the GFP-dsRNA+RBD clonal control group n = 37 (see supplementary tables ST1 and ST2 for individual embryo cell allocation and apoptosis data, respectively).

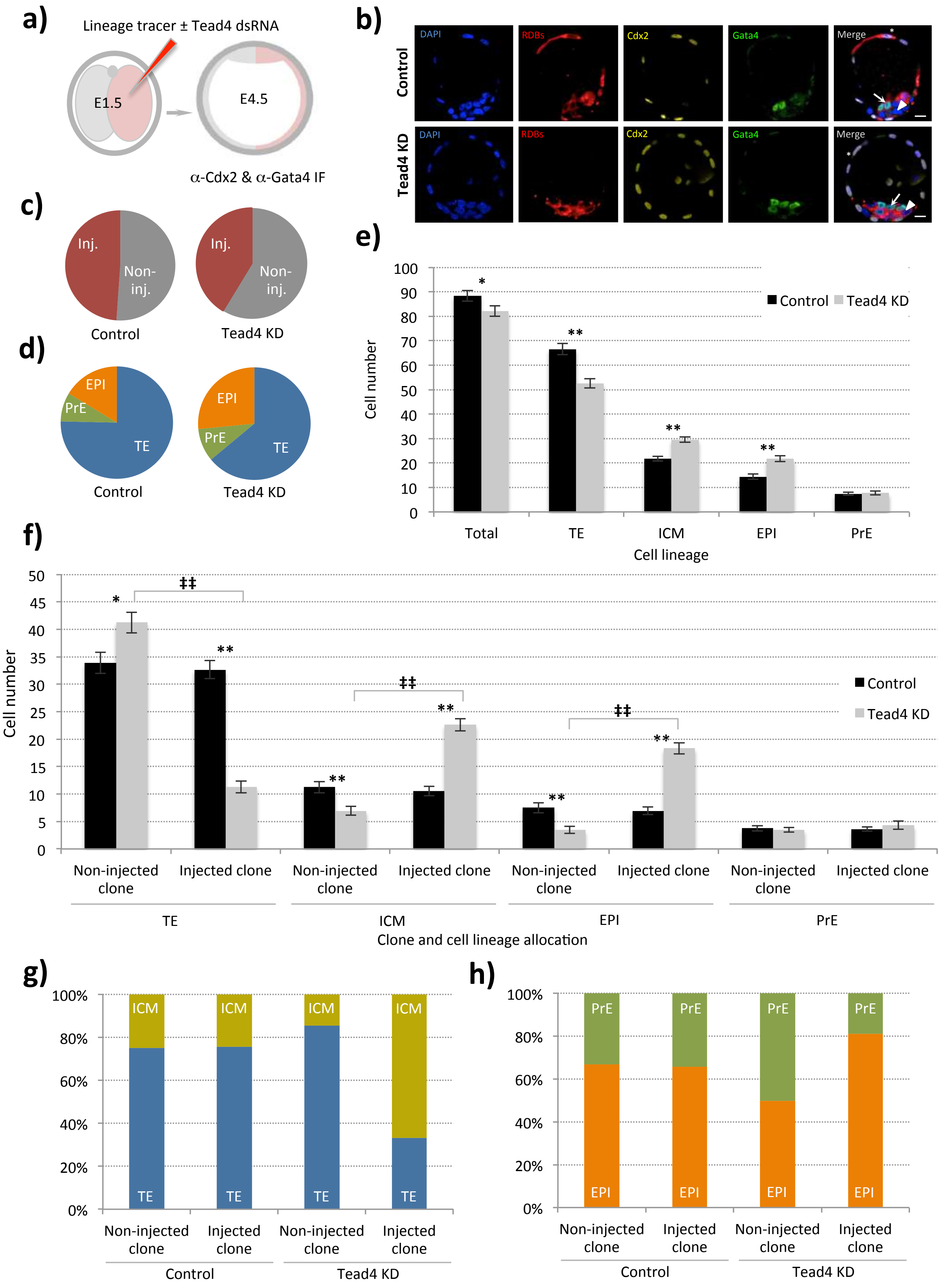

Supplementary figure S2: Clonal inhibition of TE-differentiation preferentially biases cells to EPI rather than PrE fates (Cdx2 and Gata4 lineage marker detection).

Supplementary figure S2: Clonal inhibition of TE-differentiation preferentially biases cells to EPI rather than PrE fates (Cdx2 and Gata4 lineage marker detection). a) Experimental strategy to effect clonal *Tead4*-KD and TE-inhibition in one-half of the embryo and assess cell lineage in late blastocysts (E4.5) via Cdx2 (TE marker) and Gata4 (late PrE marker) immuno-fluorescence detection (*n.b.* inner-cells devoid of either lineage marker were classified as EPI). **b)** Representative single z-plane confocal micrographs of microinjection control and clonal *Tead4*-KD late blastocyst (E4.5) embryos immuno-stained for Cdx2 (pseudo-coloured yellow) and Gata4 (green) protein expression. Progeny of the microinjected cell are distinguishable by co-injected RDB fluorescence (red). DNA is counterstained with DAPI (blue). Merged image asterisks represent exemplar cells classified in our analyses as TE, arrows as PrE cells and arrow-heads as EPI. Scale bars = 10μm. **c)** Average total cell percentage contribution of microinjected and non-microinjected cell clones in control and clonal *Tead4*-KD embryos. **d)** Relative averaged percentage contribution of total cell number to the late blastocyst lineages (TE, PrE and EPI) in control and clonal *Tead4*-KD embryos. **e)** Averaged total cell number for each late blastocyst lineage (ICM = EPI + PrE) in clonal *Tead4*-KD and control embryos. **f)** Average number of cells from either non-microinjected or microinjected cell clones contributing to late blastocyst lineages, in control and clonal *Tead4*-KD embryos. In e) and f), error bars represent s.e.m; */ ** and ‡/ ‡‡ denote statistically significant differences between equivalent cell clones of control and clonal *Tead4*-KD embryos, or between cell clones within control and clonal *Tead4*-KD embryo groups, respectively ($p<0.05$ and $p<0.005$, 2-tailed student t-tests). **g)** Averaged percentage contribution of non-microinjected and microinjected cell clones, of control and clonal *Tead4*-KD embryos, to TE or ICM of late blastocysts. **h)** As in g) but describing PrE and EPI lineage contribution in ICM. Overall, control embryos n = 24 and clonal *Tead4*-KD embryos n = 24 (Supplementary tables ST3 and ST6 individual embryo cell allocation and apoptosis data, respectively).

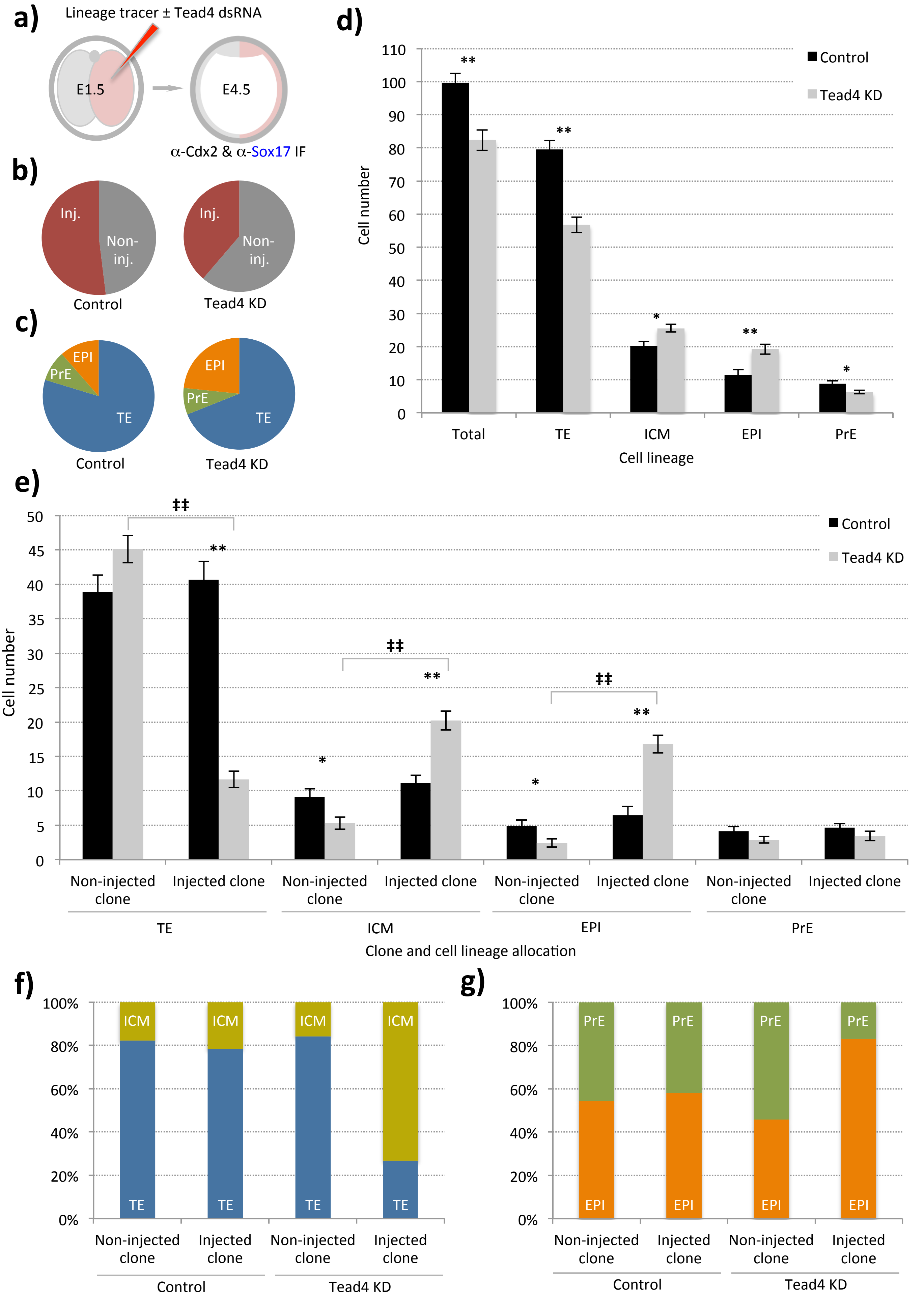

Supplementary figure S3: Clonal inhibition of TE-differentiation preferentially biases cells to EPI rather than PrE fates (Cdx2 and Sox17 lineage marker detection).

Supplementary figure S3: Clonal inhibition of TE-differentiation preferentially biases cells to EPI rather than PrE fates (Cdx2 and Sox17 lineage marker detection). a) Schematic of experimental strategy to effect clonal *Tead4*-KD and TE-inhibition in one-half of the embryo and assess cell lineage in late blastocysts (E4.5) via Cdx2 (TE marker) and Sox17 (early PrE marker) immuno-fluorescence detection (*n.b.* inner-cells devoid of either lineage marker were classified as EPI). **b)** Average total cell percentage contribution of microinjected and non-microinjected cell clones in control and clonal *Tead4*-KD embryos. **c)** Relative averaged percentage contribution of total cell number to the late blastocyst lineages (TE, PrE and EPI) in control and clonal *Tead4*-KD embryos. **d)** Averaged total cell number for each late blastocyst lineage (ICM = EPI + PrE) in clonal *Tead4*-KD and control embryos. **e)** Average number of cells from either non-microinjected or microinjected cell clones contributing to late blastocyst lineages, in control and clonal *Tead4*-KD embryos. In e) and f), error bars represent s.e.m; */ ** and ‡/ ‡‡ denote statistically significant differences between equivalent cell clones of control and clonal *Tead4*-KD embryos, or between cell clones within control and clonal *Tead4*-KD embryo groups, respectively (confidence intervals of $p<0.05$ and $p<0.005$, 2-tailed student t-tests). **f)** Averaged percentage contribution of non-microinjected and microinjected cell clones, of control and clonal *Tead4*-KD embryos, to TE or ICM of late blastocysts. **g)** Averaged percentage contribution of non-microinjected and microinjected ICM cell clones, in control and clonal *Tead4*-KD embryos, to PrE or EPI lineages. Overall, control embryos n = 13 and clonal *Tead4*-KD embryos n = 9 (see supplementary tables ST4 and ST7, individual embryo cell allocation and apoptosis data, respectively).

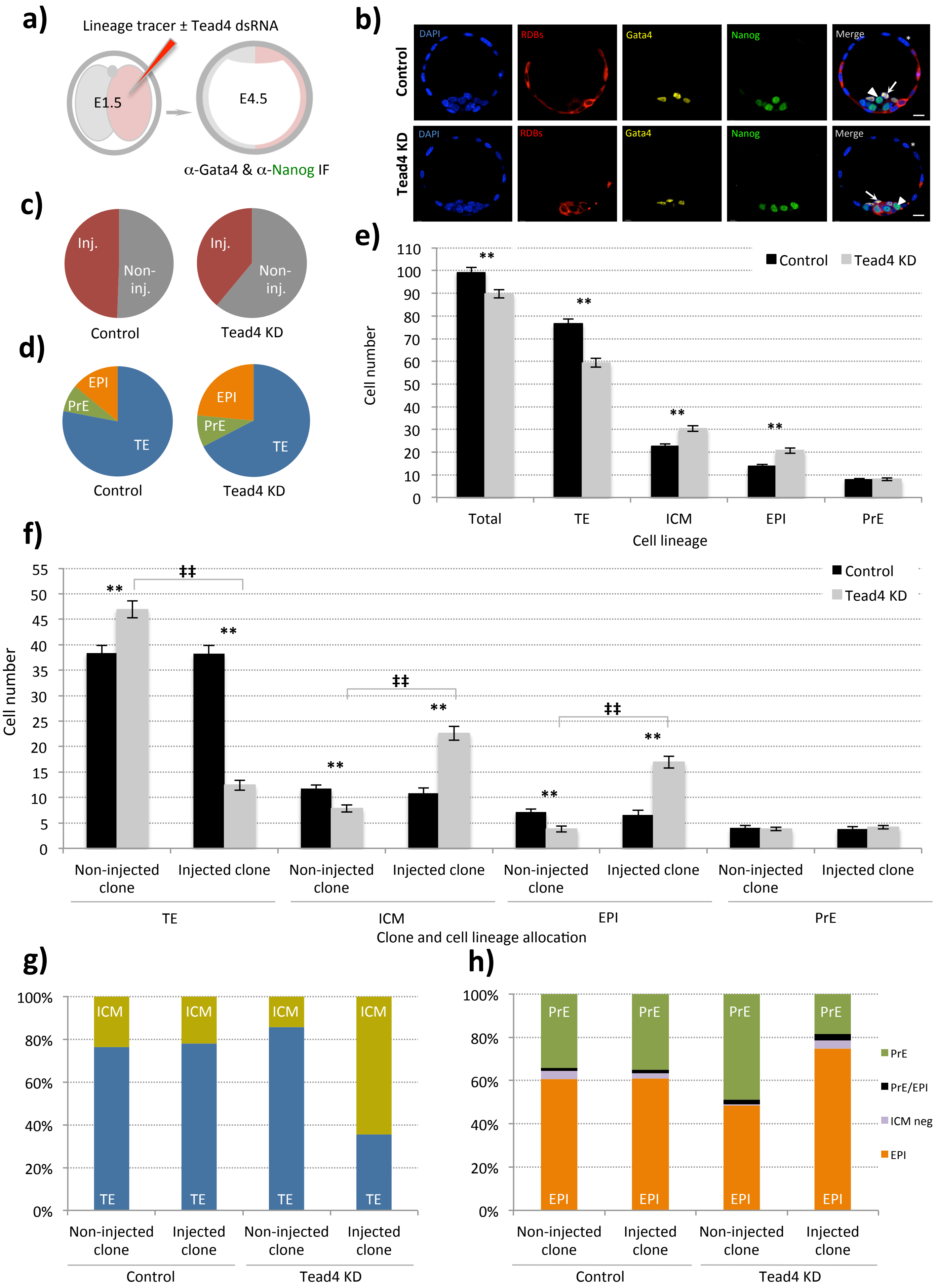

Supplementary figure S4: Clonal inhibition of TE-differentiation preferentially biases cells to EPI rather than PrE fates (Gata4 and Nanog lineage marker detection).

Supplementary figure S4: Clonal inhibition of TE-differentiation preferentially biases cells to EPI rather than PrE fates (Gata4 and Nanog lineage marker detection). a) Experimental strategy to effect clonal *Tead4*-KD and TE-inhibition in one-half of the embryo and assess cell lineage in late blastocysts (E4.5) via Gata4 (late PrE marker) and Nanog (EPI marker) immuno-fluorescence detection (*n.b.* outer-cells were classified as TE, inner cells that were either double negative or double positive for Gata4 and Nanog were also recorded). **b)** Representative single z-plane confocal micrographs of microinjection control and clonal *Tead4*-KD late blastocyst (E4.5) embryos immuno-stained for Gata4 (pseudo-coloured yellow) and Nanog (green) protein expression. Progeny of the microinjected cell are distinguishable by co-injected RDB fluorescence (red). DNA is counterstained with DAPI (blue). Merged image asterisks represent exemplar cells classified in our analyses as TE, arrows as PrE cells and arrow-heads as EPI. Scale bars = 10μm. **c)** Average total cell percentage contribution of microinjected and non-microinjected cell clones in microinjection control and clonal *Tead4*-KD embryos. **d)** Relative averaged percentage contribution of total cell number to definitive late blastocyst lineages (TE, PrE and EPI) in control and clonal *Tead4*-KD embryos. **e)** Averaged total cell number for each definitive late blastocyst lineage (ICM = EPI + PrE) in clonal *Tead4*-KD and control embryos. **f)** Average number of cells from either non-microinjected or microinjected cell clones contributing to the definitive late blastocyst lineages, in control and clonal *Tead4*-KD embryos. In e) and f), error bars represent s.e.m; */ ** and ‡/ ‡‡ denote statistically significant differences between equivalent cell clones of control and clonal *Tead4*-KD embryos, or between cell clones within control and clonal *Tead4*-KD embryo groups, respectively ($p<0.05$ and $p<0.005$, 2-tailed student t-tests). **g)** Averaged percentage contribution of non-microinjected and microinjected cell clones, of control and clonal *Tead4*-KD embryos, to TE or ICM of late blastocysts. **h)** As in g) but describing PrE and EPI lineage contribution in ICM, in addition to the relatively infrequent incidence of cells positively immuno-staining for both ICM lineage markers (PrE/EPI – black) or non at all (ICM neg – violet). Overall, control embryos n = 25 and clonal *Tead4*-KD embryos n = 23 (see supplementary tables ST5 and ST8, individual embryo cell allocation and apoptosis data, respectively).

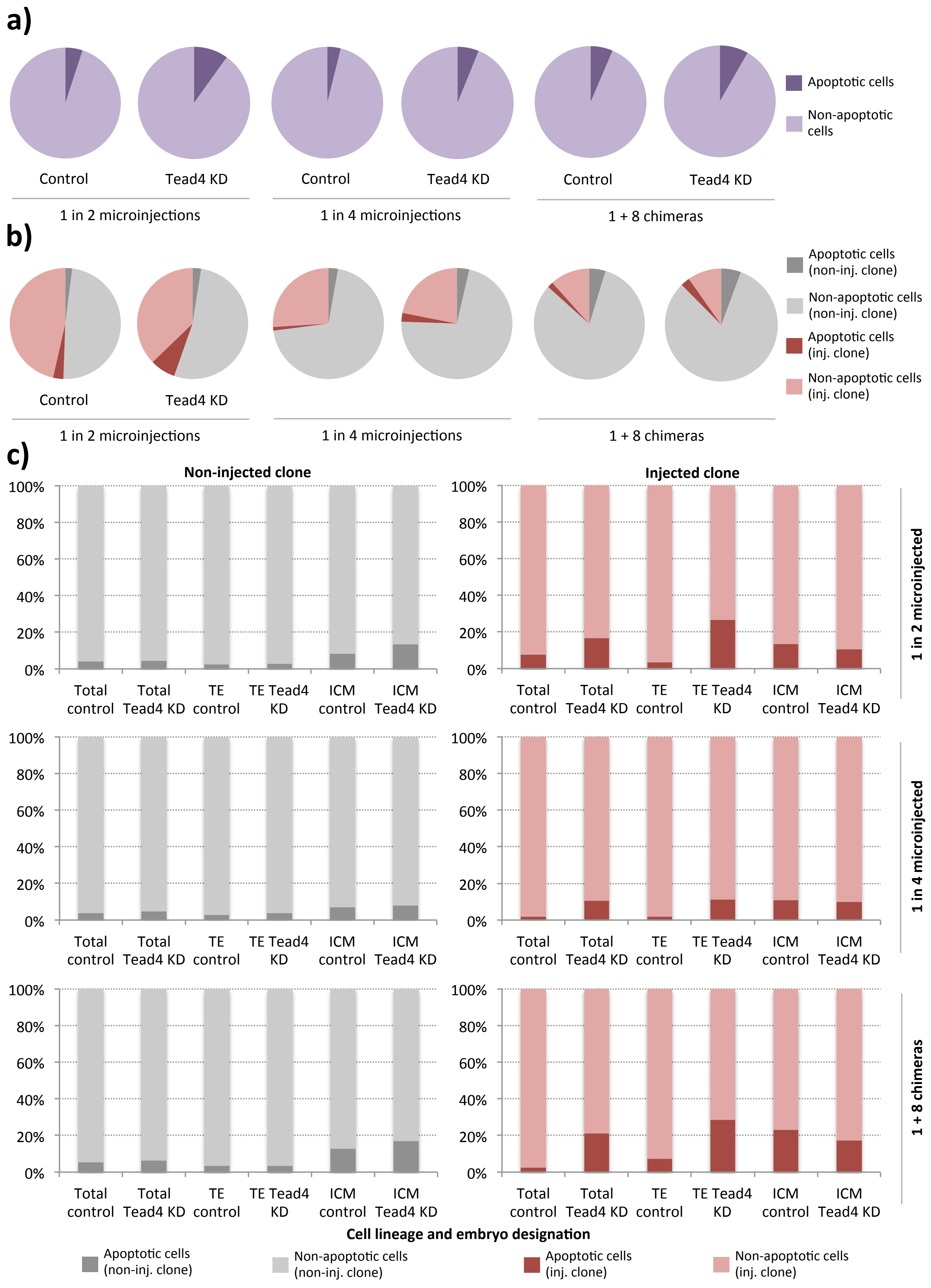

Supplementary figure S5: Average percentage rates of apoptosis in control and Tead4-specific dsRNA microinjected and chimeric embryos (Cdx2 and Gata4 lineage marker detection).

Supplementary figure S5: Average percentage rates of apoptosis in control and Tead4-dsRNA microinjected and chimeric embryos. a) Pie charts detailing average rates of total apoptosis observed (by the presence of fragmented inter-phase nuclei) in either control or Tead4-dsRNA microinjected embryos, cultured to the late blastocyst (E4.5) stage (whereby one cell at the 2- or 4-cell stages, referred to as '1 in 2' or '1 in 4 microinjections' respectively, was microinjected – data refers to embryos described in figure 3 and supplementary figures S2 & S6)**.** Pie charts describing the observed total apoptosis rates in chimeric embryos composed from unperturbed 8-cell stage embryos aggregated with either one control or one *Tead4* KD 8-cell stage blastomere (referred to as '1 + 8 chimeras' – data refers to embryos described in figure 4 and supplementary figure S7) and then similarly cultured to the late blastocyst, are also given. **b)** Pie charts describing the data given in a) but detailing the average rates of apoptosis in the non-microinjected and microinjected cell clones (unmarked and RDB-marked clones in chimeric embryos) of the cultured late blastocysts (cell clones are distinguishable by the presence or absence of injected RDB lineage tracer). **c)** Percentage bar charts expanding the data given in a) and b) but detailing the average incidence of apoptosis within each of the late blastocyst cell lineages of both control and *Tead4* knockdown embryos, in each of the three experimental paradigms ('1 in 2' and '1 in 4' microinjections, plus '1 + 8' chimeras). For individual embryo apoptosis data, refer to supplementary tables ST6, ST10 and ST12. All data were obtained from embryo groups immuno-stained for Cdx2 and Gata4.

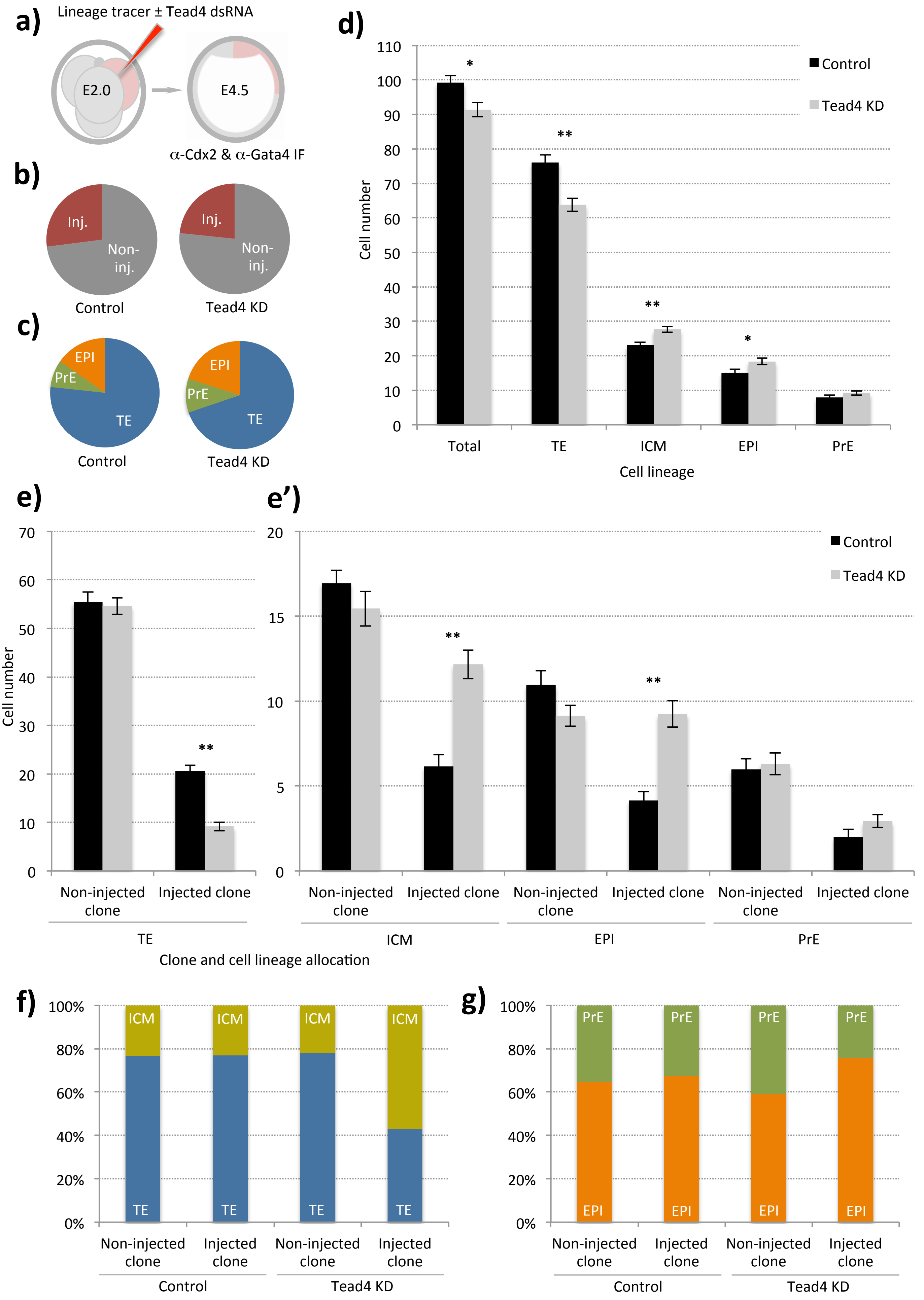

Supplementary figure S6: TE-differentiation inhibition within smaller cell clones (a quarter of the embryo) also biases cells to EPI rather than PrE fates.

Supplementary figure S6: TE-differentiation inhibition within smaller cell clones (a quarter of the embryo) also biases cells to EPI rather than PrE fates. a) Schematic of experimental strategy to effect clonal *Tead4*-KD and TE-inhibition in one-quarter of the embryo and assess cell lineage in late blastocysts (E4.5) via Cdx2 (TE marker) and Gata4 (late PrE marker) immuno-fluorescence detection. **b)** Average total cell percentage contribution of microinjected and non-microinjected cell clones in control and *Tead4*-KD embryos. **c)** Relative percentage contribution of total cell number to the late blastocyst lineages (TE, PrE and EPI) in control and *Tead4*-KD embryos. **d)** Averaged total cell number for each late blastocyst lineage (ICM = EPI + PrE) in *Tead4*-KD and control embryos. **e)** Average number of cells from either non-microinjected or microinjected cell clones in TE lineage, in control and *Tead4*-KD embryos. **e')** As in e) but describing contribution to the other late blastocyst lineages. In d), e) and e') error bars represent s.e.m; */ ** denote statistically significant differences between equivalent cell clones of control and *Tead4*-KD embryos (confidence intervals of $p<0.05$ and $p<0.005$, 2-tailed student t-tests). **f)** Percentage contribution of non-microinjected and microinjected cell clones, of control and *Tead4*-KD embryos, to TE or ICM of late blastocysts. **g)** Percentage contribution of non-microinjected and microinjected ICM cell clones, in control and *Tead4*-KD embryos, to PrE or EPI lineages. Overall, control embryos n = 19 and *Tead4*-KD embryos n = 29 (see supplementary tables ST9 and ST10 individual embryo cell allocation and apoptosis data, respectively).

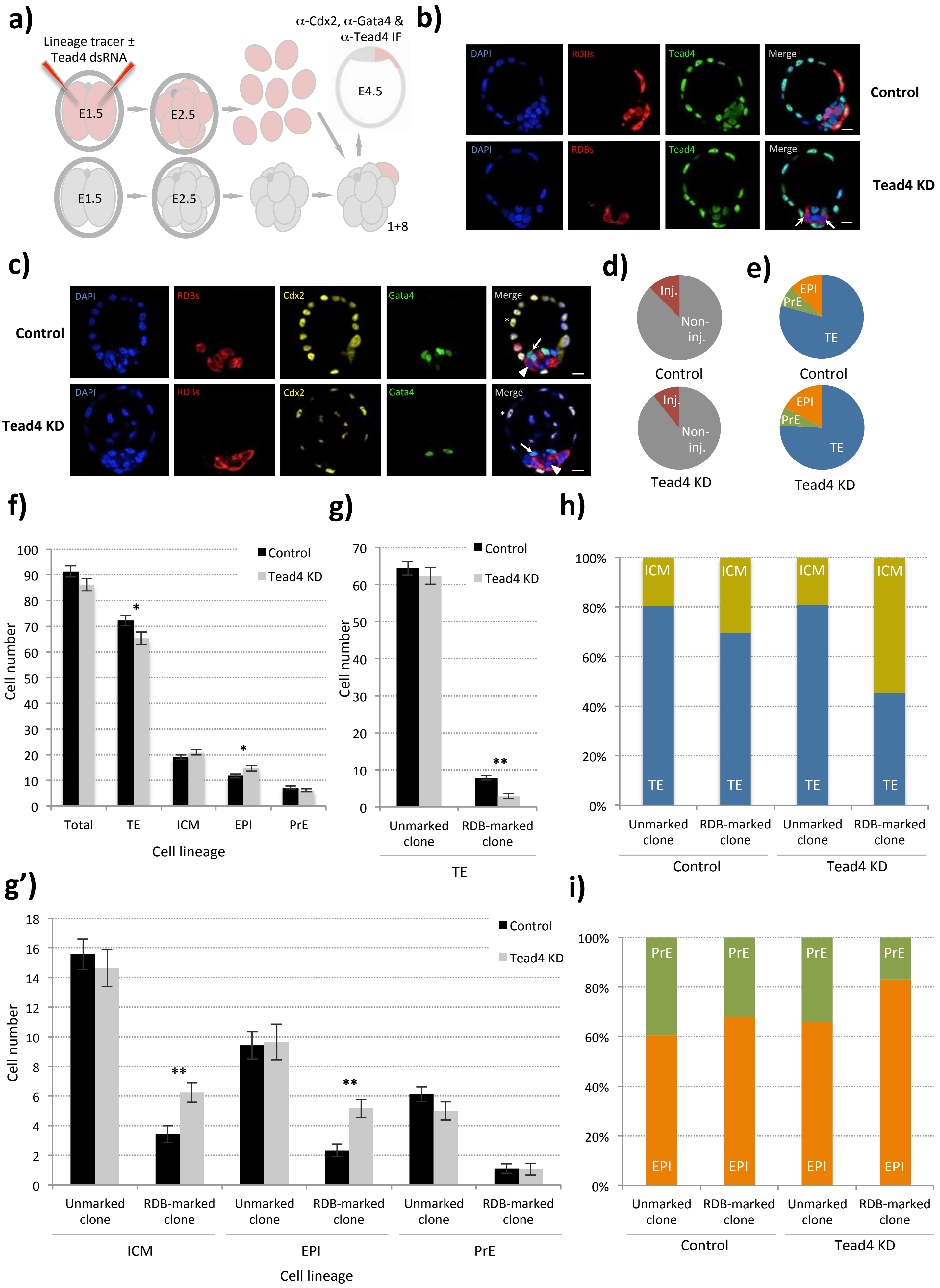

Supplementary figure S7: Clonal inhibition of TE-differentiation in 1+8 chimeric embryos preferentially biases cells to EPI rather than PrE fates (Cdx2 and Gata4 lineage marker detection).

Supplementary figure S7: TE-inhibition within small chimeric ICM clones also biases against ultimate PrE cell-fate. a) Experimental strategy to generate embryo chimeras containing TE-inhibited cells equivalent to one ninth of the embryo and to asses late blastocysts (E4.5) lineages (as in Fig. 3). **b)** Representative single z-plane confocal micrographs of control and *Tead4*-KD clone containing chimeras immuno-stained for Tead4 (green) expression. In merged image arrows denote cells not expressing detectable levels of Tead4 derived from original *Tead4*-KD donor blastomere **c)** Further, representative single z-plane confocal micrographs of late blastocyst (E4.5) chimeras immuno-stained for Cdx2 (pseudo-coloured yellow) and Gata4 (green). Merged image asterisks represent exemplar cells classified in our analyses as belonging to the TE, arrows PrE and arrow-heads EPI. In b) and c), cells deriving from donor blastomeres, themselves originally derived from control or Tead4-dsRNA microinjected 2-cell (E1.5) stage embryos, within chimeras are distinguishable by co-injected RDB (red). DNA is counterstained (DAPI, blue). Scale bars = 10μm. **d)** Average total cell percentage contribution of marked and non-marked cell clones. **e)** Relative average percentage contribution of total cell number to late blastocyst lineages in control- and *Tead4*-KD-chimeras. **f)** Averaged total cell number for each late blastocyst lineage (ICM = EPI + PrE) in control- and *Tead4*-KD-chimeras. **g)** Average number of cells from either RDB-marked or non-marked cell clones in TE lineage, in control and *Tead4*-KD-chimeras. **g')** As in g) but describing contribution to the other late blastocyst lineages. In f), g) and g') error bars represent s.e.m; */ ** denote statistically significant differences between equivalent cell clones of control- and *Tead4*-KD-chimeras ($p<0.05$ and $p<0.005$, 2-tailed student t-tests). **h)** Percentage contribution of RDB-marked and unmarked cell clones, in control- and *Tead4*-KD-embryos, to TE or ICM of late blastocysts. **i)** As in h) but describing PrE and EPI lineage contribution in ICM. Overall, control-chimeras n = 30 and *Tead4*-KD-chimeras n = 17 (Supplementary tables ST11 and ST12 individual embryo cell allocation and apoptosis data, respectively).

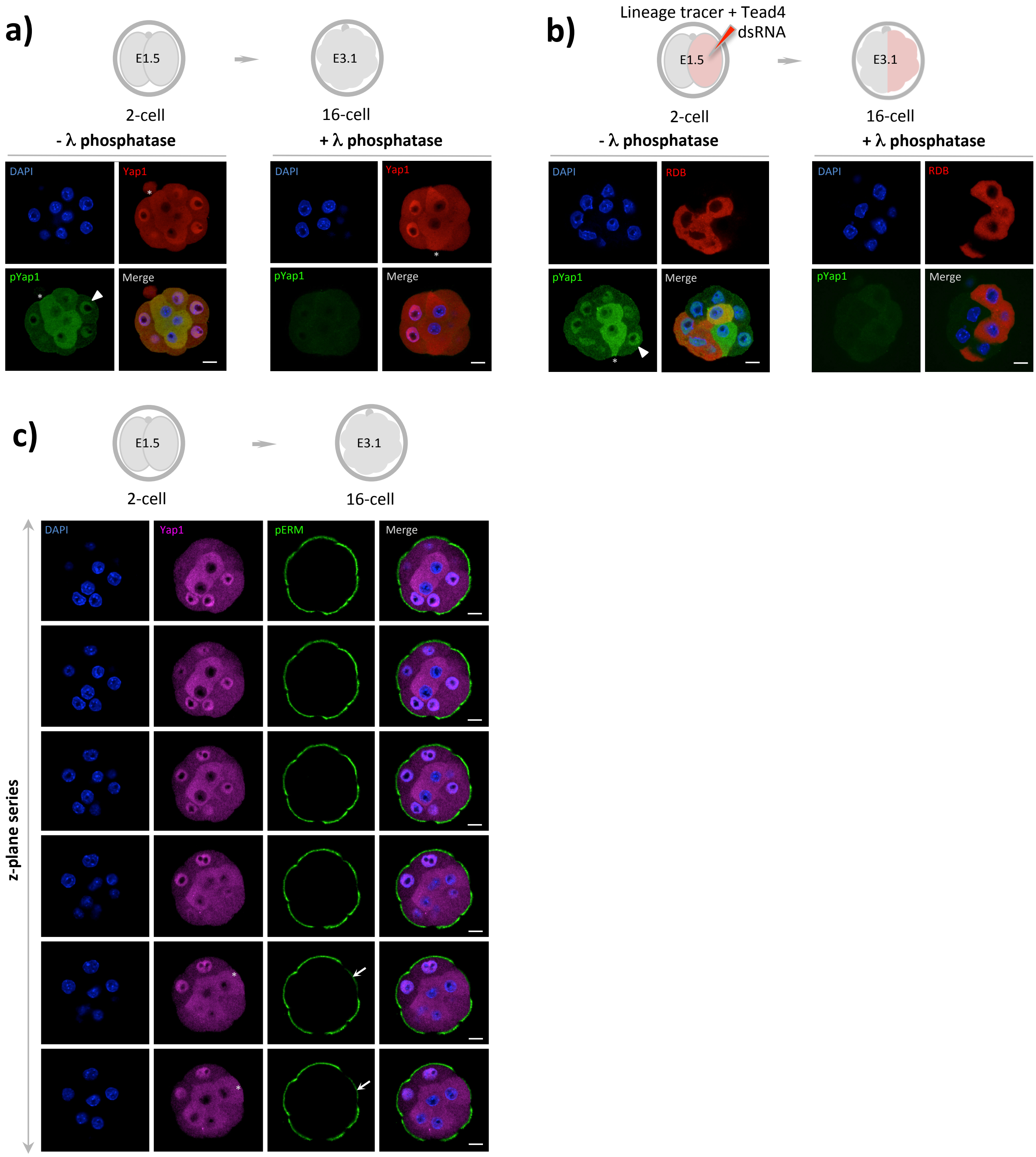

Supplementary figure S8: Yap1, phospho-Yap1 and phospho-ezrin (pERM) expression and intracellular localisation within non-perturbed and Tead4-specifc dsRNA microinjected (in one cell at the 2-cell stage) embryos at the mid-16-cell (E3.1) stage.

Supplementary figure S8: Yap1, phospho-Yap1 and phospho-ezrin (pERM) expression and intracellular localisation within non-perturbed and Tead4-specifc dsRNA microinjected (in one cell at the 2-cell stage) embryos at the mid-16-cell (E3.1) stage. a) Representative single confocal z-plane of mid-16-cell stage (E3.1) stage embryos, *in vitro* cultured from the 2-cell stage (E1.5) and double immuno-stained for Yap1 (red – *n.b.* anti-sera does not discriminate between phosphorylated and non-phosphorylated forms) and phosphorylated-Yap1 (green – pYap1). Note that the specificity of the anti-pYap1 anti-sera for only the phosphorylated isoform of Yap1 was confirmed by pre-incubating fixed embryos with λ phosphatase prior to immuno-staining (compare lack of signal in right panel with that in left panel). Yap1 immuno-staining (red) is enriched in the nuclei of outer-cells and the cytoplasm of inner-cells, with an occasional outer-cell displaying a cytoplasmic signal (see asterisks). pYap1 localisation is consistently cytoplasmic, with low level nuclear expression, in inner-cells (plus the occasional outer-cell – asterisk). However, it is also present within the nuclei of outer-cells, suggesting a basal level of nuclear Yap1 import that is irrespective of phosphorylation status (see arrow-head highlighted examples). **b)** Representative single confocal z-plane of mid-16-cell stage embryos, *in vitro* cultured after microinjection with RDB lineage tracer (red) plus Tead4-dsRNA in one cell at the 2-cell stage and immuno-stained for pYap1 (green). Right panel shows immuno-staining after pre-treatment with λ phosphatase and left panel without treatment. Note, pYap1 localisation is dictated by a cell's relative spatial position within the embryo (*i.e.* mainly cytoplasmic in inner-cells and nuclear in outer-cells) irrespective of whether the cell is derived from the microinjected, and hence *Tead4* KD, clone or non-microinjected clone. This result indicates that the mis-localisation of Yap1 protein (using the anti-sera that does not discriminate between phosphorylated and non-phosphorylated isoforms) observed after *Tead4* KD in mid-16-cell (E3.1) and 32-cell (E3.6) stage embryos (see figure 5) is not phosphorylated. **c)** Six consecutive confocal z-plane images of unperturbed 16-cell stage embryos, *in vitro* cultured from the 2-cell stage, and immuno-stained for Yap1 (pseudo-coloured magenta – *n.b.* anti-sera does not discriminate between phosphorylated and non-phosphorylated forms) and phospho-ezrin (green - using pERM antibody). Note nuclear localised Yap1 signal in the outer-cells accompanied by robust pERM immuno-staining in the apical domain. Conversely, Yap1 localisation is cytoplasmic and excluded from

the nucleus in encapsulated inner-cells. However, occasionally an outer-cell exhibiting cytoplasmic Yap1 localisation (akin to that normally observed in inner-cells – see asterisk) is observed and is associated with either reduced pERM immuno-staining (as in the example given – see arrow) or a very small exposed apical domain (data not shown). In relation to all panels of the figure, DNA was counter-stained with DAPI and the scale bars = 10μM.

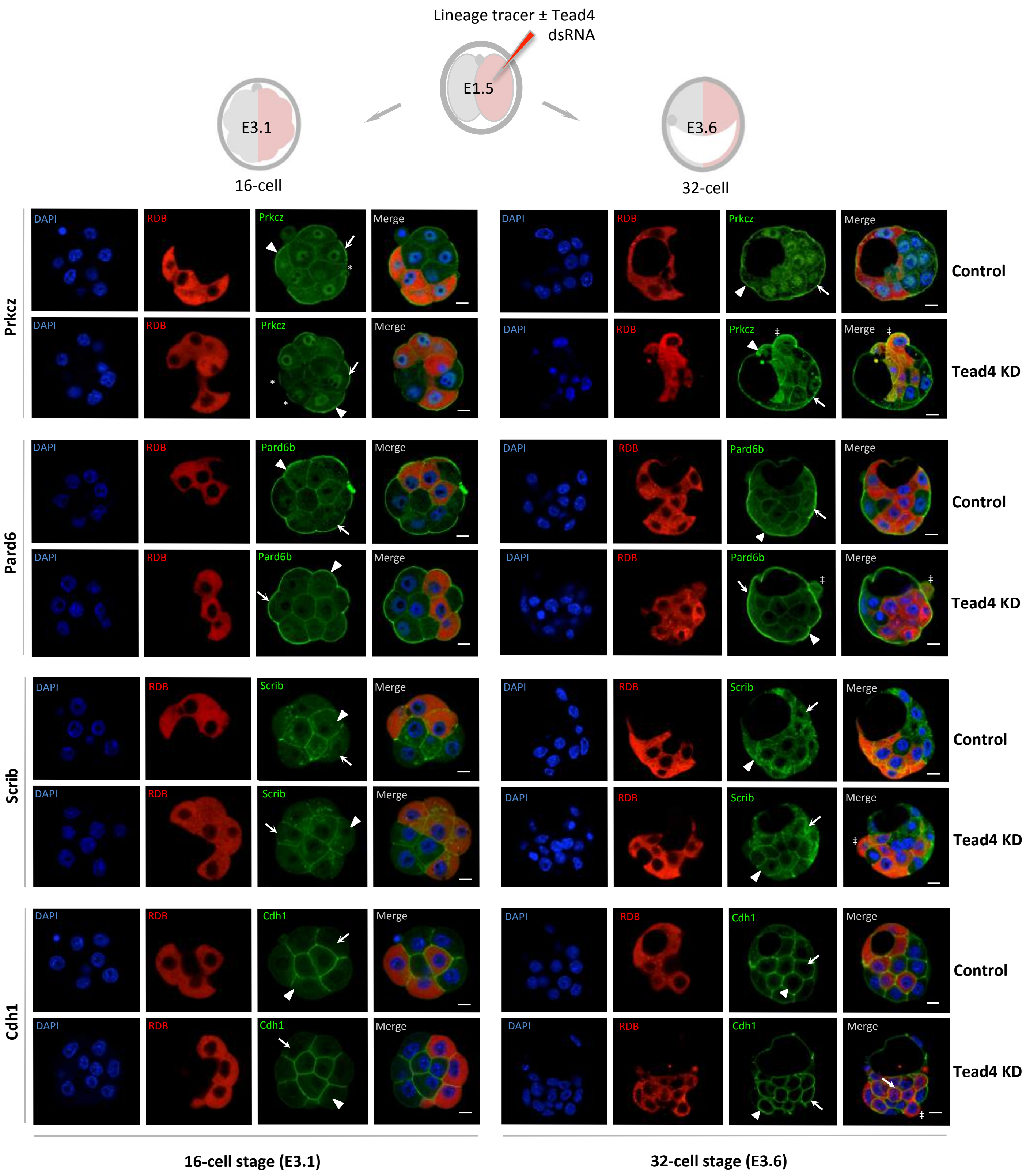

Supplementary figure S9: Clonal inhibition of TE differentiation via down-regulation of *Tead4* expression is not associated with reduced expression or non-apical/ basolateral protein localisation of the polarity marker genes (*Prkcz, Pard6b, Scrib* and *Cdh1*).

Supplementary figure S9: Clonal inhibition of TE differentiation via down-regulation of *Tead4* expression is not associated with reduced expression or non-apical/ basolateral protein localisation of the polarity marker genes (*Prkcz, Pard6b, Scrib* and *Cdh1*). 2-cell stage (E1.5) embryos were microinjected in one cell with RDB lineage tracer (red) ± Tead4-dsRNA and *in vitro* cultured to either the mid-16-cell (E3.1) or 32-cell (E3.6) stages. Embryos were then fixed and immuno-stained for either the apical polarity markers Prkcz and Pard6b (green; upper panels) or the basolateral polarity markers Scrib and Cdh1 (green; lower panels). DNA was counterstained with DAPI (blue). Arrows denote exemplar outer-cell apical domains immuno-stained for either Prkcz or Pard6b or basolateral domains immuno-stained for Scrib and Cdh1, derived from the non-microinjected cell clone of both control and *Tead4*-KD embryos. Whereas, arrow-heads highlight the same within the microinjected cell clone. Asterisks denote outer 16-cell stage embryo cells exhibiting reduced apical immuno-staining for either Prkcz or Pard6b that is not restricted to one or other clone in either control or *Tead4* KD experimental embryos. Double cross-hairs in merged images highlight outer-cells from the microinjected cell clone of *Tead4* KD embryos that exhibit atypical rounded morphology at the 32-cell stage (E3.6), irrespective of the primary antibody used in the immuno-staining procedure. However such cells are typically associated with enhanced apical Prkcz immuno-staining. Scale bars = 10μM. Note from the representative single confocal z-plane micrographs, that at the 16-cell stage there are no differences in the expression level or apical/ basolateral localisation of either Prkcz/ Pard6b or Scrib/ Cdh1 proteins, respectively, between the non-microinjected and microinjected (distinguishable by red fluorescence) cell clones of control or *Tead4* KD embryos. Although, as with pERM immuno-staining (see supplementary figure S8) an outer-cell lacking, or with greatly diminished, apically localised factors is occasionally observed (see asterisks in Prkcz panels). Moreover, note that the extent of apical/ basolateral localisation of the relevant factors is equal when comparing control and *Tead4* KD embryos directly. However at the 32-cell (E3.6) stage, enhanced apical polarity (associated with Prkcz) and atypical rounded morphology is observed within those members of the Tead4-dsRNA microinjected cell clone that did not internalise as a result of the fifth cleavage division (16- to 32-cell stage transition - see double crosshairs in micrographs) when compared against the non-microinjected outer-cell clone population or both clones in control embryos. By the >64-

cell (E4.5) stage, such cells undergo apoptosis (see supplementary figure S5 and supplementary tables ST6-ST8) or they can sometimes extrude their apical pole in an enucleated cell (data not shown).

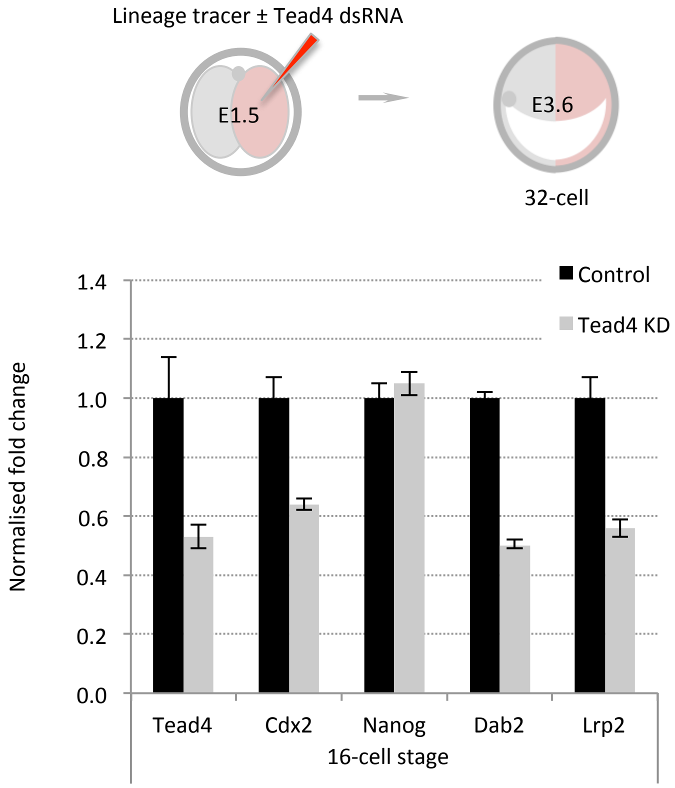

Supplementary Fig. S10: Clonal TE-inhibition; no enhanced Nanog mRNA expression at the to 32-cell stage but attenuated PrE-specific marker gene mRNA expression.

Supplementary figure S10: Clonal TE-inhibition; no enhanced Nanog mRNA expression at the to 32-cell stage but attenuated PrE-specific marker gene mRNA expression. The experimental strategy to down-regulate *Tead4* and inhibit TE-differentiation within a clone of cells representing half the total embryo cell number, prior to Q-RTPCR analysis at the early blastocyst (E3.6) stage (upper). Normalised expression fold changes, resulting from *Tead4*-KD, of the stated transcripts at the 32-cell (E3.6) stage (lower). Individual gene mRNA levels were normalised against Rpl23 and/ or H2afz transcript levels within control and experimental knockdown conditions prior to fold change calculation. Note Tead4, Cdx2, Dab2 and Lrp2 mRNA levels in clonal Tead4-KD/ TE-inhibited embryos are around half as abundant than in controls, whereas Nanog mRNA levels remain unaffected. Errors = s.e.m, n = at least 2 for biological and 3 for technical replicates.

a)

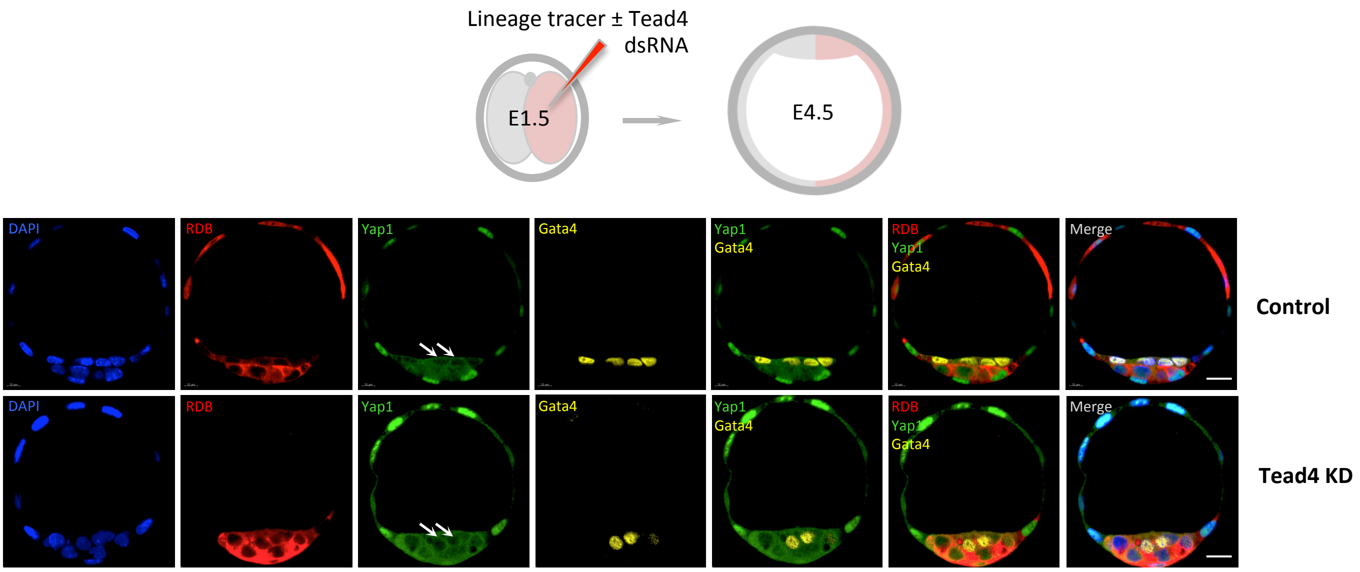

b)

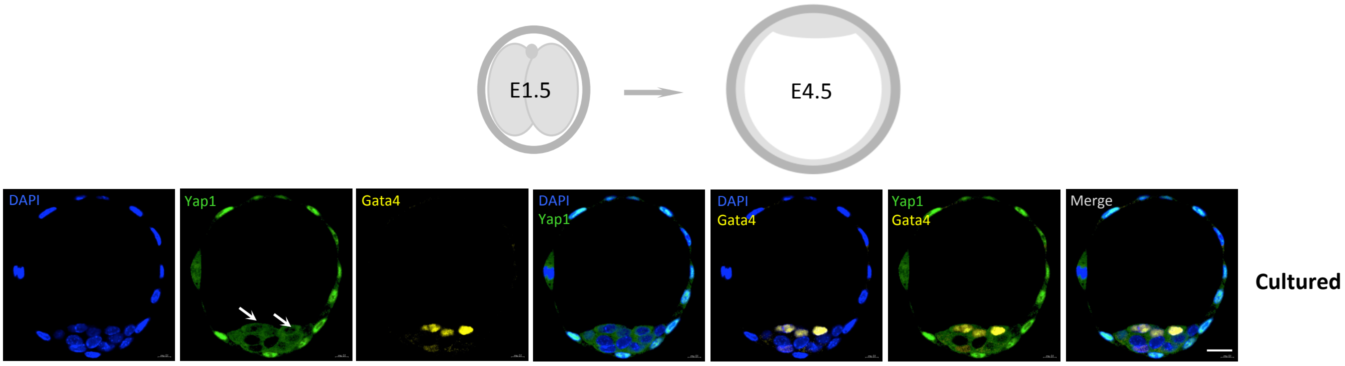

Supplementary figure S11: PrE cells, marked by Gata4 immuno-staining, both within and outwith the microinjected clone for *Tead4*-KD/ TE-inhibited or control embryos, and in *in vitro* cultured control embryos, exhibit cytoplasmic Yap1 localisation indicative of an active hippo-signalling pathway.

Supplementary figure S11: PrE cells, marked by Gata4 immuno-staining, both within and outwith the microinjected clone for *Tead4*-KD/ TE-inhibited or control embryos, and in *in vitro* cultured control embryos, exhibit cytoplasmic Yap1 localisation indicative of an active hippo-signalling pathway. a) Upper panel; Schematic of experimental strategy to clonally inhibit TE-differentiation in one-half of the developing embryos cells by microinjecting one cell at the 2-cell (E1.5) cell stage with RDBs ± Tead4-dsRNA and *in vitro* culturing to the late blastocyst (E4.5) stage, before assaying the activity of the hippo-signalling pathway in resulting PrE cells via immuno-fluorescent staining for Yap1 (anti-sera does not discriminate between phosphorylated and non-phosphorylated isoforms) and Gata4. Lower panel; representative single z-section confocal micrographs of microinjected late blastocyst (E4.5) stage embryos immuno-fluorescently stained for Yap1 (green) and Gata4 (pseudo-coloured yellow), with DNA counterstained with DAPI (blue). Cells derived from the microinjected clone are discernable by RBD fluorescence (red). Partial and fully merged images are shown to aid interpretation. Note that in both control and *Tead4*-KD embryos, active hippo-signalling, characterised by cytoplasmic Yap1, is preserved in all cells of the ICM, including Gata4 positive PrE cells (see arrows) **b)** As in **a)** but with the exception that immuno-fluorescent staining was performed on culture control embryos harvested at the 2-cell stage and *in vitro* cultured until the late blastocyst (E4.5) stage. Note, akin to examples in a), active hippo-signalling, characterised by cytoplasmic Yap1, in all cells of the ICM, including Gata4 positive PrE cells (see arrows). In all micrographs the scale bar is equivalent to 10μM.

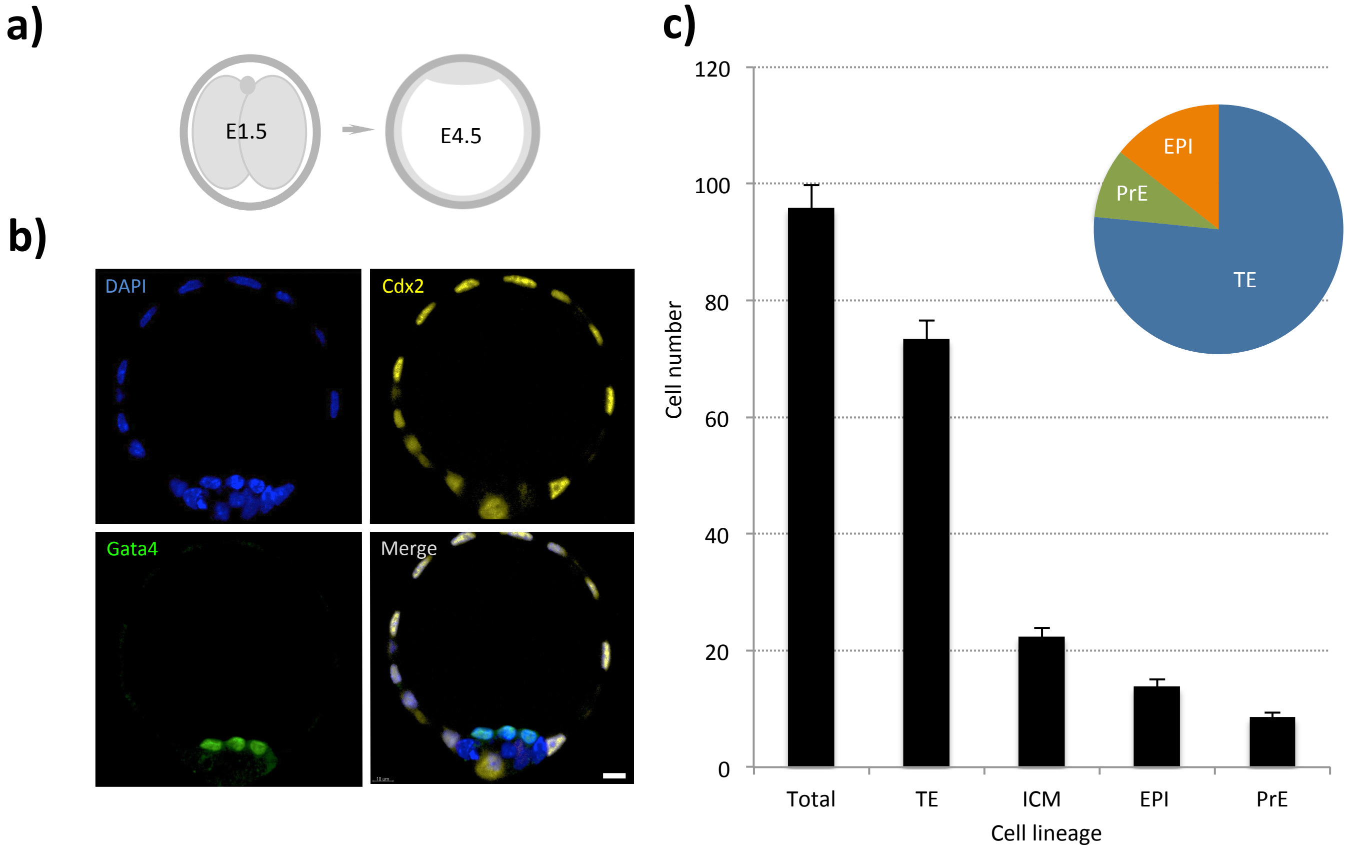

Supplementary figure S12: Relative cell lineage segregation in unperturbed preimplantation mouse embryos *in vitro* cultured from the 2-cell stage until the late blastocyst stage (E4.5); establishing an experimental baseline.

Supplementary figure S12: Relative cell lineage segregation in unperturbed preimplantation mouse embryos *in vitro* cultured from the 2-cell stage until the late blastocyst stage (E4.5); establishing an experimental baseline. a) Schematic representation of the *in vitro* culture period of recovered 2-cell (E1.5) stage embryos. **b)** Representative single confocal z-plane image through the centre of a cultured E4.5 stage late blastocyst immuno-stained for TE (Cdx2 – pseudo-coloured yellow) and PrE (Gata4 – green) lineage markers, plus DNA nuclear co-stain (DAPI – blue). Scale bar represents 10μM. **c)** Bar chart reporting the average cell number contribution to each late blastocyst (E4.5) stage cell lineage of *in vitro* cultured embryos (as described in a) above). Trophectoderm (TE) and primitive endoderm (PrE) contribution was assigned by presence of specific lineage marker immuno-staining (see b) above). Epiblast (EPI) contribution was determined by a lack of immuno-stain for either TE or PrE marker protein within the inner-cell mass (ICM; PrE + EPI). The average total number of cells is also given (n= 16 and error bars represent s.e.m.). Inset – pie chart detailing the same data in percentage format. See supplementary table ST13 for individual embryo data.

Supplementary tables ST1

RDB (1in2, IF: Cdx2/Gata4)

#	TOTAL NUMBER OF CELLS							NON-INJECTED CLONE				INJECTED CLONE			
	EMBRYO	TE	ICM			NON-INJECTED	INJECTED	OUTER	INNER			OUTER	INNER		
			EPI	PrE	TOTAL				Gata4 -	Gata4 +	TOTAL		Gata4 -	Gata4 +	TOTAL
1	94	74	13	7	20	49	45	39	7	3	10	35	6	4	10
2	108	73	29	6	35	45	63	30	12	3	15	43	17	3	20
3	86	70	12	4	16	37	49	33	3	1	4	37	9	3	12
4	110	83	14	13	27	50	60	35	8	7	15	48	6	6	12
5	108	82	14	12	26	67	41	50	10	7	17	32	4	5	9
6	92	72	13	7	20	41	51	34	3	4	7	38	10	3	13
7	89	72	6	11	17	43	46	38	2	3	5	34	4	8	12
8	108	80	14	14	28	57	51	41	7	9	16	39	7	5	12
9	89	68	12	9	21	38	51	29	6	3	9	39	6	6	12
10	79	58	13	8	21	47	32	32	11	4	15	26	2	4	6
11	120	78	26	16	42	69	51	42	17	10	27	36	9	6	15
12	92	69	18	5	23	39	53	29	9	1	10	40	9	4	13
13	77	57	7	13	20	37	40	27	4	6	10	30	3	7	10
14	89	76	5	8	13	45	44	44	1	0	1	32	4	8	12
15	94	64	17	13	30	40	54	27	9	4	13	37	8	9	17
16	94	73	12	9	21	54	40	39	10	5	15	34	2	4	6
17	90	70	12	8	20	48	42	40	6	2	8	30	6	6	12
18	75	51	20	4	24	36	39	27	9	0	9	24	11	4	15
19	84	60	15	9	24	50	34	37	8	5	13	23	7	4	11
20	85	60	16	9	25	44	41	32	7	5	12	28	9	4	13
21	88	58	20	10	30	41	47	26	10	5	15	32	10	5	15
22	97	64	25	8	33	40	57	25	13	2	15	39	12	6	18
23	104	80	17	7	24	55	49	45	9	1	10	35	8	6	14
24	98	74	19	5	24	54	44	40	10	4	14	34	9	1	10
25	97	63	25	9	34	55	42	31	18	6	24	32	7	3	10
26	97	85	7	5	12	42	55	37	3	2	5	48	4	3	7
27	114	81	24	9	33	41	73	29	3	9	12	52	21	0	21
28	112	83	17	12	29	62	50	43	10	9	19	40	7	3	10
29	111	88	11	12	23	58	53	44	5	9	14	44	6	3	9
30	99	82	12	5	17	48	51	40	5	3	8	42	7	2	9
**TOTAL**	2880	2148	465	267	732	1432	1448	1065	235	132	367	1083	230	135	365
**AVERAGE**	**96.0**	**71.6**	**15.5**	**8.9**	**24.4**	**47.7**	**48.3**	**35.5**	**7.8**	**4.4**	**12.2**	**36.1**	**7.7**	**4.5**	**12.2**
SEM	2.1	1.8	1.1	0.6	1.2	1.6	1.6	1.2	0.7	0.5	1.0	1.3	0.7	0.4	0.7
Stat. sig. (inter-clone) ‡p<0.05, ‡‡p<0.005															
p-value (2-tailed students t-test)							8.14E-01					7.34E-01	8.74E-01	8.77E-01	9.56E-01

GFP-dsRNA+RDB (1in2, IF: Cdx2/Gata4)

#	TOTAL NUMBER OF CELLS							NON-INJECTED CLONE				INJECTED CLONE			
	EMBRYO	TE	ICM			NON-INJECTED	INJECTED	OUTER	INNER			OUTER	INNER		
			EPI	PrE	TOTAL				Gata4 -	Gata4 +	TOTAL		Gata4 -	Gata4 +	TOTAL
1	93	67	12	14	26	48	45	35	8	5	13	32	4	9	13
2	99	66	27	6	33	49	50	33	16	0	16	33	11	6	17
3	82	62	11	9	20	42	40	32	6	4	10	30	5	5	10
4	97	74	19	4	23	50	47	33	16	1	17	41	3	3	6
5	93	64	23	6	29	49	44	33	15	1	16	31	8	5	13
6	102	81	12	9	21	49	53	41	4	4	8	40	8	5	13
7	106	79	14	13	27	50	56	34	7	9	16	45	7	4	11
8	95	78	8	9	17	49	46	42	2	5	7	36	6	4	10
9	109	82	16	11	27	53	56	42	5	6	11	40	11	5	16
10	102	74	13	15	28	48	54	40	1	7	8	34	12	8	20
11	98	81	8	9	17	39	59	28	7	4	11	53	1	5	6
12	95	74	12	9	21	44	51	34	7	3	10	40	5	6	11
13	100	72	16	12	28	54	46	41	10	3	13	31	6	9	15
14	82	64	11	7	18	41	41	34	6	1	7	30	5	6	11
15	99	79	14	6	20	49	50	42	5	2	7	37	9	4	13
16	102	86	8	8	16	53	49	43	6	4	10	43	2	4	6
17	87	62	16	9	25	49	38	37	11	1	12	25	5	8	13
18	103	75	23	5	28	50	53	34	13	3	16	41	10	2	12
19	83	63	16	4	20	35	48	26	5	4	9	37	11	0	11
20	88	59	25	4	29	53	35	44	9	0	9	15	16	4	20
21	90	68	12	10	22	45	45	34	4	7	11	34	8	3	11
22	81	67	10	4	14	35	46	31	2	2	4	36	8	2	10
23	86	66	15	5	20	42	44	33	7	2	9	33	8	3	11
24	116	88	23	5	28	63	53	49	12	2	14	39	11	3	14
25	88	59	20	9	29	43	45	27	11	5	16	32	9	4	13
26	106	76	19	11	30	49	57	38	7	4	11	38	12	7	19
27	91	64	16	11	27	48	43	31	11	6	17	33	5	5	10
28	97	72	19	6	25	47	50	44	2	1	3	28	17	5	22
29	84	54	26	4	30	43	41	28	12	3	15	26	14	1	15
30	91	62	24	5	29	51	40	34	13	4	17	28	11	1	12
31	104	78	20	6	26	50	54	41	5	4	9	37	15	2	17
32	99	76	14	9	23	51	48	36	12	3	15	40	2	6	8
33	99	75	13	11	24	52	47	37	9	6	15	38	4	5	9
34	101	79	17	5	22	52	49	41	8	3	11	38	9	2	11
35	102	80	18	4	22	54	48	42	9	3	12	38	9	1	10
36	86	60	15	11	26	45	41	30	5	10	15	30	10	1	11
37	96	72	18	6	24	40	56	23	13	4	17	49	5	2	7
**TOTAL**	3532	2638	603	291	894	1764	1768	1327	301	136	437	1311	302	155	457
**AVERAGE**	**95.5**	**71.3**	**16.3**	**7.9**	**24.2**	**47.7**	**47.8**	**35.9**	**8.1**	**3.7**	**11.8**	**35.4**	**8.2**	**4.2**	**12.4**
SEM	1.4	1.4	0.8	0.5	0.8	0.9	1.0	1.0	0.7	0.4	0.6	1.1	0.6	0.4	0.6
Stat. sig. (exp. vs. con embryo) *p<0.05, **p<0.005															
p-value (2-tailed students t-test)	8.31E-01	8.93E-01	5.69E-01	1.86E-01	8.71E-01	9.76E-01	7.93E-01	8.16E-01	7.63E-01	2.66E-01	7.25E-01	6.96E-01	6.15E-01	5.60E-01	8.42E-01
Stat. sig. (inter-clone) ‡p<0.05, ‡‡p<0.005															
p-value (2-tailed students t-test)							9.35E-01					7.74E-01	9.77E-01	3.39E-01	5.50E-01

Supplementary tables ST1: Quantified cell lineage segregation in individual late blastocyst stage (E4.5) embryos cultured *in vitro* from 2-cell stage (E1.5) embryos microinjected, in a single cell, with fluorescent RDB tracer alone or RDB and GFP-dsRNA, (immuno-stained for Cdx2 and Gata4) *– relating to supplementary figure S1.* The two tables report the total number of cells for each individual late blastocyst stage (E4.5) embryo, within each lineage (TE – light blue, EPI – yellow, PrE – green, ICM – dark blue and total cell number - olive), for embryos microinjected, in one cell at the 2-cell stage (E1.5), with either fluorescent RDB lineage tracer alone ('RDB 1in2' – upper table) or RDBs plus GFP-dsRNA ('GFP-dsRNA+RDB 1in2'– lower table). Additionally the total number of cells within both the non-microinjected (white) and microinjected (red) clones is also given. On the right of the tables, the contribution of cells from the non-microinjected and microinjected cell clones, for each individual embryo, to each blastocyst lineage is also given. The averaged data (s.e.m. = standard error of the mean), as used in supplementary figure S1, is also provided. In both RDB alone and GFP-dsRNA+RDB control tables, the averaged data is accompanied by p-values (resulting from 2-tailed students t-test) describing the statistical significance of inter-clone differences for each lineage plus the total cell clone count (‡ = $p<0.05$ and ‡‡ = $p<0.005$). In regard to the tabulated *Tead4* KD data, p-values (2-tailed student t-test) describing the statistical significance of differences between equivalent cell lineages and cell clones between the averaged data of control and *Tead4* KD embryos is also provided (* = $p<0.05$ and ** = $p<0.005$).

Supplementary tables ST2

RDB (1in2, IF: Cdx2/ Gata4) apoptotic cells

#	TOTAL NUMBER OF CELLS					NON-INJECTED CLONE		INJECTED CLONE	
	EMBRYO	TE	ICM	NON-INJECTED	INJECTED	OUTER	ICM	OUTER	ICM
1	4	2	2	1	3	0	1	2	1
2	3	1	2	2	1	1	1	0	1
3	11	6	5	6	5	3	3	3	2
4	7	4	3	3	4	2	1	2	2
5	5	1	4	3	2	1	2	0	2
6	4	3	1	1	3	1	0	2	1
7	9	2	7	5	4	1	4	1	3
8	6	2	4	4	2	2	2	0	2
9	9	2	7	3	6	0	3	2	4
10	12	4	8	8	4	3	5	1	3
11	5	1	4	4	1	1	3	0	1
12	6	5	1	5	1	4	1	1	0
13	12	4	8	4	8	1	3	3	5
14	11	4	7	5	6	1	4	3	3
15	6	4	2	3	3	3	0	1	2
16	4	1	3	4	0	1	3	0	0
17	5	2	3	4	1	2	2	0	1
18	11	6	5	7	4	4	3	2	2
19	6	0	6	3	3	0	3	0	3
20	9	6	3	3	6	2	1	4	2
21	12	3	9	4	8	0	4	3	5
22	5	2	3	0	5	0	0	2	3
23	5	4	1	0	5	0	0	4	1
24	13	7	6	7	6	4	3	3	3
25	6	0	6	2	4	0	2	0	4
26	10	5	5	6	4	4	2	1	3
27	7	3	4	4	3	3	1	0	3
28	3	0	3	2	1	0	2	0	1
29	7	4	3	5	2	3	2	1	1
30	3	2	1	2	1	2	0	0	1
TOTAL	216	90	126	110	106	49	61	41	65
AVERAGE	7.2	3.0	4.2	3.7	3.5	1.6	2.0	1.4	2.2
SEM	0.6	0.4	0.4	0.4	0.4	0.3	0.3	0.2	0.2
Stat. sig. (inter-clone) ‡p<0.05, ‡‡p<0.005									
p-value (2-tailed students t-test)					8.03E-01			4.52E-01	7.03E-01

GFP-dsRNA+RDB (1in2, IF: Cdx2/ Gata4) apoptotic cells

#	TOTAL NUMBER OF CELLS					NON-INJECTED CLONE		INJECTED CLONE	
	EMBRYO	TE	ICM	NON-INJECTED	INJECTED	OUTER	ICM	OUTER	ICM
1	11	4	7	6	5	3	3	1	4
2	8	1	7	5	3	1	4	0	3
3	17	6	11	8	9	3	5	3	6
4	3	3	0	0	3	0	0	3	0
5	9	4	5	4	5	2	2	2	3
6	7	2	5	3	4	1	2	1	3
7	8	0	8	7	1	0	7	0	1
8	6	3	3	3	3	0	3	3	0
9	4	0	4	2	2	0	2	0	2
10	7	2	5	6	1	2	4	0	1
11	11	4	7	7	4	3	4	1	3
12	11	2	9	6	5	1	5	1	4
13	6	1	5	1	5	0	1	1	4
14	5	3	2	2	3	2	0	1	2
15	6	5	1	6	0	5	1	0	0
16	8	4	4	3	5	1	2	3	2
17	9	3	6	5	4	2	3	1	3
18	7	1	6	4	3	1	3	0	3
19	4	2	2	1	3	0	1	2	1
20	20	12	8	8	12	7	1	5	7
21	8	3	5	5	3	1	4	2	1
22	10	6	4	7	3	3	4	3	0
23	5	1	4	3	2	1	2	0	2
24	8	5	3	7	1	5	2	0	1
25	4	1	3	3	1	1	2	0	1
26	7	3	4	4	3	2	2	1	2
27	13	7	6	6	7	3	3	4	3
28	15	6	9	7	8	5	2	1	7
29	3	0	3	2	1	0	2	0	1
30	3	1	2	3	0	1	2	0	0
31	1	0	1	1	0	0	1	0	0
32	9	1	8	3	6	0	3	1	5
33	9	6	3	4	5	2	2	4	1
34	5	2	3	3	2	1	2	1	1
35	7	2	5	3	4	1	2	1	3
36	8	6	2	3	5	2	1	4	1
37	8	6	2	4	4	3	1	3	1
TOTAL	290	118	172	155	135	65	90	53	82
AVERAGE	7.8	3.2	4.6	4.2	3.6	1.8	2.4	1.4	2.2
SEM	0.6	0.4	0.4	0.4	0.4	0.3	0.2	0.2	0.3
Stat. sig. (exp. vs. con embryo) *p<0.05, **p<0.005									
p-value (2-tailed students t-test)	4.59E-01	7.30E-01	4.53E-01	3.04E-01	8.41E-01	7.45E-01	2.56E-01	8.47E-01	9.00E-01
Stat. sig. (inter-clone) ‡p<0.05, ‡‡p<0.005									
p-value (2-tailed students t-test)					3.26E-01			3.78E-01	5.84E-01

Supplementary tables ST2: Incidence of apoptotic cells within individual late blastocyst stage (E4.5) embryos *in vitro* cultured from 2-cell stage (E1.5) embryos microinjected, in a single cell, with fluorescent RDB lineage tracer alone or RDB and GFP-dsRNA (immuno-stained for Cdx2 and Gata4) – *relating to supplementary figure S1.* The tables reports the total number of apoptotic cells (in olive), for each individual late blastocyst stage (E4.5) embryo, within each spatial compartment (*i.e.* 'TE/ outer' – light blue or encapsulated 'ICM' – dark blue), for embryos microinjected, in one cell at the 2-cell stage (E1.5), with either fluorescent RDB lineage tracer alone ('RDB 1in2 apoptotic cells' – upper table) or RDBs plus Tead4-dsRNA ('GFP-dsRNA+RDB 1in2 apoptotic cells' – lower table) – *i.e.* those embryos referred to in supplementary figure S1 and supplementary tables ST1. Additionally, the total number of apoptotic cells within both the non-microinjected (white) and microinjected (red) clones is also given. On the right of the tables, the incidence of apoptotic cells from the non-microinjected and microinjected cell clones, for each individual embryo, within either spatial compartment is reported. The averaged data (s.e.m. = standard error of the mean) is also provided. In both control and *Tead4* KD tables, the averaged data is accompanied by p-values (resulting from 2-tailed students t-test) describing the statistical significance of inter-clone differences for each spatial compartment (‡ = p<0.05 and ‡‡ = p<0.005). In regard to the tabulated *Tead4* KD data, p-values (2-tailed student t-test) describing the statistical significance of differences between equivalent spatial compartments and cell clones between the averaged data of control and *Tead4* KD embryos is also provided (* = p<0.05 and ** = p<0.005).

Supplementary tables ST3

Control (1in2, IF: Cdx2/ Gata4)

#	TOTAL NUMBER OF CELLS							NON-INJECTED CLONE				INJECTED CLONE			
	EMBRYO	TE	ICM			NON-INJECTED	INJECTED	OUTER	INNER			OUTER	INNER		
			EPI	PrE	TOTAL				Gata4 -	Gata4 +	TOTAL		Gata4 -	Gata4 +	TOTAL
1	76	61	11	4	15	25	51	16	6	3	9	45	5	1	6
2	89	76	7	6	13	46	43	37	5	4	9	39	2	2	4
3	80	56	20	4	24	54	26	36	16	2	18	20	4	2	6
4	86	60	21	5	26	37	49	24	12	1	13	36	9	4	13
5	80	58	15	7	22	42	38	34	5	3	8	24	10	4	14
6	81	52	14	15	29	49	32	29	12	8	20	23	2	7	9
7	79	63	11	5	16	43	36	34	6	3	9	29	5	2	7
8	83	54	22	7	29	46	37	23	18	5	23	31	4	2	6
9	100	79	12	9	21	49	51	42	4	3	7	37	8	6	14
10	87	65	14	8	22	36	51	32	1	3	4	33	13	5	18
11	107	83	17	7	24	70	37	55	11	4	15	28	6	3	9
12	106	78	19	9	28	52	54	38	8	6	14	40	11	3	14
13	78	51	20	7	27	43	35	30	10	3	13	21	10	4	14
14	92	73	12	7	19	31	61	26	4	1	5	47	8	6	14
15	85	65	12	8	20	37	48	27	6	4	10	38	6	4	10
16	96	70	21	5	26	50	46	35	12	3	15	35	9	2	11
17	71	47	19	5	24	40	31	27	8	5	13	20	11	0	11
18	90	74	6	10	16	59	31	46	4	9	13	28	2	1	3
19	94	72	7	15	22	42	52	34	0	8	8	38	7	7	14
20	112	92	10	10	20	72	40	57	9	6	15	35	1	4	5
21	89	59	23	7	30	44	45	31	11	2	13	28	12	5	17
22	89	70	12	7	19	34	55	28	4	2	6	42	8	5	13
23	75	58	12	5	17	39	36	32	5	2	7	26	7	3	10
24	96	82	9	5	14	44	52	41	3	0	3	41	6	5	11
**TOTAL**	2121	1598	346	177	523	1084	1037	814	180	90	270	784	166	87	253
**AVERAGE**	**88.4**	**66.6**	**14.4**	**7.4**	**21.8**	**45.2**	**43.2**	**33.9**	**7.5**	**3.8**	**11.3**	**32.7**	**6.9**	**3.6**	**10.5**
SEM	2.2	2.4	1.1	0.6	1.0	2.2	1.9	1.9	0.9	0.5	1.0	1.6	0.7	0.4	0.8
Stat. sig. (inter-clone) ‡p<0.05, ‡‡p<0.005															
p-value (2-tailed students t-test)							5.07E-01					6.23E-01	6.15E-01	8.39E-01	5.97E-01

Tead4 KD (1in2, IF: Cdx2/ Gata4)

#	TOTAL NUMBER OF CELLS							NON-INJECTED CLONE				INJECTED CLONE			
	EMBRYO	TE	ICM			NON-INJECTED	INJECTED	OUTER	INNER			OUTER	INNER		
			EPI	PrE	TOTAL				Gata4 -	Gata4 +	TOTAL		Gata4 -	Gata4 +	TOTAL
1	70	44	20	6	26	49	21	42	1	6	7	2	19	0	19
2	76	46	26	4	30	48	28	40	7	1	8	6	19	3	22
3	72	48	19	5	24	43	29	40	0	3	3	8	19	2	21
4	82	49	27	6	33	49	33	40	5	4	9	9	22	2	24
5	104	64	33	7	40	45	59	37	4	4	8	27	29	3	32
6	72	41	27	4	31	32	40	19	10	3	13	22	17	1	18
7	77	44	29	4	33	42	35	29	9	4	13	15	20	0	20
8	90	50	22	18	40	46	44	42	1	3	4	8	21	15	36
9	71	42	25	4	29	32	39	28	3	1	4	14	22	3	25
10	78	58	12	8	20	45	33	43	1	1	2	15	11	7	18
11	80	51	17	12	29	44	36	39	1	4	5	12	16	8	24
12	91	69	16	6	22	61	30	60	0	1	1	9	16	5	21
13	79	52	17	10	27	51	28	38	5	8	13	14	12	2	14
14	86	64	12	10	22	58	28	55	0	3	3	9	12	7	19
15	96	61	29	6	35	54	42	51	1	2	3	10	28	4	32
16	88	62	20	6	26	56	32	51	4	1	5	11	16	5	21
17	71	42	20	9	29	40	31	37	2	1	3	5	18	8	26
18	94	65	25	4	29	66	28	56	7	3	10	9	18	1	19
19	67	39	23	5	28	39	28	32	4	3	7	7	19	2	21
20	67	43	17	7	24	40	27	35	1	4	5	8	16	3	19
21	91	57	17	17	34	49	42	43	0	6	6	14	17	11	28
22	89	52	32	5	37	50	39	41	5	4	9	11	27	1	28
23	92	61	20	11	31	57	35	47	3	7	10	14	17	4	21
24	88	57	18	13	31	61	27	45	9	7	16	12	9	6	15
**TOTAL**	1971	1261	523	187	710	1157	814	990	83	84	167	271	440	103	543
**AVERAGE**	**82.1**	**52.5**	**21.8**	**7.8**	**29.6**	**48.2**	**33.9**	**41.3**	**3.5**	**3.5**	**7.0**	**11.3**	**18.3**	**4.3**	**22.6**
SEM	2.1	1.8	1.2	0.8	1.1	1.8	1.6	1.9	0.6	0.4	0.8	1.1	1.0	0.7	1.1
Stat. sig. (exp. vs. con embryo) *p<0.05, **p<0.005	*	**	**		**		**	*	**		**	**	**		**
p-value (2-tailed students t-test)	4.35E-02	2.68E-05	2.94E-05	6.82E-01	4.53E-06	2.94E-01	5.39E-04	9.59E-03	8.41E-04	6.96E-01	2.19E-03	1.31E-13	1.33E-11	4.31E-01	5.68E-11
Stat. sig. (inter-clone) ‡p<0.05, ‡‡p<0.005							‡‡					‡‡	‡‡		‡‡
p-value (2-tailed students t-test)							4.06E-07					6.44E-16	4.66E-15	3.60E-01	2.07E-14

Supplementary tables ST3: Quantified cell lineage segregation in individual late blastocyst stage (E4.5) embryos cultured *in vitro* from 2-cell stage (E1.5) embryos microinjected, in a single cell, with fluorescent RDB tracer ± Tead4-dsRNA (immuno-stained for Cdx2 and Gata4) – *relating to figure 3 and supplementary figure S2.* The two tables report the total number of cells for each individual late blastocyst stage (E4.5) embryo, within each lineage (TE – light blue, EPI – yellow, PrE – green, ICM – dark blue and total cell number - olive), for embryos microinjected, in one cell at the 2-cell stage (E1.5), with either fluorescent RDB lineage tracer alone (‘control 1in2’ – upper table) or RDBs plus Tead4-dsRNA (‘*Tead4* KD 1in2’– lower table). Additionally the total number of cells within both the non-microinjected (white) and microinjected (red) clones is also given. On the right of the tables, the contribution of cells from the non-microinjected and microinjected cell clones, for each individual embryo, to each blastocyst lineage is also given. The averaged data (s.e.m. = standard error of the mean), as used in figure 3 and supplementary figure S2, is also provided. In both control and *Tead4* KD tables, the averaged data is accompanied by p-values (resulting from 2-tailed students t-test) describing the statistical significance of inter-clone differences for each lineage plus the total cell clone count (‡ = $p<0.05$ and ‡‡ = $p<0.005$). In regard to the tabulated *Tead4* KD data, p-values (2-tailed student t-test) describing the statistical significance of differences between equivalent cell lineages and cell clones between the averaged data of control and *Tead4* KD embryos is also provided (* = $p<0.05$ and ** = $p<0.005$).

Supplementary tables ST4

Control (1in2, IF: Cdx2/ Sox17)

#	TOTAL NUMBER OF CELLS							NON-INJECTED CLONE				INJECTED CLONE			
	EMBRYO	TE	ICM			NON-INJECTED	INJECTED	OUTER	INNER			OUTER	INNER		
			EPI	PrE	TOTAL				Sox17 -	Sox17 +	TOTAL		Sox17 -	Sox17 +	TOTAL
1	89	71	8	10	18	48	41	37	6	5	11	34	2	5	7
2	107	83	14	10	24	44	63	36	3	5	8	47	11	5	16
3	96	78	12	6	18	30	66	23	4	3	7	55	8	3	11
4	86	69	4	13	17	38	48	29	4	5	9	40	0	8	8
5	100	81	10	9	19	39	61	33	2	4	6	48	8	5	13
6	113	101	4	8	12	58	55	53	1	4	5	48	3	4	7
7	113	85	23	5	28	55	58	45	8	2	10	40	15	3	18
8	113	91	11	11	22	60	53	44	9	7	16	47	2	4	6
9	82	66	12	4	16	56	26	51	5	0	5	15	7	4	11
10	105	82	12	11	23	54	51	44	5	5	10	38	7	6	13
11	98	72	14	12	26	51	47	33	8	10	18	39	6	2	8
12	95	69	22	4	26	40	55	31	8	1	9	38	14	3	17
13	99	85	2	12	14	50	49	46	1	3	4	39	1	9	10
**TOTAL**	1296	1033	148	115	263	623	673	505	64	54	118	528	84	61	145
**AVERAGE**	**99.7**	**79.5**	**11.4**	**8.8**	**20.2**	**47.9**	**51.8**	**38.8**	**4.9**	**4.2**	**9.1**	**40.6**	**6.5**	**4.7**	**11.2**
SEM	2.8	2.8	1.7	0.9	1.4	2.5	2.9	2.5	0.8	0.7	1.2	2.7	1.3	0.6	1.1
Stat. sig. (inter-clone) ‡p<0.05, ‡‡p<0.005															
p-value (2-tailed students t-test)							3.26E-01					6.34E-01	3.30E-01	5.59E-01	2.07E-01

Tead4 KD (1in2, IF: Cdx2/ Sox17)

#	TOTAL NUMBER OF CELLS							NON-INJECTED CLONE				INJECTED CLONE			
	EMBRYO	TE	ICM			NON-INJECTED	INJECTED	OUTER	INNER			OUTER	INNER		
			EPI	PrE	TOTAL				Sox17 -	Sox17 +	TOTAL		Sox17 -	Sox17 +	TOTAL
1	95	65	26	4	30	51	44	49	1	1	2	16	25	3	28
2	91	66	16	9	25	55	36	52	2	1	3	14	14	8	22
3	91	64	20	7	27	59	32	49	4	6	10	15	16	1	17
4	73	48	19	6	25	43	30	40	1	2	3	8	18	4	22
5	84	56	21	7	28	48	36	43	3	2	5	13	18	5	23
6	84	56	22	6	28	59	25	52	4	3	7	4	18	3	21
7	82	57	21	4	25	53	29	45	5	3	8	12	16	1	17
8	70	52	10	8	18	45	25	41	0	4	4	11	10	4	14
9	71	47	18	6	24	41	30	35	2	4	6	12	16	2	18
**TOTAL**	741	511	173	57	230	454	287	406	22	26	48	105	151	31	182
**AVERAGE**	**82.3**	**56.8**	**19.2**	**6.3**	**25.6**	**50.4**	**31.9**	**45.1**	**2.4**	**2.9**	**5.3**	**11.7**	**16.8**	**3.4**	**20.2**
SEM	3.1	2.4	1.5	0.6	1.1	2.2	2.0	2.0	0.6	0.5	0.9	1.2	1.3	0.7	1.4
Stat. sig. (exp. vs. con embryo) *p<0.05, **p<0.005	**	**	**	*	*		**		*		*	**	**		**
p-value (2-tailed students t-test)	5.90E-04	4.58E-06	2.65E-03	2.54E-02	7.62E-03	4.61E-01	1.70E-05	6.27E-02	1.63E-02	1.73E-01	1.79E-02	2.60E-08	2.72E-05	1.93E-01	9.32E-05
Stat. sig. (inter-clone) ‡p<0.05, ‡‡p<0.005							‡‡					‡‡	‡‡		‡‡
p-value (2-tailed students t-test)							1.33E-05					1.30E-09	9.80E-07	5.49E-01	4.23E-07

Supplementary tables ST4: Quantified cell lineage segregation in individual late blastocyst stage (E4.5) embryos cultured *in vitro* from 2-cell stage (E1.5) embryos microinjected, in a single cell, with fluorescent RDB tracer ± Tead4-specific dsRNA, (immuno-stained for Cdx2 and Sox17) – *relating to figure 3 and supplementary figure S3*. The two tables report the total number of cells for each individual late blastocyst stage (E4.5) embryo, within each lineage (TE – light blue, EPI – yellow, PrE – green, ICM – dark blue and total cell number - olive), for embryos microinjected, in one cell at the 2-cell stage (E1.5), with either fluorescent RDB lineage tracer alone ('control 1in2 IF: Cdx2/ Sox17' – upper table) or RDBs plus Tead4-dsRNA ('*Tead4* KD 1in2 IF: Cdx2/ Sox17'– lower table). Additionally the total number of cells within both the non-microinjected (white) and microinjected (red) clones is also given. On the right of the tables, the contribution of cells from the non-microinjected and microinjected cell clones, for each individual embryo, to each blastocyst lineage is also given. The averaged data (s.e.m. = standard error of the mean), as used in figure 3 and supplementary figure S3, is also provided. In both control and *Tead4* KD tables, the averaged data is accompanied by p-values (resulting from 2-tailed students t-test) describing the statistical significance of inter-clone differences for each lineage plus the total cell clone count (‡ = $p<0.05$ and ‡‡ = $p<0.005$). In regard to the tabulated *Tead4* KD data, p-values (2-tailed student t-test) describing the statistical significance of differences between equivalent cell lineages and cell clones between the averaged data of control and *Tead4* KD embryos is also provided (* = $p<0.05$ and ** = $p<0.005$).

Supplementary tables ST5

Control (1in2, IF: Gata4/ Nanog)

#	TOTAL NUMBER OF CELLS										
	EMBRYO	OUTER			INNER					NON-INJECTED	INJECTED
		Nanog-Gata4-	Nanog+	TOTAL	Nanog+	Gata4+	Nanog+Gata4+	Nanog-Gata4-	TOTAL		
1	101	79	4	83	11	7	0	0	18	47	54
2	98	70	11	81	11	6	0	0	17	49	49
3	91	75	2	77	7	7	0	0	14	43	48
4	76	60	2	62	8	6	0	0	14	43	33
5	96	71	0	71	11	11	1	2	25	49	47
6	76	51	2	53	17	6	0	0	23	43	33
7	116	98	2	100	8	6	0	2	16	69	47
8	107	78	4	82	16	8	0	1	25	68	39
9	102	77	0	77	8	16	1	0	25	49	53
10	92	60	14	74	14	4	0	0	18	43	49
11	89	74	0	74	10	5	0	0	15	45	44
12	99	70	6	76	14	9	0	0	23	49	50
13	82	55	3	58	15	7	0	2	24	38	44
14	90	69	0	69	9	10	1	1	21	56	34
15	101	68	3	71	22	5	0	3	30	45	56
16	117	74	19	93	12	11	0	1	24	53	64
17	93	62	6	68	14	11	0	0	25	39	54
18	102	65	6	71	22	5	4	0	31	42	60
19	110	82	7	89	11	10	0	0	21	56	54
20	103	52	25	77	21	4	0	1	26	52	51
21	108	72	7	79	18	11	0	0	29	46	62
22	109	82	1	83	14	10	0	2	26	59	50
23	112	75	8	83	18	10	1	0	29	60	52
24	113	78	7	85	21	6	0	1	28	59	54
25	97	70	8	78	12	5	0	2	19	52	45
**TOTAL**	2480	1767	147	1914	344	196	8	18	566	1254	1226
**AVERAGE**	**99.2**	**70.7**	**5.9**	**76.6**	**13.8**	**7.8**	**0.3**	**0.7**	**22.6**	**50.2**	**49.0**
SEM	2.3	2.1	1.2	2.1	0.9	0.6	0.2	0.2	1.0	1.7	1.6
Stat. sig. (inter-clone) ‡p<0.05, ‡‡p<0.005											
p-value (2-tailed students t-test)											6.32E-01

#	NON-INJECTED CLONE								INJECTED CLONE							
	OUTER			INNER					OUTER			INNER				
	Nanog-Gata4-	Nanog+	TOTAL	Nanog+	Gata4+	Nanog+Gata4+	Nanog-Gata4-	TOTAL	Nanog-Gata4-	Nanog+	TOTAL	Nanog+	Gata4+	Nanog+Gata4+	Nanog-Gata4-	TOTAL
1	36	2	38	6	3	0	0	9	43	2	45	5	4	0	0	9
2	28	6	34	10	5	0	0	15	42	5	47	1	1	0	0	2
3	36	1	37	6	0	0	0	6	39	1	40	1	7	0	0	8
4	33	2	35	2	6	0	0	8	27	0	27	6	0	0	0	6
5	38	0	38	5	4	0	2	11	33	0	33	6	7	1	0	14
6	29	2	31	12	0	0	0	12	22	0	22	5	6	0	0	11
7	57	0	57	6	4	0	2	12	41	2	43	2	2	0	0	4
8	55	3	58	6	4	0	0	10	23	1	24	10	4	0	1	15
9	34	0	34	4	10	1	0	15	43	0	43	4	6	0	0	10
10	24	11	35	7	1	0	0	8	36	3	39	7	3	0	0	10
11	31	0	31	10	4	0	0	14	43	0	43	0	1	0	0	1
12	36	2	38	6	5	0	0	11	34	4	38	8	4	0	0	12
13	25	0	25	8	4	0	1	13	30	3	33	7	3	0	1	11
14	46	0	46	4	4	1	1	10	23	0	23	5	6	0	0	11
15	32	2	34	5	4	0	2	11	36	1	37	17	1	0	1	19
16	27	7	34	10	8	0	1	19	47	12	59	2	3	0	0	5
17	31	0	31	3	5	0	0	8	31	6	37	11	6	0	0	17
18	29	4	33	5	3	1	0	9	36	2	38	17	2	3	0	22
19	37	5	42	8	6	0	0	14	45	2	47	3	4	0	0	7
20	26	12	38	12	2	0	0	14	26	13	39	9	2	0	1	12
21	34	2	36	7	3	0	0	10	38	5	43	11	8	0	0	19
22	48	0	48	7	4	0	0	11	34	1	35	7	6	0	2	15
23	40	2	42	11	6	1	0	18	35	6	41	7	4	0	0	11
24	38	4	42	13	3	0	1	17	40	3	43	8	3	0	0	11
25	39	3	42	6	3	0	1	10	31	5	36	6	2	0	1	9
**TOTAL**	889	70	959	179	101	4	11	295	878	77	955	165	95	4	7	271
**AVERAGE**	**35.6**	**2.8**	**38.4**	**7.2**	**4.0**	**0.2**	**0.4**	**11.8**	**35.1**	**3.1**	**38.2**	**6.6**	**3.8**	**0.2**	**0.3**	**10.8**
SEM	1.7	0.7	1.5	0.6	0.4	0.1	0.1	0.7	1.4	0.7	1.7	0.9	0.4	0.1	0.1	1.0
Stat. sig. (inter-clone) ‡p<0.05, ‡‡p<0.005																
p-value (2-tailed students t-test)									8.45E-01	7.70E-01	9.44E-01	5.97E-01	7.02E-01	1.00E+00	3.76E-01	4.42E-01

Tead4 KD (1in2, IF: Gata4/ Nanog)

#	TOTAL NUMBER OF CELLS										
	EMBRYO	OUTER			INNER					NON-INJECTED	INJECTED
		Nanog-Gata4-	Nanog+	TOTAL	Nanog+	Gata4+	Nanog+Gata4+	Nanog-Gata4-	TOTAL		
1	79	50	6	56	19	4	0	0	23	46	33
2	84	49	8	57	18	8	0	1	27	48	36
3	78	50	4	54	14	7	1	2	24	48	30
4	84	50	2	52	23	7	1	1	32	48	36
5	86	51	0	51	24	11	0	0	35	54	32
6	88	48	1	49	30	4	2	3	39	44	44
7	98	47	9	56	27	12	2	1	42	55	43
8	114	79	7	86	19	8	0	1	28	78	36
9	92	63	3	66	18	7	0	1	26	50	42
10	87	60	4	64	12	9	2	0	23	58	29
11	85	59	4	63	15	5	0	2	22	58	27
12	106	67	6	73	22	10	0	1	33	72	34
13	78	47	4	51	21	6	0	0	27	57	21
14	84	47	7	54	24	6	0	0	30	58	26
15	94	51	11	62	18	10	3	1	32	55	39
16	90	59	7	66	13	8	2	1	24	58	32
17	95	52	10	62	22	8	1	2	33	56	39
18	95	58	8	66	19	10	0	0	29	61	34
19	87	52	6	58	17	10	1	1	29	47	40
20	86	58	0	58	20	8	0	0	28	55	31
21	93	57	1	58	23	8	4	0	35	53	40
22	83	37	1	38	38	4	0	3	45	42	41
23	99	58	7	65	20	14	0	0	34	59	40
**TOTAL**	2065	1249	116	1365	476	184	19	21	700	1260	805
**AVERAGE**	**89.8**	**54.3**	**5.0**	**59.3**	**20.7**	**8.0**	**0.8**	**0.9**	**30.4**	**54.8**	**35.0**
SEM	1.8	1.8	0.7	2.0	1.2	0.5	0.2	0.2	1.3	1.7	1.2
Stat. sig. (exp. vs. con embryo) *p<0.05, **p<0.005	**	**		**	**				**		**
p-value (2-tailed students t-test)	2.30E-03	3.19E-07	5.51E-01	2.66E-07	3.82E-05	8.42E-01	9.38E-02	4.82E-01	2.05E-05	5.99E-02	2.07E-08
Stat. sig. (inter-clone) ‡p<0.05, ‡‡p<0.005											‡‡
p-value (2-tailed students t-test)											1.68E-11

#	NON-INJECTED CLONE								INJECTED CLONE							
	OUTER			INNER					OUTER			INNER				
	Nanog-Gata4-	Nanog+	TOTAL	Nanog+	Gata4+	Nanog+Gata4+	Nanog-Gata4-	TOTAL	Nanog-Gata4-	Nanog+	TOTAL	Nanog+	Gata4+	Nanog+Gata4+	Nanog-Gata4-	TOTAL
1	43	0	43	0	3	0	0	3	7	6	13	19	1	0	0	20
2	40	3	43	0	5	0	0	5	9	5	14	18	3	0	1	22
3	42	1	43	1	4	0	0	5	8	3	11	13	3	1	2	19
4	33	0	33	10	4	1	0	15	17	2	19	13	3	0	1	17
5	39	0	39	9	6	0	0	15	12	0	12	15	5	0	0	20
6	41	0	41	3	0	0	0	3	7	1	8	27	4	2	3	36
7	40	5	45	5	4	1	0	10	7	4	11	22	8	1	1	32
8	67	3	70	4	4	0	0	8	12	4	16	15	4	0	1	20
9	46	0	46	0	4	0	0	4	17	3	20	18	3	0	1	22
10	50	1	51	3	4	0	0	7	10	3	13	9	5	2	0	16
11	49	2	51	5	2	0	0	7	10	2	12	10	3	0	2	15
12	59	3	62	4	6	0	0	10	8	3	11	18	4	0	1	23
13	39	4	43	9	5	0	0	14	8	0	8	12	1	0	0	13
14	44	6	50	5	3	0	0	8	3	1	4	19	3	0	0	22
15	47	0	47	1	6	1	0	8	4	11	15	17	4	2	1	24
16	49	2	51	2	3	1	1	7	10	5	15	11	5	1	0	17
17	44	4	48	5	3	0	0	8	8	6	14	17	5	1	2	25
18	47	5	52	4	5	0	0	9	11	3	14	15	5	0	0	20
19	35	4	39	4	4	0	0	8	17	2	19	13	6	1	1	21
20	50	0	50	2	3	0	0	5	8	0	8	18	5	0	0	23
21	48	0	48	1	4	0	0	5	9	1	10	22	4	4	0	30
22	37	0	37	5	0	0	0	5	0	1	1	33	4	0	3	40
23	45	3	48	5	6	0	0	11	13	4	17	15	8	0	0	23
**TOTAL**	1034	46	1080	87	88	4	1	180	215	70	285	389	96	15	20	520
**AVERAGE**	**45.0**	**2.0**	**47.0**	**3.8**	**3.8**	**0.2**	**0.0**	**7.8**	**9.3**	**3.0**	**12.4**	**16.9**	**4.2**	**0.7**	**0.9**	**22.6**
SEM	1.6	0.4	1.6	0.6	0.3	0.1	0.0	0.7	0.9	0.5	1.0	1.1	0.4	0.2	0.2	1.4
Stat. sig. (exp. vs. con embryo) *p<0.05, **p<0.005	**		**	**			*	**	**		**	**			*	**
p-value (2-tailed students t-test)	1.98E-04	3.09E-01	4.09E-04	1.91E-04	7.04E-01	9.00E-01	1.26E-02	1.96E-04	3.79E-18	9.67E-01	4.67E-16	7.53E-09	5.14E-01	5.49E-02	1.46E-02	2.18E-08
Stat. sig. (inter-clone) ‡p<0.05, ‡‡p<0.005									‡‡		‡‡	‡‡		‡	‡‡	‡‡
p-value (2-tailed students t-test)									1.81E-20	1.27E-01	1.73E-19	8.85E-12	4.87E-01	4.59E-02	5.24E-04	3.70E-11

Supplementary tables ST5: Quantified cell lineage segregation in individual late blastocyst stage (E4.5) embryos cultured *in vitro* from 2-cell stage (E1.5) embryos microinjected, in a single cell, with fluorescent RDB tracer ± Tead4-specific dsRNA, (immuno-stained for Gata4 and Nanog) – *relating to figure 3 and supplementary figure S4.* The two tables report the total number of cells for each individual late blastocyst stage (E4.5) embryo, within each lineage (TE – purple, EPI – yellow, PrE – green, ICM – dark blue and total cell number – olive, plus number of outer cells that did not immuno-stain for either ICM marker – light blue, or were weakly Nanog positive – dusty blue, and inner cells that either immuno-stained for both markers – intermediate blue, or neither - grey), for embryos microinjected, in one cell at the 2-cell stage (E1.5), with either fluorescent RDB lineage tracer alone ('control 1in2 IF: Gata4/ Nanog' – upper table) or RDBs plus Tead4-dsRNA ('*Tead4* KD 1in2 IF: Gata4/ Nanog'– lower table). Additionally the total number of cells within both the non-microinjected (white) and microinjected (red) clones is also given. On the right of the tables, the contribution of cells from the non-microinjected and microinjected cell clones, for each individual embryo, to each blastocyst lineage is also given. The averaged data (s.e.m. = standard error of the mean), as used in figure 3 and supplementary figure S4, is also provided. In both control and *Tead4* KD tables, the averaged data is accompanied by p-values (resulting from 2-tailed students t-test) describing the statistical significance of inter-clone differences for each lineage plus the total cell clone count (‡ = $p<0.05$ and ‡‡ = $p<0.005$). In regard to the tabulated *Tead4* KD data, p-values (2-tailed student t-test) describing the statistical significance of differences between equivalent cell lineages and cell clones between the averaged data of control and *Tead4* KD embryos is also provided (* = $p<0.05$ and ** = $p<0.005$).

Supplementary tables ST6

Control (1in2, IF: Cdx2/ Gata4) apoptotic cells

#	TOTAL NUMBER OF CELLS					NON-INJECTED CLONE		INJECTED CLONE	
	EMBRYO	TE	ICM	NON-INJECTED	INJECTED	OUTER	ICM	OUTER	ICM
1	5	2	3	0	5	0	0	2	3
2	7	4	3	2	5	1	1	3	2
3	5	3	2	2	3	2	0	1	2
4	5	0	5	0	5	0	0	0	5
5	4	0	4	0	4	0	0	0	4
6	5	1	4	3	2	0	3	1	1
7	5	1	4	3	2	0	3	1	1
8	3	2	1	1	2	1	0	1	1
9	9	4	5	3	6	3	0	1	5
10	1	1	0	1	0	1	0	0	0
11	4	2	2	4	0	2	2	0	0
12	6	3	3	4	2	3	1	0	2
13	2	2	0	0	2	0	0	2	0
14	7	5	2	2	5	2	0	3	2
15	4	1	3	1	3	0	1	1	2
16	7	3	4	3	4	1	2	2	2
17	8	3	5	4	4	1	3	2	2
18	4	1	3	2	2	0	2	1	1
19	3	1	2	1	2	0	1	1	1
20	4	2	2	1	3	0	1	2	1
21	5	4	1	2	3	1	1	3	0
22	4	0	4	3	1	0	3	0	1
23	2	2	0	1	1	1	0	1	0
24	3	2	1	2	1	2	0	0	1
TOTAL	112	49	63	45	67	21	24	28	39
AVERAGE	4.7	2.0	2.6	1.9	2.8	0.9	1.0	1.2	1.6
SEM	0.4	0.3	0.3	0.3	0.3	0.2	0.2	0.2	0.3
Stat. sig. (inter-clone) ‡p<0.05, ‡‡p<0.005					‡				
p-value (2-tailed students t-test)					3.92E-02			3.17E-01	1.03E-01

Tead4 KD (1in2, IF: Cdx2/ Gata4) apoptotic cells

#	TOTAL NUMBER OF CELLS					NON-INJECTED CLONE		INJECTED CLONE	
	EMBRYO	TE	ICM	NON-INJECTED	INJECTED	OUTER	ICM	OUTER	ICM
1	4	1	3	1	3	0	1	1	2
2	8	6	2	0	8	0	0	6	2
3	7	4	3	3	4	3	0	1	3
4	14	8	6	4	10	2	2	6	4
5	15	10	5	1	14	1	0	9	5
6	12	9	3	5	7	4	1	5	2
7	7	1	6	2	5	1	1	0	5
8	13	7	6	4	9	2	2	5	4
9	15	12	3	0	15	0	0	12	3
10	11	8	3	1	10	1	0	7	3
11	10	3	7	5	5	1	4	2	3
12	4	3	1	1	3	1	0	2	1
13	10	5	5	6	4	3	3	2	2
14	17	6	11	4	13	1	3	5	8
15	5	3	2	0	5	0	0	3	2
16	8	5	3	1	7	0	1	5	2
17	3	1	2	1	2	0	1	1	1
18	9	5	4	4	5	2	2	3	2
19	8	7	1	1	7	1	0	6	1
20	4	1	3	2	2	0	2	1	1
21	10	6	4	2	8	1	1	5	3
22	13	8	5	4	9	3	1	5	4
23	4	4	0	1	3	1	0	3	0
24	6	4	2	2	4	1	1	3	1
TOTAL	217	127	90	55	162	29	26	98	64
AVERAGE	9.0	5.3	3.8	2.3	6.8	1.2	1.1	4.1	2.7
SEM	0.8	0.6	0.5	0.4	0.8	0.2	0.2	0.6	0.4
Stat. sig. (exp. vs. con embryo) *p<0.05, **p<0.005	**	**			**			**	*
p-value (2-tailed students t-test)	3.84E-05	3.03E-05	5.97E-02	3.59E-01	3.91E-05	2.86E-01	8.01E-01	5.03E-05	2.86E-02
Stat. sig. (inter-clone) ‡p<0.05, ‡‡p<0.005					‡‡			‡‡	‡‡
p-value (2-tailed students t-test)					7.40E-06			6.67E-05	5.88E-04

Supplementary tables ST6: Incidence of apoptotic cells within individual late blastocyst stage (E4.5) embryos *in vitro* cultured from 2-cell stage (E1.5) embryos microinjected, in a single cell, with fluorescent RDB lineage tracer ± Tead4-dsRNA, (immuno-stained for Cdx2 and Gata4) – *relating to figure 3, supplementary figures S2 and S5 and supplementary tables ST3.* The tables reports the total number of apoptotic cells (in olive), for each individual late blastocyst stage (E4.5) embryo, within each spatial compartment (*i.e.* 'TE/ outer' – light blue or encapsulated 'ICM' – dark blue), for embryos microinjected, in one cell at the 2-cell stage (E1.5), with either fluorescent RDB lineage tracer alone ('Control 1in2 apoptotic cells' – upper table) or RDBs plus Tead4-dsRNA ('Tead4 KD 1in2 apoptotic cells' – lower table) – *i.e.* those embryos referred to in figure 3, supplementary figures S2 and S5 and supplementary tables ST3. Additionally, the total number of apoptotic cells within both the non-microinjected (white) and microinjected (red) clones is also given. On the right of the tables, the incidence of apoptotic cells from the non-microinjected and microinjected cell clones, for each individual embryo, within either spatial compartment is reported. The averaged data (s.e.m. = standard error of the mean) is also provided. In both control and *Tead4* KD tables, the averaged data is accompanied by p-values (resulting from 2-tailed students t-test) describing the statistical significance of inter-clone differences for each spatial compartment (‡ = $p<0.05$ and ‡‡ = $p<0.005$). In regard to the tabulated *Tead4* KD data, p-values (2-tailed student t-test) describing the statistical significance of differences between equivalent spatial compartments and cell clones between the averaged data of control and *Tead4* KD embryos is also provided (* = $p<0.05$ and ** = $p<0.005$). Note that there is a significant increase in the general frequency of apoptosis in *Tead4* KD embryos compared to control embryos. Moreover that this is solely accounted for apoptosis in the microinjected clone and is largely restricted to the outer residing TE. Given that these cells are TE-inhibited by their nature (*i.e.* exhibit down-regulated *Tead4* expression), these data suggest a regulative developmental mechanism to remove cells unable to appropriately respond to their relative position within the embryo.

Supplementary tables ST7

Control (1in2, IF: Cdx2/ Sox17) apoptotic cells

#	TOTAL NUMBER OF CELLS					NON-INJECTED CLONE		INJECTED CLONE	
	EMBRYO	TE	ICM	NON-INJECTED	INJECTED	OUTER	ICM	OUTER	ICM
1	4	3	1	2	2	1	1	2	0
2	2	0	2	0	2	0	0	0	2
3	9	3	6	4	5	2	2	1	4
4	4	2	2	1	3	0	1	2	1
5	2	2	0	0	2	0	0	2	0
6	4	2	2	1	3	0	1	2	1
7	3	3	0	2	1	2	0	1	0
8	4	2	2	2	2	1	1	1	1
9	10	6	4	5	5	3	2	3	2
10	7	3	4	2	5	1	1	2	3
11	6	3	3	4	2	2	2	1	1
12	12	6	6	8	4	3	5	3	1
13	3	0	3	0	3	0	0	0	3
**TOTAL**	70	35	35	31	39	15	16	20	19
**AVERAGE**	**5.4**	**2.7**	**2.7**	**2.4**	**3.0**	**1.2**	**1.2**	**1.5**	**1.5**
SEM	0.9	0.5	0.5	0.6	0.4	0.3	0.4	0.3	0.4
Stat. sig. (inter-clone) ‡p<0.05, ‡‡p<0.005									
p-value (2-tailed students t-test)					4.20E-01			3.64E-01	6.59E-01

Tead4 KD (1in2, IF: Cdx2/ Sox17) apoptotic cells

#	TOTAL NUMBER OF CELLS					NON-INJECTED CLONE		INJECTED CLONE	
	EMBRYO	TE	ICM	NON-INJECTED	INJECTED	OUTER	ICM	OUTER	ICM
1	7	5	2	1	6	1	0	4	2
2	3	2	1	0	3	0	0	2	1
3	7	5	2	0	7	0	0	5	2
4	5	4	1	1	4	1	0	3	1
5	10	5	5	5	5	1	4	4	1
6	7	7	0	1	6	1	0	6	0
7	9	7	2	2	7	2	0	5	2
8	4	4	0	0	4	0	0	4	0
9	3	1	2	0	3	0	0	1	2
**TOTAL**	55	40	15	10	45	6	4	34	11
**AVERAGE**	**6.1**	**4.4**	**1.7**	**1.1**	**5.0**	**0.7**	**0.4**	**3.8**	**1.2**
SEM	0.8	0.7	0.5	0.5	0.5	0.2	0.4	0.5	0.3
Stat. sig. (exp. vs. con embryo) *p<0.05, **p<0.005					**			**	
p-value (2-tailed students t-test)	5.59E-01	5.19E-02	1.78E-01	1.46E-01	7.20E-03	2.32E-01	1.95E-01	2.40E-03	5.99E-01
Stat. sig. (inter-clone) ‡p<0.05, ‡‡p<0.005					‡‡			‡‡	
p-value (2-tailed students t-test)					9.50E-05			2.00E-04	1.61E-01

Supplementary tables ST7: Incidence of apoptotic cells within individual late blastocyst stage (E4.5) embryos *in vitro* cultured from 2-cell stage (E1.5) embryos microinjected, in a single cell, with fluorescent RDB lineage tracer ± Tead4-specific dsRNA, (immuno-stained for Cdx2 and Sox17) – *relating to figure 3, supplementary figure S3 and supplementary tables ST4.* The tables reports the total number of apoptotic cells (in olive), for each individual late blastocyst stage (E4.5) embryo, within each spatial compartment (*i.e.* 'TE/ outer' – light blue or encapsulated 'ICM' – dark blue), for embryos microinjected, in one cell at the 2-cell stage (E1.5), with either fluorescent RDB lineage tracer alone ('Control 1in2 apoptotic cells, IF: Cdx2/ Sox17' – upper table) or RDBs plus Tead4-dsRNA ('Tead4 KD 1in2 apoptotic cells, IF: Cdx2/ Sox17' – lower table) – *i.e.* those embryos referred to in figure 3, supplementary figure S3 and supplementary tables ST4. Additionally, the total number of apoptotic cells within both the non-microinjected (white) and microinjected (red) clones is also given. On the right of the tables, the incidence of apoptotic cells from the non-microinjected and microinjected cell clones, for each individual embryo, within either spatial compartment is reported. The averaged data (s.e.m. = standard error of the mean) is also provided. In both control and *Tead4* KD tables, the averaged data is accompanied by p-values (resulting from 2-tailed students t-test) describing the statistical significance of inter-clone differences for each spatial compartment (‡ = $p<0.05$ and ‡‡ = $p<0.005$). In regard to the tabulated *Tead4* KD data, p-values (2-tailed student t-test) describing the statistical significance of differences between equivalent spatial compartments and cell clones between the averaged data of control and *Tead4* KD embryos is also provided (* = $p<0.05$ and ** = $p<0.005$).

Supplementary tables ST8

Control (1in2, IF: Gata4/ Nanog) apoptotic cells

#	TOTAL NUMBER OF CELLS					NON-INJECTED CLONE		INJECTED CLONE	
	EMBRYO	TE	ICM	NON-INJECTED	INJECTED	OUTER	ICM	OUTER	ICM
1	7	3	4	2	5	1	1	2	3
2	4	1	3	2	2	0	2	1	1
3	2	0	2	1	1	0	1	0	1
4	6	4	2	3	3	2	1	2	1
5	2	1	1	1	1	0	1	1	0
6	5	1	4	3	2	1	2	0	2
7	6	3	3	5	1	2	3	1	0
8	6	5	1	5	1	5	0	0	1
9	6	1	5	3	3	1	2	0	3
10	4	2	2	2	2	1	1	1	1
11	6	2	4	4	2	1	3	1	1
12	5	2	3	2	3	1	1	1	2
13	8	4	4	5	3	3	2	1	2
14	2	1	1	1	1	0	1	1	0
15	6	3	3	5	1	3	2	0	1
16	2	0	2	1	1	0	1	0	1
17	4	1	3	2	2	0	2	1	1
18	6	3	3	3	3	1	2	2	1
19	3	0	3	1	2	0	1	0	2
20	5	1	4	2	3	0	2	1	2
21	6	2	4	5	1	2	3	0	1
22	0	0	0	0	0	0	0	0	0
23	4	4	0	3	1	3	0	1	0
24	2	1	1	1	1	1	0	0	1
25	11	6	5	7	4	2	5	4	0
**TOTAL**	11	6	5	7	4	2	5	4	0
**AVERAGE**	**4.7**	**2.0**	**2.7**	**2.8**	**2.0**	**1.2**	**1.6**	**0.8**	**1.1**
SEM	0.5	0.3	0.3	0.4	0.2	0.3	0.2	0.2	0.2
Stat. sig. (inter-clone) ‡p<0.05, ‡‡p<0.005									
p-value (2-tailed students t-test)					6.57E-02			2.66E-01	1.38E-01

Tead4 KD (1in2, IF: Gata4/ Nanog) apoptotic cells

#	TOTAL NUMBER OF CELLS					NON-INJECTED CLONE		INJECTED CLONE	
	EMBRYO	TE	ICM	NON-INJECTED	INJECTED	OUTER	ICM	OUTER	ICM
1	10	6	4	2	8	1	1	5	3
2	12	8	4	3	9	1	2	7	2
3	21	11	10	8	13	3	5	8	5
4	12	5	7	1	11	1	0	4	7
5	9	5	4	1	8	0	1	5	3
6	15	7	8	3	12	2	1	5	7
7	9	5	4	3	6	1	2	4	2
8	4	0	4	1	3	0	1	0	3
9	11	3	8	5	6	2	3	1	5
10	8	7	1	3	5	2	1	5	0
11	16	9	7	7	9	3	4	6	3
12	12	4	8	6	6	0	6	4	2
13	9	3	6	5	4	0	5	3	1
14	12	8	4	5	7	3	2	5	2
15	16	6	10	11	5	3	8	3	2
16	13	10	3	5	8	3	2	7	1
17	7	5	2	1	6	0	1	5	1
18	14	9	5	3	11	2	1	7	4
19	14	7	7	2	12	1	1	6	6
20	14	6	8	6	8	2	4	4	4
21	14	4	10	4	10	1	3	3	7
22	22	8	14	8	14	2	6	6	8
23	13	6	7	6	7	1	5	5	2
**TOTAL**	13	6	7	6	7	1	5	5	2
**AVERAGE**	**12.5**	**6.2**	**6.3**	**4.3**	**8.2**	**1.5**	**2.8**	**4.7**	**3.5**
SEM	0.8	0.5	0.6	0.5	0.6	0.2	0.4	0.4	0.5
Stat. sig. (exp. vs. con embryo) *p<0.05, **p<0.005	**	**	**	*	**		*	**	**
p-value (2-tailed students t-test)	2.81E-09	7.33E-08	1.27E-05	2.32E-02	3.40E-10	4.21E-01	1.70E-02	4.97E-10	6.97E-05
Stat. sig. (inter-clone) ‡p<0.05, ‡‡p<0.005					‡‡			‡‡	
p-value (2-tailed students t-test)					2.83E-05			3.06E-08	3.23E-01

Supplementary tables ST8: Incidence of apoptotic cells within individual late blastocyst stage (E4.5) embryos *in vitro* cultured from 2-cell stage (E1.5) embryos microinjected, in a single cell, with fluorescent RDB lineage tracer ± Tead4-specific dsRNA, (immuno-stained for Gata4 and Nanog) – *relating to figure 3, supplementary figure S4 and supplementary tables ST5.* The tables reports the total number of apoptotic cells (in olive), for each individual late blastocyst stage (E4.5) embryo, within each spatial compartment (*i.e.* 'TE/ outer' – light blue or encapsulated 'ICM' – dark blue), for embryos microinjected, in one cell at the 2-cell stage (E1.5), with either fluorescent RDB lineage tracer alone ('Control 1in2 apoptotic cells, IF: Gata4/ Nanog' – upper table) or RDBs plus Tead4-dsRNA ('Tead4 KD 1in2 apoptotic cells, IF: Gata4/ Nanog' – lower table) – *i.e.* those embryos referred to in figure 3, supplementary figure S3 and supplementary tables ST5. Additionally, the total number of apoptotic cells within both the non-microinjected (white) and microinjected (red) clones is also given. On the right of the tables, the incidence of apoptotic cells from the non-microinjected and microinjected cell clones, for each individual embryo, within either spatial compartment is reported. The averaged data (s.e.m. = standard error of the mean) is also provided. In both control and *Tead4* KD tables, the averaged data is accompanied by p-values (resulting from 2-tailed students t-test) describing the statistical significance of inter-clone differences for each spatial compartment (‡ = $p<0.05$ and ‡‡ = $p<0.005$). In regard to the tabulated *Tead4* KD data, p-values (2-tailed student t-test) describing the statistical significance of differences between equivalent spatial compartments and cell clones between the averaged data of control and *Tead4* KD embryos is also provided (* = $p<0.05$ and ** = $p<0.005$).

Supplementary tables ST9

Control (1in4, IF: Cdx2/Gata4)

#	TOTAL NUMBER OF CELLS							NON-INJECTED CLONE				INJECTED CLONE			
	EMBRYO	TE	ICM			NON-INJECTED	INJECTED	OUTER	INNER			OUTER	INNER		
			EPI	PrE	TOTAL				Gata4 -	Gata4 +	TOTAL		Gata4 -	Gata4 +	TOTAL
1	86	65	11	10	21	62	24	49	8	5	13	16	3	5	8
2	94	70	19	5	24	70	24	49	16	5	21	21	3	0	3
3	104	83	14	7	21	88	16	74	10	4	14	9	4	3	7
4	97	75	17	5	22	78	19	61	13	4	17	14	4	1	5
5	101	76	16	9	25	73	28	58	10	5	15	18	6	4	10
6	97	71	17	9	26	70	27	53	12	5	17	18	5	4	9
7	99	68	24	7	31	72	27	46	19	7	26	22	5	0	5
8	101	82	10	9	19	75	26	56	10	9	19	26	0	0	0
9	95	75	13	7	20	68	27	53	11	4	15	22	2	3	5
10	98	70	19	9	28	71	27	50	12	9	21	20	7	0	7
11	103	83	7	13	20	71	32	56	3	12	15	27	4	1	5
12	105	78	17	10	27	77	28	57	15	5	20	21	2	5	7
13	84	59	17	8	25	51	33	38	11	2	13	21	6	6	12
14	105	76	17	12	29	76	29	58	8	10	18	18	9	2	11
15	101	77	20	4	24	79	22	62	14	3	17	15	6	1	7
16	80	61	12	7	19	54	26	39	9	6	15	22	3	1	4
17	115	97	11	7	18	83	32	67	9	7	16	30	2	0	2
18	108	88	14	6	20	76	32	60	12	4	16	28	2	2	4
19	111	91	12	8	20	81	30	67	6	8	14	24	6	0	6
**TOTAL**	1884	1445	287	152	439	1375	509	1053	208	114	322	392	79	38	117
**AVERAGE**	**99.2**	**76.1**	**15.1**	**8.0**	**23.1**	**72.4**	**26.8**	**55.4**	**10.9**	**6.0**	**16.9**	**20.6**	**4.2**	**2.0**	**6.2**
SEM	2.0	2.3	0.9	0.5	0.9	2.1	1.0	2.1	0.8	0.6	0.8	1.2	0.5	0.5	0.7

Tead4 KD (1in4, IF: Cdx2/Gata4)

#	TOTAL NUMBER OF CELLS							NON-INJECTED CLONE				INJECTED CLONE			
	EMBRYO	TE	ICM			NON-INJECTED	INJECTED	OUTER	INNER			OUTER	INNER		
			EPI	PrE	TOTAL				Gata4 -	Gata4 +	TOTAL		Gata4 -	Gata4 +	TOTAL
1	104	72	25	7	32	77	27	65	7	5	12	7	18	2	20
2	78	47	18	13	31	58	20	38	11	9	20	9	7	4	11
3	87	62	16	9	25	69	18	53	9	7	16	9	7	2	9
4	95	66	15	14	29	69	26	60	4	5	9	6	11	9	20
5	75	50	20	5	25	59	16	45	10	4	14	5	10	1	11
6	82	61	12	9	21	59	23	53	3	3	6	8	9	6	15
7	100	71	19	10	29	75	25	55	12	8	20	16	7	2	9
8	97	61	22	14	36	81	16	55	14	12	26	6	8	2	10
9	92	68	13	11	24	67	25	59	4	4	8	9	9	7	16
10	75	44	27	4	31	56	19	43	9	4	13	1	18	0	18
11	105	83	12	10	22	84	21	73	6	5	11	10	6	5	11
12	96	69	20	7	27	71	25	58	8	5	13	11	12	2	14
13	84	58	20	6	26	67	17	57	8	2	10	1	12	4	16
14	89	65	14	10	24	70	19	53	9	8	17	12	5	2	7
15	102	76	14	12	26	76	26	55	11	10	21	21	3	2	5
16	88	57	20	11	31	65	23	53	7	5	12	4	13	6	19
17	98	73	19	6	25	80	18	68	7	5	12	5	12	1	13
18	95	62	26	7	33	70	25	52	12	6	18	10	14	1	15
19	108	76	14	18	32	86	22	61	9	16	25	15	5	2	7
20	78	47	27	4	31	55	23	42	13	0	13	5	14	4	18
21	80	55	19	6	25	59	21	46	10	3	13	9	9	3	12
22	87	55	25	7	32	67	20	49	13	5	18	6	12	2	14
23	78	57	13	8	21	53	25	41	9	3	12	16	4	5	9
24	75	56	14	5	19	59	16	49	6	4	10	7	8	1	9
25	91	71	11	9	20	69	22	58	5	6	11	13	6	3	9
26	89	60	14	15	29	66	23	42	11	13	24	18	3	2	5
27	105	69	25	11	36	81	24	62	10	9	19	7	15	2	17
28	110	78	23	9	32	95	15	70	18	7	25	8	5	2	7
29	108	81	16	11	27	89	19	69	10	10	20	12	6	1	7
**TOTAL**	2651	1850	533	268	801	2032	619	1584	265	183	448	266	268	85	353
**AVERAGE**	**91.4**	**63.8**	**18.4**	**9.2**	**27.6**	**70.1**	**21.3**	**54.6**	**9.1**	**6.3**	**15.4**	**9.2**	**9.2**	**2.9**	**12.2**
SEM	2.1	1.9	0.9	0.6	0.9	2.0	0.7	1.7	0.6	0.7	1.0	0.9	0.8	0.4	0.8
Stat. sig. (exp. vs. con embryo) *p<0.05, **p<0.005	*	**	*		**		**					**	**		**
p-value (2-tailed students t-test)	1.02E-02	2.00E-04	1.72E-02	1.43E-01	7.00E-04	4.33E-01	1.00E-04	7.70E-01	8.95E-02	7.27E-01	2.44E-01	3.45E-09	1.81E-06	1.29E-01	1.72E-06

Supplementary tables ST9: Quantified cell lineage segregation in individual late blastocyst stage (E4.5) embryos cultured *in vitro* from 4-cell stage (E2.0) embryos microinjected, in a single cell, with fluorescent RDB tracer ± Tead4-dsRNA, (immuno-stained for Cdx2 and Gata4) *– relating to supplementary figure S6.* The two tables report the total number of cells for each individual late blastocyst stage (E4.5) embryo, within each lineage (TE – light blue, EPI – yellow, PrE – green, ICM – dark blue and total cell number - olive), for embryos microinjected, in one cell at the 4-cell stage (E2.0), with either fluorescent RDB lineage tracer alone ('Control 1in4' – upper table) or RDBs plus Tead4-dsRNA ('*Tead4* KD 1in 4' – lower table). Additionally the total number of cells within both the non-microinjected (white) and microinjected (red) clones is also given. On the right of the tables, the contribution of cells from the non-microinjected and microinjected cell clones, for each individual embryo, to each blastocyst lineage is also given. The averaged data (s.e.m. = standard error of the mean), as used in supplementary figure S6, is also provided. In regard to the tabulated *Tead4* KD data, p-values (2-tailed student t-test) describing the statistical significance of differences between equivalent cell lineages and cell clones between the averaged data of control and *Tead4* KD embryos is also provided (* = p<0.05 and ** = p<0.005).

Supplementary tables ST10

Control (1in4, IF: Cdx2/ Gata4) apoptotic cells

#	TOTAL NUMBER OF CELLS					NON-INJECTED CLONE		INJECTED CLONE	
	EMBRYO	TE	ICM	NON-INJECTED	INJECTED	OUTER	ICM	OUTER	ICM
1	2	1	1	1	1	0	1	1	0
2	4	1	3	2	2	0	2	1	1
3	2	2	0	1	1	1	0	1	0
4	5	2	3	4	1	2	2	0	1
5	4	4	0	2	2	2	0	2	0
6	5	2	3	3	2	2	1	0	2
7	9	4	5	7	2	3	4	1	1
8	4	2	2	3	1	2	1	0	1
9	6	2	4	4	2	1	3	1	1
10	4	2	2	3	1	2	1	0	1
11	10	9	1	10	0	9	1	0	0
12	2	1	1	2	0	1	1	0	0
13	1	1	0	1	0	1	0	0	0
14	4	0	4	1	3	0	1	0	3
15	2	2	0	2	0	2	0	0	0
16	5	0	5	3	2	0	3	0	2
17	3	1	2	3	0	1	2	0	0
18	0	0	0	0	0	0	0	0	0
19	4	2	2	3	1	2	1	0	1
TOTAL	76	38	38	55	21	31	24	7	14
AVERAGE	4.0	2.0	2.0	2.9	1.1	1.6	1.3	0.4	0.7
SEM	0.6	0.5	0.4	0.5	0.2	0.5	0.3	0.1	0.2

Tead4 KD (1in4, IF: Cdx2/Gata4) apoptotic cells

#	TOTAL NUMBER OF CELLS					NON-INJECTED CLONE		INJECTED CLONE	
	EMBRYO	TE	ICM	NON-INJECTED	INJECTED	OUTER	ICM	OUTER	ICM
1	3	0	3	2	1	0	2	0	1
2	9	6	3	5	4	3	2	3	1
3	5	4	1	3	2	3	0	1	1
4	7	1	6	4	3	1	3	0	3
5	6	2	4	5	1	1	4	1	0
6	12	8	4	10	2	7	3	1	1
7	2	2	0	1	1	1	0	1	0
8	7	3	4	4	3	2	2	1	2
9	8	5	3	6	2	5	1	0	2
10	2	0	2	1	1	0	1	0	1
11	4	3	1	3	1	3	0	0	1
12	4	1	3	2	2	1	1	0	2
13	18	10	8	13	5	10	3	0	5
14	8	6	2	4	4	3	1	3	1
15	6	3	3	4	2	1	3	2	0
16	7	5	2	3	4	3	0	2	2
17	5	3	2	0	5	0	0	3	2
18	5	2	3	0	5	0	0	2	3
19	4	3	1	1	3	1	0	2	1
20	7	4	3	4	3	3	1	1	2
21	5	5	0	4	1	4	0	1	0
22	9	5	4	7	2	4	3	1	1
23	2	2	0	0	2	0	0	2	0
24	9	6	3	6	3	4	2	2	1
25	5	2	3	2	3	1	1	1	2
26	3	0	3	2	1	0	2	0	1
27	6	1	5	3	3	0	3	1	2
28	3	1	2	2	1	1	1	0	1
29	3	3	0	1	2	1	0	2	0
TOTAL	174	96	78	102	72	63	39	33	39
AVERAGE	6.0	3.3	2.7	3.5	2.5	2.2	1.3	1.1	1.3
SEM	0.6	0.4	0.3	0.5	0.2	0.4	0.2	0.2	0.2
Stat. sig. (exp. vs. con embryo)<br>*p<0.05, **p<0.005	*	*			**			**	*
p-value (2-tailed students t-test)	2.24E-02	4.91E-02	1.88E-01	4.15E-01	1.00E-04	3.97E-01	8.18E-01	1.60E-03	4.00E-02

Supplementary tables ST10: Incidence of apoptotic cells within individual late blastocyst stage (E4.5) embryos *in vitro* cultured from 4-cell stage (E2.0) embryos microinjected, in a single cell, with fluorescent RDB lineage tracer ± Tead4-dsRNA, (immuno-stained for Cdx2 and Gata4) – *relating to supplementary figures S5 and S6 and supplementary tables ST9.* The table reports the total number of apoptotic cells (in olive), for each individual late blastocyst stage (E4.5) embryo, within each spatial compartment (*i.e.* 'TE/ outer' – light blue or encapsulated 'ICM' – dark blue), for embryos microinjected, in one cell at the 4-cell stage (E2.0), with either fluorescent RDB lineage tracer alone ('Control 1in4 apoptotic cells' – upper table) or RDBs plus Tead4-dsRNA ('Tead4 KD 1in4 apoptotic cells' – lower table) – *i.e.* those embryos referred to in supplementary figures S5 and S6 and supplementary tables ST9. Additionally, the total number of apoptotic cells within both the non-microinjected (white) and microinjected (red) clones is also given. On the right of the tables, the incidence of apoptotic cells from the non-microinjected and microinjected cell clones, for each individual embryo, within either spatial compartment is reported. The averaged data (s.e.m. = standard error of the mean) is also provided. In regard to the tabulated *Tead4* KD data, p-values (2-tailed student t-test) describing the statistical significance of differences between equivalent spatial compartments and cell clones between the averaged data of control and *Tead4* KD embryos is also provided (* = $p<0.05$ and ** = $p<0.005$). Note that there is a significant increase in the general frequency of apoptosis in *Tead4* KD embryos compared to control embryos. Moreover that this is solely accounted for apoptosis in the microinjected clone and is largely restricted to the outer residing TE. Given that these cells are TE-inhibited by their nature, these data suggest a regulative developmental mechanism to remove cells unable to appropriately respond to their relative position within the embryo.

Supplementary tables ST11

Control (1+8, IF: Cdx2/Gata4)

#	TOTAL NUMBER OF CELLS							NON-INJECTED CLONE				INJECTED CLONE			
	EMBRYO	TE	ICM			NON-INJECTED	INJECTED	OUTER	INNER			OUTER	INNER		
			EPI	PrE	TOTAL				Gata4 -	Gata4 +	TOTAL		Gata4 -	Gata4 +	TOTAL
1	87	67	10	10	20	75	12	60	6	9	15	7	4	1	5
2	84	60	20	4	24	75	9	55	16	4	20	5	4	0	4
3	64	49	11	4	15	52	12	41	9	2	11	8	2	2	4
4	87	69	14	4	18	80	7	65	12	3	15	4	2	1	3
5	97	68	25	4	29	84	13	57	24	3	27	11	1	1	2
6	100	82	9	9	18	85	15	72	6	7	13	10	3	2	5
7	97	78	12	7	19	84	13	74	4	6	10	4	8	1	9
8	106	82	16	8	24	99	7	75	16	8	24	7	0	0	0
9	80	69	4	7	11	66	14	57	4	5	9	12	0	2	2
10	99	73	18	8	26	88	11	62	18	8	26	11	0	0	0
11	90	69	17	4	21	79	11	59	16	4	20	10	1	0	1
12	89	67	14	8	22	79	10	63	8	8	16	4	6	0	6
13	75	62	9	4	13	67	8	57	6	4	10	5	3	0	3
14	116	100	7	9	16	100	16	84	7	9	16	16	0	0	0
15	87	70	9	8	17	79	8	62	9	8	17	8	0	0	0
16	82	66	8	8	16	72	10	56	8	8	16	10	0	0	0
17	86	64	18	4	22	78	8	56	18	4	22	8	0	0	0
18	86	73	8	5	13	78	8	67	6	5	11	6	2	0	2
19	109	89	11	9	20	97	12	83	7	7	14	6	4	2	6
20	108	86	10	12	22	92	16	70	10	12	22	16	0	0	0
21	89	74	8	7	15	77	12	66	4	7	11	8	4	0	4
22	99	84	10	5	15	89	10	74	10	5	15	10	0	0	0
23	110	91	10	9	19	101	9	85	9	7	16	6	1	2	3
24	83	57	10	16	26	68	15	48	8	12	20	9	2	4	6
25	87	75	3	9	12	69	18	68	0	1	1	7	3	8	11
26	79	59	13	7	20	65	14	55	6	4	10	4	7	3	10
27	84	65	13	6	19	76	8	61	9	6	15	4	4	0	4
28	94	77	12	5	17	80	14	66	10	4	14	11	2	1	3
29	83	69	10	4	14	74	9	63	7	4	11	6	3	0	3
30	100	73	14	13	27	89	11	69	10	10	20	4	4	3	7
TOTAL	2737	2167	353	217	570	2397	340	1930	283	184	467	237	70	33	103
**AVERAGE**	**91.2**	**72.2**	**11.8**	**7.2**	**19.0**	**79.9**	**11.3**	**64.3**	**9.4**	**6.1**	**15.6**	**7.9**	**2.3**	**1.1**	**3.4**
SEM	2.1	2.0	0.9	0.5	0.9	2.1	0.5	1.8	0.9	0.5	1.0	0.6	0.4	0.3	0.6

Tead4 KD (1+8, IF: Cdx2/Gata4)

#	TOTAL NUMBER OF CELLS							NON-INJECTED CLONE				INJECTED CLONE			
	EMBRYO	TE	ICM			NON-INJECTED	INJECTED	OUTER	INNER			OUTER	INNER		
			EPI	PrE	TOTAL				Gata4 -	Gata4 +	TOTAL		Gata4 -	Gata4 +	TOTAL
1	87	58	25	4	29	79	8	58	18	3	21	0	7	1	8
2	92	71	14	7	21	85	7	66	12	7	19	5	2	0	2
3	85	62	18	5	23	69	16	54	11	4	15	8	7	1	8
4	89	73	7	9	16	79	10	70	5	4	9	3	2	5	7
5	91	74	13	4	17	83	8	70	9	4	13	4	4	0	4
6	78	58	10	10	20	70	8	56	6	8	14	2	4	2	6
7	71	58	8	5	13	64	7	57	2	5	7	1	6	0	6
8	81	62	14	5	19	70	11	62	3	5	8	0	11	0	11
9	84	61	19	4	23	76	8	60	12	4	16	1	7	0	7
10	99	75	15	9	24	92	7	71	12	9	21	4	3	0	3
11	102	77	15	10	25	90	12	68	12	10	22	9	3	0	3
12	92	74	14	4	18	85	7	71	10	4	14	3	4	0	4
13	103	84	14	5	19	87	16	79	8	0	8	5	6	5	11
14	86	69	11	6	17	78	8	69	3	6	9	0	8	0	8
15	79	52	23	4	27	71	8	50	18	3	21	2	5	1	6
16	71	49	18	4	22	64	7	46	16	2	18	3	2	2	4
17	74	52	14	8	22	66	8	52	7	7	14	0	7	1	8
TOTAL	1464	1109	252	103	355	1308	156	1059	164	85	249	50	88	18	106
**AVERAGE**	**86.1**	**65.2**	**14.8**	**6.1**	**20.9**	**76.9**	**9.2**	**62.3**	**9.6**	**5.0**	**14.6**	**2.9**	**5.2**	**1.1**	**6.2**
SEM	2.4	2.5	1.2	0.6	1.0	2.2	0.7	2.2	1.2	0.6	1.2	0.7	0.6	0.4	0.6
Stat. sig. (exp. vs. con embryo) *p<0.05, **p<0.005		*	*				*					**	**		**
p-value (2-tailed students t-test)	1.17E-01	3.41E-02	4.03E-02	1.38E-01	1.61E-01	3.35E-01	2.20E-02	4.83E-01	8.89E-01	1.66E-01	5.73E-01	2.14E-06	5.02E-04	9.36E-01	2.30E-03

Supplementary tables ST11: Quantified cell lineage segregation in individual late blastocyst stage (E4.5) embryos derived from 8-cell stage (E2.6) chimeric embryos consisting of a single control (fluorescent RDB tracer alone) or *Tead4* KD (fluorescent RDB tracer plus Tead4-dsRNA) cell (immuno-stained for Cdx2 and Gata4) – *relating to figure 4 and supplementary figure S7.* The two tables report the total number of cells for each individual late blastocyst stage (E4.5) chimeric embryo, within each lineage (TE – light blue, EPI – yellow, PrE – green, ICM – dark blue and total cell number - olive), for embryo chimeras generated by aggregating one control (fluorescent RDB tracer alone – 'Control 1+8' – upper table) or one *Tead4* KD (fluorescent RDB tracer alone plus Tead4-dsRNA – '*Tead4* KD 1+8' – lower table) cell with non-manipulated 8-cell stage (E2.6) embryos. Additionally the total number of cells within both the non-microinjected/ non-marked (white) and microinjected/ marked (red) clones is also given. On the right of the tables, the contribution of cells from the non-marked and marked cell clones, for each individual embryo, to each blastocyst lineage is also given. The averaged data (s.e.m. = standard error of the mean), as used in figure 4 and supplementary figure S7, is also provided. In regard to the tabulated *Tead4* KD data, p-values (2-tailed student t-test) describing the statistical significance of differences between equivalent cell lineages and cell clones between the averaged data of control and *Tead4* KD embryos is also provided (* = $p<0.05$ and ** = $p<0.005$).

Supplementary tables ST12

Control (1+8, IF: Cdx2/ Gata4) apoptotic cells

#	TOTAL NUMBER OF CELLS					NON-INJECTED CLONE		INJECTED CLONE	
	EMBRYO	TE	ICM	NON-INJECTED	INJECTED	OUTER	ICM	OUTER	ICM
1	6	3	3	5	1	3	2	0	1
2	6	3	3	3	3	1	2	2	1
3	8	4	4	8	0	4	4	0	0
4	8	5	3	6	2	5	1	0	2
5	3	1	2	0	3	0	0	1	2
6	4	3	1	4	0	3	1	0	0
7	2	2	0	2	0	2	0	0	0
8	2	0	2	2	0	0	2	0	0
9	7	4	3	6	1	3	3	1	0
10	3	2	1	2	1	1	1	1	0
11	4	2	2	3	1	1	2	1	0
12	2	0	2	1	1	0	1	0	1
13	4	0	4	3	1	0	3	0	1
14	5	0	5	5	0	0	5	0	0
15	8	5	3	7	1	4	3	1	0
16	8	7	1	7	1	6	1	1	0
17	9	6	3	5	4	3	2	3	1
18	6	3	3	4	2	2	2	1	1
19	7	5	2	6	1	4	2	1	0
20	7	0	7	7	0	0	7	0	0
21	3	1	2	2	1	1	1	0	1
22	2	1	1	0	2	0	0	1	1
23	10	3	7	8	2	3	5	0	2
24	7	2	5	4	3	1	3	1	2
25	18	7	11	9	9	5	4	2	7
26	8	5	3	6	2	5	1	0	2
27	7	7	0	7	0	7	0	0	0
28	5	3	2	3	2	1	2	2	0
29	5	1	4	4	1	1	3	0	1
30	14	3	11	9	5	3	6	0	5
TOTAL	188	88	100	138	50	69	69	19	31
**AVERAGE**	**6.3**	**2.9**	**3.3**	**4.6**	**1.7**	**2.3**	**2.3**	**0.6**	**1.0**
SEM	0.6	0.4	0.5	0.5	0.3	0.4	0.3	0.1	0.3

Tead4 KD (1+8, IF: Cdx2/ Gata4) apoptotic cells

#	TOTAL NUMBER OF CELLS					NON-INJECTED CLONE		INJECTED CLONE	
	EMBRYO	TE	ICM	NON-INJECTED	INJECTED	OUTER	ICM	OUTER	ICM
1	8	3	5	5	3	3	2	0	3
2	3	2	1	3	0	2	1	0	0
3	10	3	7	8	2	3	5	0	2
4	7	4	3	4	3	3	1	1	2
5	11	3	8	6	5	0	6	3	2
6	8	3	5	1	7	1	0	2	5
7	1	1	0	1	0	1	0	0	0
8	6	0	6	5	1	0	5	0	1
9	5	3	2	1	4	0	1	3	1
10	9	6	3	8	1	6	2	0	1
11	9	4	5	6	3	1	5	3	0
12	12	8	4	7	5	3	4	5	0
13	4	1	3	2	2	0	2	1	1
14	10	3	7	9	1	3	6	0	1
15	10	4	6	8	2	3	5	1	1
16	8	7	1	8	0	7	1	0	0
17	11	4	7	8	3	3	5	1	2
TOTAL	132	59	73	90	42	39	51	20	22
**AVERAGE**	**7.8**	**3.5**	**4.3**	**5.3**	**2.5**	**2.3**	**3.0**	**1.2**	**1.3**
SEM	0.7	0.5	0.6	0.7	0.5	0.5	0.5	0.4	0.3
Stat. sig. (exp. vs. con embryo) *p<0.05, **p<0.005									
p-value (2-tailed students t-test)	1.37E-01	4.08E-01	2.17E-01	4.08E-01	1.80E-01	9.92E-01	2.69E-01	1.83E-01	5.45E-01

Supplementary tables ST12: Incidence of apoptotic cells within individual late blastocyst stage (E4.5) embryos derived from 8-cell stage (E2.6) chimeric embryos consisting of a single control (fluorescent RDB tracer alone) or *Tead4* KD (fluorescent RDB tracer plus Tead4-dsRNA) cell (immuno-stained for Cdx2 and Gata4) – *relating to figure 4, supplementary figures S5 and S7 and supplementary tables ST11.* The table reports the total number of apoptotic cells (in olive), for each individual late blastocyst stage (E4.5) embryos, within each spatial compartment (*i.e.* 'TE/ outer' – light blue or encapsulated 'ICM – dark blue), for embryo chimeras generated by aggregating one control (fluorescent RDB tracer alone – 'control 1+8' – upper table) or one *Tead4* KD (fluorescent RDB tracer alone plus Tead4-dsRNA – '*Tead4* KD 1+8' – lower table) cell with non-manipulated 8-cell stage (E2.6) embryos – *i.e.* those embryos referred to in figure 4, supplementary figures S5 and S7 and supplementary tables ST11. Additionally, the total number of apoptotic cells within both the non-microinjected/ non-marked (white) and microinjected/ marked (red) clones is also given. On the right of the tables, the incidence of apoptotic cells from the non-marked and marked cell clones, for each individual embryo, within either spatial compartment is reported. The averaged data (s.e.m. = standard error of the mean) is also provided. In regard to the tabulated *Tead4* KD data, p-values (2-tailed student t-test) describing the statistical significance of differences between equivalent spatial compartments and cell clones between the averaged data of control- and *Tead4* KD-chimeric embryos is also provided (* = p<0.05 and ** = p<0.005). Note that there are no significant differences in the incidence of apoptosis between control or *Tead4* KD embryos, irrespective of spatial location or non-marked or marked cell clones.

Supplementary table ST13

UNPERTURBED *IN VITRO* CONTROL EMBRYOS (2-CELL to E4.5)					
	TOTAL NUMBER OF CELLS				
#	EMBRYO	TE	ICM		
			EPI	PrE	TOTAL
1	80	63	13	4	17
2	88	67	12	9	21
3	83	60	19	4	23
4	73	63	4	6	10
5	82	58	12	12	24
6	96	74	10	12	22
7	115	89	19	7	26
8	113	91	11	11	22
9	97	77	10	10	20
10	96	68	21	7	28
11	113	75	23	15	38
12	103	78	15	10	25
13	98	76	14	8	22
14	128	104	14	10	24
15	83	62	12	9	21
16	86	70	12	4	16
**TOTAL**	1534	1175	221	138	359
**AVERAGE**	**95.9**	**73.4**	**13.8**	**8.6**	**22.4**
SEM	3.8	3.1	1.2	0.8	1.5

Supplementary table ST13: Quantified cell lineage segregation in individual late blastocyst stage (E4.5) embryos cultured *in vitro* from the 2-cell stage (E1.5) – *relating to supplementary figure S12*. A total of 16 embryos were recovered at the 2-cell (E1.5) stage and *in vitro* cultured until the late blastocyst stage (E4.5). The embryos were then fixed and immuno-stained for Cdx2 (a TE marker) and Gata4 (a PrE marker), to distinguish the three blastocyst lineages (the EPI lineage cells were defined as cells negative for both Cdx2 and Gata4 immuno-staining). The number of cells within each blastocyst lineage (plus the overall ICM cell count; defined as EPI + PrE) was then calculated. The table reports the number of cells for each individual embryo, within each lineage (TE – light blue, EPI – yellow, PrE – green and ICM – dark blue), together with the overall cell count (olive). The average number of cells for each lineage, as used in supplementary figure S12, is also given (s.e.m. = standard error of the mean). Quantifying this data provided a convenient experimental baseline for appropriate *in vitro* embryo culture development, against which experimental embryo (in which control or *Tead4* KD cell clones of various sizes were generated) could be compared. Therefore, permitting the exclusion of embryos from our analysis that exhibited developmental defects that could be attributed to general experimental manipulation (*see also materials and methods*).

Supplementary tables ST14

Primary antibodies used

#	ANTIGEN	cat. no.	supplier	species raised in and clonicity	dilution used	seconday antibody used (refer to below table)
1	Nanog	ab80892	Abcam	rabbit, polyclonal	1 in 200	E
2	Tead4	ab58310	Abcam	mouse, monoclonal	1 in 200	C
3	Nanog	14-5761	Affymetrix/eBioscience	rat, monoclonal	1 in 100	B
4	Cdx2	MU392A-UC	BioGenex	mouse, monoclonal	1 in 200	A,C
5	Cdh1	3195	Cell Signalling	rabbit, polyclonal	1 in 500	E
6	phospho-ezrin (pERM)	3149	Cell Signalling	rabbit, monoclonal	1 in 500	E
7	Sox17	AF1924	R&D Systems	goat, polyclonal	1 in 100	H
8	Fgfr2	sc-122	Santa Cruz	rabbit, polyclonal	1 in 200	F
9	Gata4	sc-9053	Santa Cruz	rabbit, polyclonal	1 in 100	E,G
10	Pard6b	sc-67393	Santa Cruz	rabbit, polyclonal	1 in 100	E
11	Prkcz	sc-216	Santa Cruz	rabbit, polyclonal	1 in 200	E
12	Scrib	sc-28737	Santa Cruz	rabbit, polyclonal	1 in 100	E
13	Yap1	sc101199	Santa Cruz	mouse, monoclonal	1 in 100	A,C,D

Secondary fluorescent conjugated antibodies used

#	SPECIES OF ANTIBODY TARGETED	cat. no.	supplier	species raised in and fluorophore	dilution used	used in combination with primary antibody (refer to above table)
A	mouse	715-605-150	Jackson Immuno Research Inc.	donkey, Alexa647	1 in 500	5,13
B	rat	712-096-150	Jackson Immuno Research Inc.	donkey, FITC	1 in 400	3
C	mouse	A-11029	Life Technologies	goat, Alexa488	1 in 500	2,4,13
D	mouse	A-21424	Life Technologies	goat, Alexa555	1 in 500	13
E	rabbit	A-21206	Life Technologies	donkey, Alexa488	1 in 500	1,5,6,9-12
F	rabbit	A-21429	Life Technologies	goat, Alexa555	1 in 500	8
G	rabbit	A-31573	Life Technologies	donkey, Alexa647	1 in 500	9
H	goat	A-21222	Life Technoilogies	rabbit, Alex488	1 in 500	7

Supplementary tables ST14: Details of the primary and primary antibodies used in confocal-based immuno-fluorescent microscopy.

Supplementary table ST15

Oligonucleotide primer pairs used			
#	Gene	forward (5'-3')	reverse (5'-3')
1	Cdx2	TCAAGAAGAAGCAGCAGCAG	GCAAGGAGGTCACAGGACTC
2	Dab2	GTCGGGGATTGGCTGGTATC	GGCCATTGGTTGTGCTTGTT
3	Fgfr2	AAGAGGGACACAGGATGGAC	TGTGGGTCTCTGTGAGGGTA
4	Gata3	CCGAAACCGGAAGATGTCTA	AGATGTGGCTCAGGGATGAC
5	H2afz	GCGCAGCCATCCTGGAGTA	CCGATCAGCGATTTGTGGA
6	Lrp2	TGGTCAGTGTGTTCCCATCG	CGTGTTATAGCAGGCTCCGT
7	Nanog	GGTTGAAGACTAGCAATGGTCTGA	TGCAATGGATGCTGGGATACTC
8	Rpl23	CCAGCAGTGGTAATTCGACA	GCAGAGCCTTTCATCTCTCC
9	Tead4	GAGCCCGGAGAACATGATTA	CCAAATGAGCAGACCTTCGT

Supplementary table ST15: Q-RTPCR gene-specific oligonucleotide primer sequences.
